# Genetic Landscape of the ACE2 Coronavirus Receptor

**DOI:** 10.1161/CIRCULATIONAHA.121.057888

**Published:** 2022-04-07

**Authors:** Zhijian Yang, Erin Macdonald-Dunlop, Jiantao Chen, Ranran Zhai, Ting Li, Anne Richmond, Lucija Klarić, Nicola Pirastu, Zheng Ning, Chenqing Zheng, Yipeng Wang, Tingting Huang, Yazhou He, Huiming Guo, Kejun Ying, Stefan Gustafsson, Bram Prins, Anna Ramisch, Emmanouil T. Dermitzakis, Grace Png, Niclas Eriksson, Jeffrey Haessler, Xiaowei Hu, Daniela Zanetti, Thibaud Boutin, Shih-Jen Hwang, Eleanor Wheeler, Maik Pietzner, Laura M. Raffield, Anette Kalnapenkis, James E. Peters, Ana Viñuela, Arthur Gilly, Sölve Elmståhl, George Dedoussis, John R. Petrie, Ozren Polašek, Lasse Folkersen, Yan Chen, Chen Yao, Urmo Võsa, Erola Pairo-Castineira, Sara Clohisey, Andrew D. Bretherick, Konrad Rawlik, GenOMICC Consortium, IMI-DIRECT Consortium, Tõnu Esko, Stefan Enroth, Åsa Johansson, Ulf Gyllensten, Claudia Langenberg, Daniel Levy, Caroline Hayward, Themistocles L. Assimes, Charles Kooperberg, Ani W. Manichaikul, Agneta Siegbahn, Lars Wallentin, Lars Lind, Eleftheria Zeggini, Jochen M. Schwenk, Adam S. Butterworth, Karl Michaëlsson, Yudi Pawitan, Peter K. Joshi, J. Kenneth Baillie, Anders Mälarstig, Alexander P. Reiner, James F. Wilson, Xia Shen

**Affiliations:** 1 Biostatistics Group, School of Life Sciences, Sun Yat-sen University, Guangzhou, China (Z.Y., J.C., R.Z., T.L., Z.N., C.Z., Y.W., X.S.).; 2 State Key Laboratory of Genetic Engineering, School of Life Sciences, Fudan University, Shanghai, China (X.S.).; 3 Center for Intelligent Medicine Research, Greater Bay Area Institute of Precision Medicine (Guangzhou), Fudan University, China (Z.Y., J.C., R.Z., T.L., X.S.).; 4 Centre for Global Health Research, Usher Institute, University of Edinburgh, UK (E.M.-D., N.P., Y.H., P.K.J., J.F.W., X.S.).; 5 MRC Human Genetics Unit, Institute of Genetics and Cancer, University of Edinburgh, Western General Hospital, UK (A. Richmond, L.K., T.B., E.P.-C., A.D.B., C.H., J.F.W.).; 6 Human Technopole Viale Rita Levi-Montalcini, Milan, Italy (N.P.).; 7 Department of Medical Epidemiology and Biostatistics, Karolinska Institutet, Stockholm, Sweden (Z.N., T.H., Y.C., Y.P., A.M., X.S.).; 8 West China School of Public Health, West China Fourth Hospital, Sichuan University, Chengdu (Y.H.).; 9 Department of Cardiac Surgery, Guangdong Cardiovascular Institute, Guangdong Provincial People’s Hospital Guangdong Academy of Medical Sciences, Guangzhou, China (H.G.).; 10 Division of Genetics, Department of Medicine, Brigham and Women’s Hospital and Harvard Medical School, Boston, MA (K.Y.).; 11 T.H. Chan School of Public Health, Harvard University, Boston, MA (K.Y.).; 12 Department of Immunology and Inflammation, Imperial College London, UK (J.E.P.).; 13 British Heart Foundation Cardiovascular Epidemiology Unit, Department of Public Health and Primary Care, University of Cambridge, UK (B.P., J.E.P., A.S.B.).; 14 Health Data Research UK Cambridge, Wellcome Genome Campus and University of Cambridge (B.P., J.E.P., A.S.B.).; 15 Department of Genetic Medicine and Development, University of Geneva Medical School, Switzerland (A. Ramisch, E.T.D., A.V.).; 16 Institute of Translational Genomics, Helmholtz Zentrum München–German Research Center for Environmental Health, Neuherberg, Germany (G.P., A.G., E.Z.).; 17 Technical University of Munich (TUM), School of Medicine, Germany (G.P.).; 18 Uppsala Clinical Research Center, Sweden (N.E.).; 19 Division of Public Health Sciences, Fred Hutchinson Cancer Research Center, Seattle, WA (J.H., C.K., A.P.R.).; 20 Center for Public Health Genomics, University of Virginia, Charlottesville (X.H., A.W.M.).; 21 Department of Medicine, Division of Cardiovascular Medicine, Stanford University School of Medicine, CA (D.Z., T.L.A.).; 22 Stanford Cardiovascular Institute, Stanford University, CA (D.Z., T.L.A.).; 23 Framingham Heart Study, MA (S.-J.H., C.Y., D.L.).; 24 MRC Epidemiology Unit, University of Cambridge, UK (E.W., M.P., C.L.).; 25 Department of Genetics, University of North Carolina at Chapel Hill (L.M.R.).; 26 Estonian Genome Centre, Institute of Genomics, University of Tartu, Estonia (A.K., U.V., T.E.).; 27 Institute of Molecular and Cell Biology, University of Tartu, Estonia (A.K.).; 28 Biosciences Institute, Faculty of Medical Sciences, Newcastle University, UK (A.V.).; 29 Pfizer Worldwide Research, Development and Medical, Stockholm, Sweden (A.M.).; 30 Wellcome Sanger Institute, Wellcome Genome Campus, Hinxton, Cambridge, UK (A.G., E.Z.).; 31 Faculty of Medicine, Lund University, Sweden (S. Elmståhl).; 32 Department of Nutrition and Dietetics, School of Health Science and Education, Harokopio University of Athens, Greece (G.D.).; 33 Institute of Cardiovascular & Medical Sciences, University of Glasgow, UK (J. Petrie).; 34 University of Split School of Medicine, Croatia (O.P.).; 35 Danish National Genome Center, Copenhagen, Denmark (L.F.).; 36 Population Sciences Branch, Division of Intramural Research, National Heart, Lung, and Blood Institute, National Institutes of Health, Bethesda, MD (S.-J.H., C.Y., D.L.).; 37 Roslin Institute, University of Edinburgh, Easter Bush, UK (E.P.-C., S.C., K.R., J.K.B.).; 38 Algebra University College, Ilica, Zagreb, Croatia (O.P.).; 39 Department of Immunology, Genetics and Pathology, Uppsala Universitet, Science for Life Laboratory, Sweden (S. Enroth, A.J., U.G.).; 40 Department of Medical Sciences, Uppsala University, Sweden (A.S., S.G., L.W., L.L.).; 41 Technical University of Munich (TUM) and Klinikum Rechts der Isar, TUM School of Medicine, Germany (E.Z.).; 42 Affinity Proteomics, Science for Life Laboratory, KTH Royal Institute of Technology, Solna, Sweden (J.M.S.).; 43 British Heart Foundation Centre of Research Excellence, University of Cambridge, UK (A.S.B.).; 44 National Institute for Health Research Blood and Transplant Research Unit in Donor Health and Genomics, University of Cambridge, UK (A.S.B.).; 45 Department of Surgical Sciences, Uppsala University, Sweden (K.M.).; 46 Intensive Care Unit, Royal Infirmary of Edinburgh, UK (J.K.B.).; 47 Computational Medicine, Berlin Institute of Health at Charité–Universitätsmedizin, Germany (M.P., C.L.).

**Keywords:** angiotensin-converting enzyme 2, Genome-Wide Association Study, cardiovascular diseases, COVID-19, SARS-CoV-2

## Abstract

SARS-CoV-2, the causal agent of COVID-19, enters human cells using the ACE2 (angiotensin-converting enzyme 2) protein as a receptor. ACE2 is thus key to the infection and treatment of the coronavirus. ACE2 is highly expressed in the heart and respiratory and gastrointestinal tracts, playing important regulatory roles in the cardiovascular and other biological systems. However, the genetic basis of the ACE2 protein levels is not well understood.

**Methods:**

We have conducted the largest genome-wide association meta-analysis of plasma ACE2 levels in >28 000 individuals of the SCALLOP Consortium (Systematic and Combined Analysis of Olink Proteins). We summarize the cross-sectional epidemiological correlates of circulating ACE2. Using the summary statistics–based high-definition likelihood method, we estimate relevant genetic correlations with cardiometabolic phenotypes, COVID-19, and other human complex traits and diseases. We perform causal inference of soluble ACE2 on vascular disease outcomes and COVID-19 severity using mendelian randomization. We also perform in silico functional analysis by integrating with other types of omics data.

**Results:**

We identified 10 loci, including 8 novel, capturing 30% of the heritability of the protein. We detected that plasma ACE2 was genetically correlated with vascular diseases, severe COVID-19, and a wide range of human complex diseases and medications. An X-chromosome cis–protein quantitative trait loci–based mendelian randomization analysis suggested a causal effect of elevated ACE2 levels on COVID-19 severity (odds ratio, 1.63 [95% CI, 1.10–2.42]; *P*=0.01), hospitalization (odds ratio, 1.52 [95% CI, 1.05–2.21]; *P*=0.03), and infection (odds ratio, 1.60 [95% CI, 1.08–2.37]; *P*=0.02). Tissue- and cell type–specific transcriptomic and epigenomic analysis revealed that the ACE2 regulatory variants were enriched for DNA methylation sites in blood immune cells.

**Conclusions:**

Human plasma ACE2 shares a genetic basis with cardiovascular disease, COVID-19, and other related diseases. The genetic architecture of the ACE2 protein is mapped, providing a useful resource for further biological and clinical studies on this coronavirus receptor.

**Clinical Perspective**

**What Is New?**

- The overall heritability of the ACE2 (angiotensin-converting enzyme 2) level is 16%, of which 30% can be explained by 10 protein quantitative trait loci identified in this study.
- ACE2 level is genetically correlated with both COVID-19 and cardiovascular disease.
- Elevated ACE2 levels show a causal relationship with COVID-19 severity, hospitalization, and infection, as shown by a cis–protein quantitative trait loci–based mendelian randomization analysis.
- ACE2 regulatory variants are enriched on DNA methylation sites in blood immune cells.

**What Are the Clinical Implications?**

- The causal evidence for ACE2 suggests that pharmacological inhibition of circulating ACE2 may be a promising approach for treating COVID-19 or its comorbidities.
- Transcription factors such as HNF1A and HNF4A play essential roles in ACE2 regulation and could provide alternative paths to pharmacological modulation of ACE2 plasma levels.
- The genetic correlations between ACE2 levels and both COVID-19 and cardiovascular disease risk imply that the cardiovascular complications seen in patients with COVID-19 may be intrinsic to the disease and mechanistically driven by ACE2.

SARS-CoV-2, the causal agent of COVID-19, enters human cells using the ACE2 (angiotensin-converting enzyme 2) protein as a receptor.^1–5^ ACE2 is highly expressed in the heart, respiratory, and gastrointestinal tracts, and it plays important regulatory roles in the cardiovascular and other biological systems.^6^ ACE2 proteolytically degrades angiotensin II (a potent vasoconstrictive, proinflammatory, and prothrombotic mediator) into angiotensin-(1–7), thereby regulating blood pressure, salt and water balance, glucose homeostasis, and amino acid absorption in the kidney and small intestine.

Shedding of a soluble form of ACE2 from the cell surface is regulated by membrane-bound enzymes such as TMPRSS2 and ADAM17.^7,8^ Enzymatic cleavage of the ACE2 extracellular domain by TMPRSS2 after binding of the spike protein of SARS-CoV-2 to ACE2 also plays a s role in SARS-CoV-2 cell entry and infection. To date, several large observational cohort studies including either patients with heart failure or atrial fibrillation or healthy children and adults have demonstrated that circulating ACE2 antigen levels^9–12^ are higher in men than women, increase with age, and correlate with cardiovascular outcomes and cardiometabolic and inflammatory biomarkers, but not with the use of angiotensin-converting enzyme (ACE) inhibitors or angiotensin receptor blockers (ARBs).^13,14^ Therefore, further understanding the epidemiological relationships and genetic regulation of the soluble ACE2 protein could have important implications for risk of SARS-CoV-2 infection or disease severity and cardiovascular disease (CVD) risk in general and could motivate further assessment of the causal role of ACE2 in these diseases.

A recent genome-wide analysis of soluble ACE2 measured in plasma of 3442 patients with heart failure identified 3 genome-wide significant protein quantitative trait loci (pQTL): a cis-pQTL on chromosome X (located near the cognate ACE2 gene) and 2 trans-loci on chromosomes 12 and 21 encompassing the genes encoding transcription factors HNF1A and ERG, respectively.^15^ Current antibody-based ACE2 pQTL studies have been insufficient to capture enough heritability of the protein or to estimate downstream genetic connections between ACE2 and diseases such as CVD and COVID-19.

Here, by performing so far the largest genome-wide association meta-analysis of plasma ACE2 (n=28 204), we summarize the cross-sectional epidemiological correlates of circulating ACE2; report 10 pQTL, including 8 novel for ACE2; estimate relevant genetic correlations with other cardiometabolic phenotypes; and conduct causal inference of soluble ACE2 on vascular disease outcomes and COVID-19 severity using mendelian randomization (MR).

## Methods

### Transparency and Openness Promotion

The summary statistics data of the plasma ACE2 genome-wide association study (GWAS) meta-analysis are publicly available online.^16^ Other summary-level data used in this study are also available as stated in Data Availability in the Supplemental Material. The individual-level human phenotypic and genetic data and biological samples used in this study will not be made available to other researchers for the purposes of reproducing the results or replicating the procedure.

### Data Collection

Across the participating cohorts, plasma ACE2 abundance was measured with the Proximity Extension Assay (PEA) technology of Olink Proteomics on the Multiplex CVDII targeted 96-protein panel. PEA has high readout specificity and sensitivity (Supplemental Material) and consumes a minimal amount of sample. In PEA, matched pairs of oligonucleotide-labeled antibodies bind to the target ACE2 antigen, and on antibody binding, the matched oligonucleotides are brought into proximity.^17^ A polymerase chain reaction target sequence corresponding to the ACE2 protein is then created, amplified, and quantified by quantitative polymerase chain reaction.

Genome-wide association summary statistics of plasma ACE2 protein were obtained from 14 cohorts of European ancestry. The cohorts’ details are given in Table S1 and the Supplemental Material. The institutional review committees for each cohort approved the study methodology, and all study participants provided informed consent for their clinical and genetic data to be used for research. The maximum sample size per single nucleotide polymorphism (SNP) is 28 204 individuals. Each cohort provided data imputed to 1000 Genomes Project phase 3 reference or to the Haplotype Reference Consortium reference, which resulted in 17 166 011 autosomal SNPs and 3 922 856 X chromosome SNPs. We tested 8 682 405 and 730 046 genetic variants on the autosomes and X chromosome, respectively, with a minor allele frequency >0.01. Each cohort applied quality control measures for call rate filters, sex mismatch, population outliers, heterozygosity, and cryptic relatedness as documented in the Supplemental Material. Before we ran the genetic analyses, protein values (on the log2 scale) were inverse-gaussian transformed to zero mean and unit variance. No detection threshold was applied to the raw Olink measurement values.

The summary association statistics for severe COVID-19 and other complex traits and diseases were obtained from publicly available established resources (see Data Availability in Supplemental Material). The ACE protein serum concentration GWAS in 4147 individuals from the ORIGIN trial (Outcome Reduction With Initial Glargine Intervention)^18^ was obtained from the authors (see also Acknowledgments). The aptamer-based plasma ACE-level GWAS made use of the recent study in 35 559 Icelanders.^19^ For severe COVID-19, we considered the GenOMICC Consortium GWAS.^20^ For COVID-19 hospitalization and infection GWAS, we used COVID-19 Host Genetics Initiative GWAS meta-analysis round 4 (October 20, 2020).^21^ To provide a proper replication of causal effect inference, we subtracted the GenOMICC GWAS from the Host Genetics Initiative meta-analysis using the R package MetaSubtract.

### Genome-Wide Association Meta-Analysis

Genetic analyses were conducted with additive model regressions with adjustment for population structure and study-specific parameters (details in Supplemental Material). For the X chromosome, the analysis was performed separately for men and women, with 0 to 1 genotype coding for men. Each contributing cohort uploaded the result summary statistics in a standardized format to a secure computational cluster at the University of Edinburgh. The meta-analysis was performed with METAL (2011-03-25)^22^ with the inverse variance–weighted approach (STDERR option).

### Heritability and Genetic Correlation Analysis

For the autosomes, we estimated the genome-wide SNP-based heritability and genetic correlation values for plasma ACE2 and other complex traits using high-definition likelihood,^23^ an approach based on summary association statistics. We used the default reference panel containing linkage disequilibrium (LD) information of 1 029 876 quality-controlled imputed SNPs provided in the high-definition likelihood software. The genetic variance captured by the cis-pQTL of ACE2 on chromosome X was estimated from the top SNP assuming Hardy-Weinberg equilibrium, that is, $2f(1-f){\hat{\beta }}^{2},$ where f and $\hat{\beta }$ are the minor allele frequency and the genetic effect estimate, respectively. The genetic variance captured by each autosomal trans-pQTL was also estimated from the top SNP assuming Hardy-Weinberg equilibrium. Because the phenotypic variance of ACE2 was standardized to 1 through inverse-gaussian transformation, the sum of $2f\left(1-f\right){\hat{\beta }}^{2}$ across all the pQTL yields the proportion of phenotypic variance explained by the discovered pQTL. Thus, the ratio of the genetic variance captured by the pQTL to the estimated heritability gives the proportion of heritability explained.

### Mendelian Randomization

For cis-pQTL–based MR analysis, we used SNP genotypes within the X chromosomal *ACE2* gene region as instrumental variables to perform a standard inverse variance–weighted causal effect estimation using the TwoSampleMR package.^24^ The LD *r*^2^ clumping threshold was set to 0.001 to ensure independently associated genetic instruments. For autosomal genome-wide multi-instrument MR analysis, we conducted the analysis using the generalized summary data–based MR (GSMR)^25^ module in the GCTA software. The genome-wide significance threshold of *P* values was set to 5×10^−^^8^. The LD *r*^2^ clumping threshold was set to 0.05.

### Cis-Expression Quantitative Trait Loci Analysis

To analyze the effect of any identified plasma ACE2 pQTL on nearby gene expression, we used 2 well-established, publicly available genetics of gene expression databases: the large (n>31 000 individuals) blood gene expression eQTLGen and the GTEx Consortium, which is smaller (n≈1000 individuals) but contains a much broader tissue representation (54 nondiseased tissue sites) to detect tissue-specific expression quantitative trait loci (eQTL). For each discovered trans-pQTL of plasma ACE2, we extracted the eQTL association summary statistics for the genes within a 1-Mb window of the lead ACE2-associated variant. The analysis was performed to evaluate candidate genes for the discovered ACE2 pQTL, excluding the major histocompatibility complex region on chromosome 6.

### Chromatin States Enrichment Analysis

We extracted 127 consolidated epigenomes from the Roadmap Epigenomics Project,^26^ which covers 15 chromatin states quantified across 28 human tissues and cell types. We used an SNP-based logistic regression to test for the enrichment of LD-corrected genome-wide significant ACE2 association signals on each chromatin state in each tissue or cell type:


$$\log\;\;\left(\frac{E\left[Pr\left(P_{j}<5\times 10^{-8}\right)\right]}{E\left[Pr\left(P_{j}\;\geq 5\times 10^{-8}\right)\right]}\right)=\mu +\delta l_{j}+\beta C_{j}$$ (1)

where is the LD score of the *j*th SNP, precalculated by the ldsc software for the HapMap3 SNPs (major histocompatibility complex region excluded); *C*_*j*_ takes a value of 0 or 1, as an indicator for whether the SNP is annotated to be within the particular chromatin state; and β is the parameter of interest, that is, the log odds ratio of significant ACE2 associations in the chromatin state compared with that in the other SNPs. The higher enrichment of the ACE2 association signals is at the annotated SNPs, the higher β will be.

### Statistical Analysis

The genetic effects from different cohorts were inverse-variance weighted in the meta-analysis using the METAL software. Subtraction of cohorts from GWAS summary statistics was done with the R package MetaSubtract. The heritability and genetic correlation parameters were estimated with the high-definition likelihood and ldsc software. MR analysis was performed with the R package TwoSampleMR and GCTA software. Linear and generalized linear models were fitted using the lm() and glm() procedures in R. The displayed parameters estimates are shown with SEs or 95% CIs.

## Results

### Epidemiological Correlates of Plasma ACE2 Levels

Across our SCALLOP (Systematic and Combined Analysis of Olink Proteins) cohorts, plasma ACE2 levels were higher in men than in women (Table S2). Significant positive associations were found between higher plasma ACE2 and cathepsin L1, body mass index (BMI), triglycerides, liver fat, hypertension, CVD, blood pressure, and diabetes (Figure S1 and Table S3). Among commonly used antihypertensive drug classes, calcium channel blockers were positively associated with ACE2 levels, but there was no significant correlation between ACE2 levels and either ACE inhibitor use or angiotensin receptor blocker use in the overall sample. When the sample was stratified by sex, ARB use was significantly associated with higher ACE2 levels in men only. We also identified significant sex heterogeneity for the associations with cathepsin L1, BMI, triglycerides, and diabetes: The ACE2 associations with BMI and triglycerides were stronger in men, whereas the associations with cathepsin L1 and diabetes appeared to be stronger in women.

### GWAS Identified 10 Genome-Wide Significant Loci for Plasma ACE2

We conducted a genome-wide association meta-analysis in up to 28 204 individuals from 14 cohorts in the SCALLOP Consortium for plasma ACE2 protein measured with the Olink platform. Our analysis included both the autosomes and the X chromosome, in which the *ACE2* gene is encoded. Genotypes in each cohort were either obtained from whole-genome sequencing or imputed to the 1000 Genomes Phase 3 or Haplotype Reference Consortium reference panels (see Supplemental Material). The ACE2 measurements were inverse-gaussian transformed before analysis, adjusted for age, sex, population structure, and cohort-specific covariates. We tested 8 682 405 and 730 046 genetic variants on the autosomes and the X chromosome, respectively, with minor allele frequencies >0.01.

**Figure 1.**
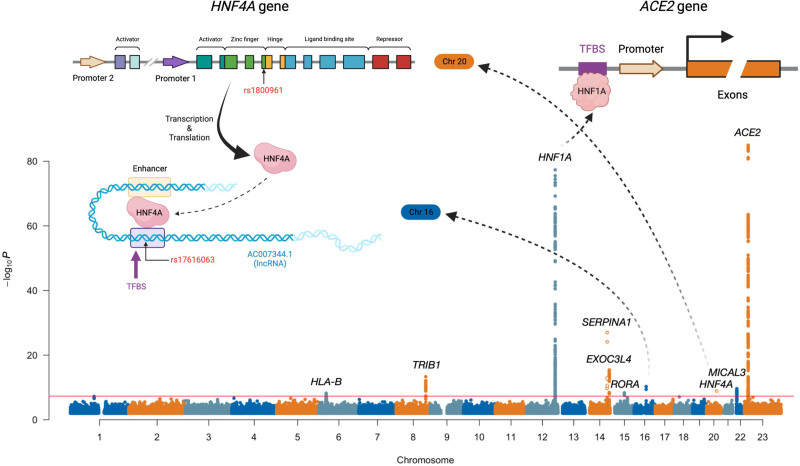
**Genomic meta-analysis scan of plasma ACE2.** Mapped genes are labeled at genome-wide significant loci (*P*<5×10^−8^). Genome-wide significant variants with minor allele frequency <0.05 are marked as circles instead of solid dots. Illustrations are provided for the interactions between 2 pairs of mapped loci; the locus on chromosome 16 is a transcription binding site for the transcription factor HNF4A mapped on chromosome 20, and HNF1A acts as the transcription factor for the *ACE2* gene. ACE2 indicates angiotensin-converting enzyme 2; and TFBS, transcription factor binding site.

The genome-wide association meta-analysis discovered 10 genome-wide significant loci for plasma ACE2 (Table, Figure 1, and Figures S2–S12). From conditional and joint multi-SNP analysis^27^ on these discovered loci, we identified a secondary genome-wide significant association at the *EXOC3L4* locus on chromosome 14 (lead variant rs73356643, GWAS *P*=1.3×10^−6^, conditional *P*=1.0*×*10^−8^). Using the high-definition likelihood method,^23^ we estimated the autosomal SNP-based heritability of plasma ACE2 to be 16.1% (SE, 2.5%). Adding the X chromosome heritability of 0.5% for men and 1.3% for women (Figure S13), we obtained an estimated genome-wide heritability of 16.6% and 17.4% for men and women, respectively. According to an additive genetic effects model under Hardy-Weinberg equilibrium, the 10 lead variants together explain 4.1% of the phenotypic variance of plasma ACE2 equivalent to ≈30% of the heritability.

**Table.**
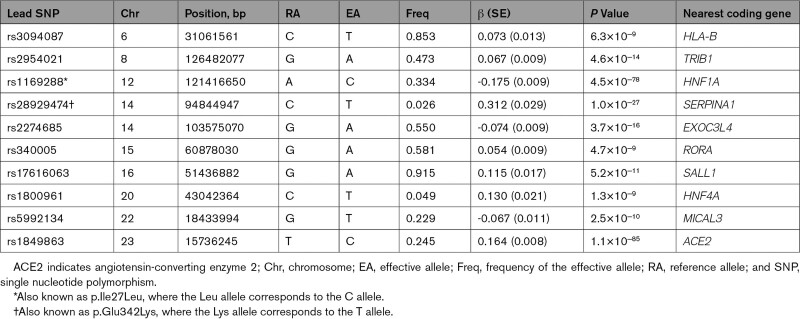
Summary of 10 Genome-Wide Significant Loci for Plasma ACE2

Except for the *ACE2* cis-pQTL (lead variant rs1849863, *P*=1.1×10^−85^) and the *ACE2* transcription factor *HNF1A* trans-pQTL (lead variant rs1169288, *P*=4.5×10^−78^), the other 8 trans-pQTL have not been previously associated with plasma ACE2 (Table and Table S4). In contrast to ACE2 and HNF1A pQTL, we were unable to replicate another previously reported locus on chromosome 21 (rs2186346, *P*=0.41).^15^

To further characterize the potential functional, biological, and clinical impact of our newly discovered ACE2-associated loci and to prioritize plausible causal genes at these loci, we cross-referenced each of our index SNPs using (1) 2 available gene expression and eQTL resources, the eQTLGen Consortium (Figure S14A and Table S5) and the GTEx consortium (Figure S14B and Table S6); (2) epigenomic information from the Roadmap Epigenomics Project,^26^ which covers 15 chromatin states quantified across various human tissues and cell types (Figure S15 and Table S7); and (3) genotype-phenotype association database records from PhenoScanner^28^ (Table S8). We excluded the HLA locus on chromosome 6 because of the complicated genomic and LD structure in this region.

### Tissue, Epigenomic, and Regulatory Characterization of the ACE2-Associated Genomic Loci

Across the 8 autosomal ACE2-associated genomic loci, analysis of blood gene expression associations using eQTLGen data showed that plausible candidate genes generally ranked high among the genes underlying each trans-pQTL. However, multitissue analysis of the GTEx eQTL associations of the prioritized genes in the discovered pQTL at each of the 8 loci did not point to clear specific tissues of ACE2 regulation. In GTEx, *ACE2* cis-eQTL associations also were found in multiple tissues, including brain, tibial nerve, tibial artery, and pituitary (Figure S16), although *ACE2* tends to have specifically lower expression in these tissues (Figure S17). Across diverse tissues and cell types, chromatin states such as weak transcription and heterochromatin harbored enriched plasma ACE2 genetic associations (Figure S15). Notably, the enrichment was more tissue specific for the strong transcription chromatin state in which the ACE2 genetic associations were particularly enriched in T cells, natural killer cells, hematopoietic cells, and blood lymphoblastoid cells. This suggests a shared genetic regulation between plasma ACE2 and hemocyte immune functions.

### Functional and Phenotypic Annotation of the ACE2-Associated Loci

The majority of the autosomal trans-pQTL for ACE2 have been pleiotropically associated through previous GWAS with various cardiovascular, metabolic, inflammatory/immune, and pulmonary traits (Table S8). These loci are detailed further below.

Two loci involve coding variants from the same family of hepatic/pancreatic transcription factor genes. These include missense variants of *HNF1A* (lead variant rs1169288 or p.Ile27Leu, *P*=4.5×10^−78^) and hepatocyte nuclear factor *HNF4A* (lead variant rs1800961, p.Thr139Ile, *P*=1.3×10^−9^). Both of these *HNF1A* and *HNF4A* missense variants have previously been associated with high-density lipoprotein, low-density lipoprotein, total cholesterol, type 2 diabetes, coronary heart disease, C-reactive protein (CRP), fibrinogen, coagulation factor VII, and other hepatically synthesized enzymes or enzyme inhibitors such as γ-glutamyl transferase or α1-antitrypsin levels. In particular, p.Ile27Leu is a well-known variant with the Leu allele (corresponds to the C allele of rs1169288), increasing the risk of type 2 diabetes; meanwhile, it reduces plasma ACE2 concentration (β=−0.175; SE=0.009) in our study. Rare loss-of-function variants of *HNF1A* and *HNF4A* can cause the mendelian disorder mature-onset diabetes of the young. The transcription factors HNF1A and HNF4A have been shown to regulate ACE2 expression in a pancreas- and ileum-specific manner, respectively.^29,30^ Both HNF1A and HNF4A also have a crucial role in protein fucosylation.^31^ Given that the SARS-CoV-2 spike (S) protein is heavily fucosylated with host-derived glycans,^32^ in addition to regulating the expression of ACE2, these 2 loci could potentially influence S protein glycosylation and thereby influence the entry of the virus to the host cell.^3^

In addition to the 2 HNF transcription factors, 2 other loci contain genes involved in hepatic gene transcriptional regulation. *RORA* encodes retinoic acid receptor-α, another hepatic transcriptional activator involved in the regulation of circadian rhythm, metabolism, and immune function.^33^ The intronic *RORA* variant (lead variant rs340005, *P*=4.7×10^−9^) is located in an ENCODE distal enhancer region and has previously been associated with CRP and γ-glutamyl transferase levels. The lead SNP rs2954021 (*P*=4.6×10^−14^) located upstream of the pleiotropic *TRIB1* gene has been associated with various metabolic traits, liver enzymes, plasma lipids, kidney function, blood cell traits, hepatic steatosis, and coronary heart disease (Figure S18 shows examples of colocalization of the ACE2 associations with high-density lipoprotein, low-density lipoprotein, BMI, and waist circumference). The protein product of *TRIB1*, Tribbles-1, posttranslationally regulates the degradation of CCAAT/enhancer-binding protein α, which contributes to the dysregulation of hepatic lipid-related gene expression^34^ and is also a positive regulator of HNF4A.^35^

The lead variant at the *SERPINA1* locus, rs28929474 (*P*=1.0×10^−27^), encodes the canonical European Pi*Z allele p.Glu342Lys, causing recessively inherited α-1-antitrypsin deficiency, a disease that affects the lung and liver.^36^ The Lys allele leads to lower serum concentrations of α-1-antitrypsin^37^ and higher plasma ACE2 levels (β=0.312; SE=0.029). In population-based genetic studies, rs28929474 has been associated with various metabolic phenotypes (height, bone mineral density, systolic blood pressure, fat-free mass, gallstones, lipids), pulmonary function, alcohol consumption, and plasma levels of various hepatic enzymes (alkaline phosphatase, alanine aminotransferase) and acute-phase proteins synthesized in the liver (eg, CRP, AFP). An important point is that the α-1-antitrypsin serine protease inhibitor additionally possesses antiviral and anti-inflammatory properties, including inhibition of TMPRSS2 and ADAM17 enzyme activities, and therefore has been proposed as a possible host protective factor against COVID-19.^38,39^ The class 1 major histocompatibility complex locus (lead variant rs3094087, *P*=6.3×10^−9^) has been associated with a variety of autoimmune, gastrointestinal, blood cell counts, anthropometric, and pulmonary traits.

Several of the newly identified pQTL have less clearly understood biological relationships to plasma ACE2 or are less clearly mapped with respect to causal genes. The *MICAL3* intronic lead variant rs5992134 (*P*=2.5×10^−10^) is a cis-eQTL for *MICAL3*, is located within a predicted distal enhancer region, and has previously been associated with liver enzymes, pulmonary function, and systolic blood pressure. MICAL-3 is an NADPH-dependent oxidoreductase enzyme that participates in actin cytoskeleton reorganization.^40^ The pQTL on chromosome 16 is located in a gene desert (closest gene, *SALL1*, ≈300 kb) but has previously been associated with CRP and red blood cell count. The lead variant rs17616063 is located within an ENCODE distal enhancer and overlaps a transcription factor binding site (Ensembl regulatory feature identifier, ENSR00000085879), with the nearest experimentally verified motif ENSM00156191351 binding with protein HNF4A (encoded on the chromosome 20 locus) in the HepG2 cell line (Figure 1 and Figure S19). The chromosome 14 locus (*EXOC3L4* intronic lead variant rs2274685, *P*=3.7×10^−16^) has been associated with liver enzymes, platelet quantitative traits, and gene expression of *CDC42BPB*, whereas another nearby gene, *TNFAIP2*, is abundantly expressed in immune cells and has been implicated in inflammation and infectious disease.^41^ From conditional and joint multi-SNP analyses of these discovered loci, we identified a secondary genome-wide significant association at the *EXOC3L4* locus on chromosome 14 (lead variant rs73356643, GWAS *P*=1.3×10^−6^, conditional *P*=1.0×10^−8^).

At the cis-pQTL located near the *ACE2* gene, the lead variant rs1849863 was strongly associated with plasma ACE2 levels (*P*=1.1×10^−85^). With a threshold of LD *r*^2^<0.001, we obtained another 2 independent cis-instruments rs143380244 (*P*=8.8×10^−9^) and rs73202884 (*P*=2.8×10^−13^) for plasma ACE2. The 3 cis variants together capture 1.4% of the phenotypic variance of ACE2, equivalent to 8.8% of the heritability of ACE2. It is notable that no established clinical or phenotypic association for the ACE2 cis-regulatory locus was found, possibly because of the lack of association studies on the X chromosome (Figure S20). Our sentinel SNP at the cis-pQTL rs1849863 is in strong LD with the previously reported top SNP rs12558179 by Nelson et al^15^ (*R*^2^=0.9489 and *D*′=0.9741 in 1000 Genomes European individuals). A recent report identified a cis-regulatory rare variant rs190509934 for *ACE2* gene expression that influences COVID-19 risk,^42^ for which the rare allele C only forms a haplotype with the minor, C, allele of our detected top SNP rs1849863. However, the rare allele of rs190509934 was reported by Horowitz et al^42^ to reduce ACE2 mRNA expression, whereas we found that the minor allele of rs1849863 increases the plasma ACE2 level.

### Genetic and Causal Relationships Between Plasma ACE2 and CVDs

**Figure 2.**
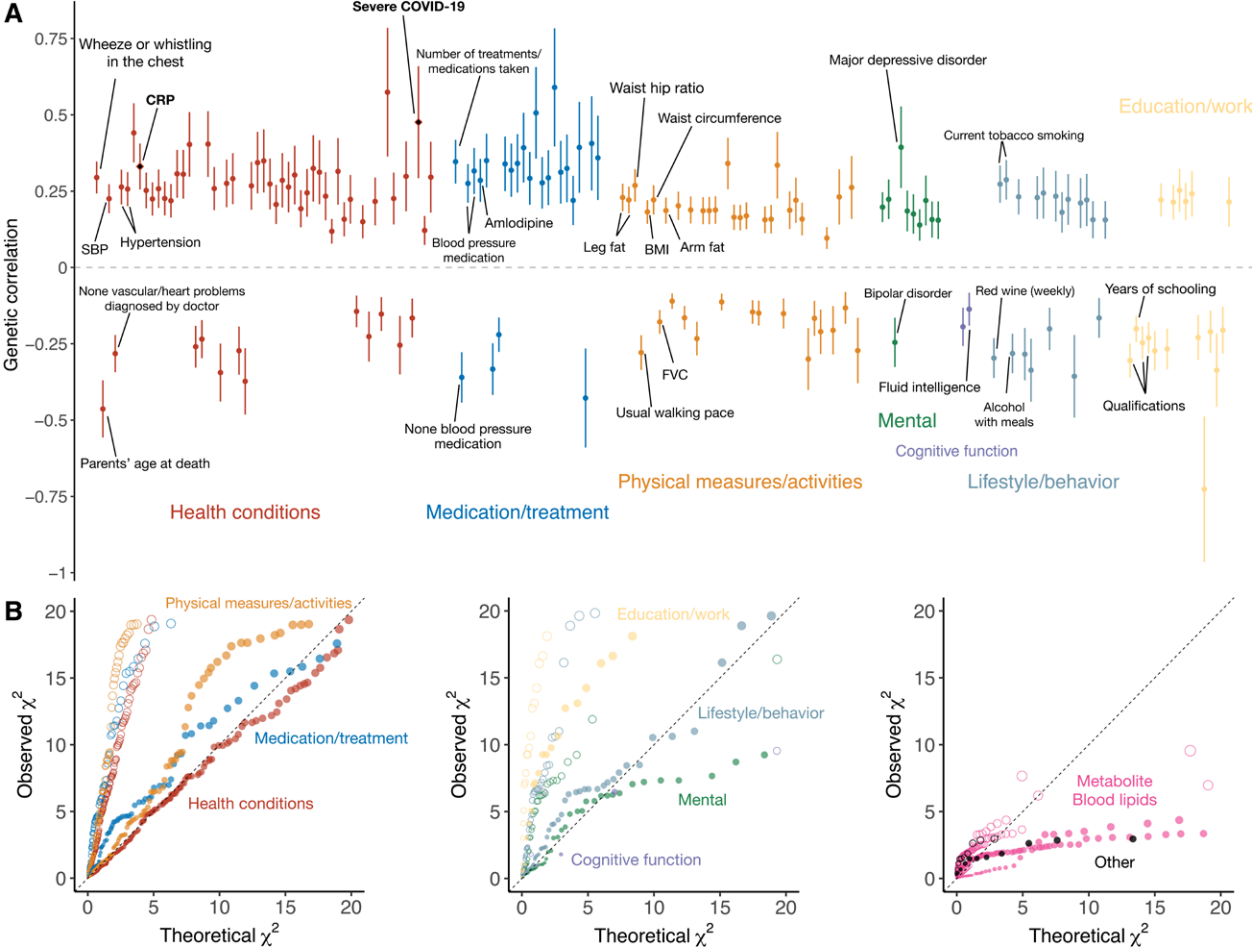
**Genetic correlations between plasma ACE2 and human complex traits and diseases. A**, Statistically significant (false discovery rate <5%) genetic correlations with ACE2 (angiotensin-converting enzyme 2) are shown; severe COVID-19, C-reactive protein (CRP), and other representative traits are labeled. Error bars represent SEs. Colors label different groups of phenotypes. Detailed explanations of the annotated phenotypes are given in the Supplemental Material. **B**, Enrichment of genetic correlations with ACE2 within each group of phenotypes. Circles are the quantile-quantile (QQ) plots of the genetic correlations test statistics against the null, whereas the solid dots are the QQ plots of the test statistics within each phenotype group against all the analyzed phenotypes. BMI indicates body mass index; FVC, forced vital capacity; and SBP, systolic blood pressure.

We explored the shared genetic architecture between plasma ACE2 and cardiovascular and metabolic risk factors and CVD outcomes. We estimated bivariate ACE2-trait genetic correlations using 2 different databases of GWAS summary statistics.^43^ First, we assessed genetic correlations between plasma ACE2 and various human lifestyle/behavioral, psychosocial, health-related, and biomarker traits with available GWAS summary statistics using the high-definition likelihood method^23^ (Table S9). Among these, plasma ACE2 showed significant positive genetic correlations with cigarette smoking, blood pressure, cholesterol levels, CRP, and anthropometric traits (false discovery rate <5%; Figure 2 and Table S9).

**Figure 3.**
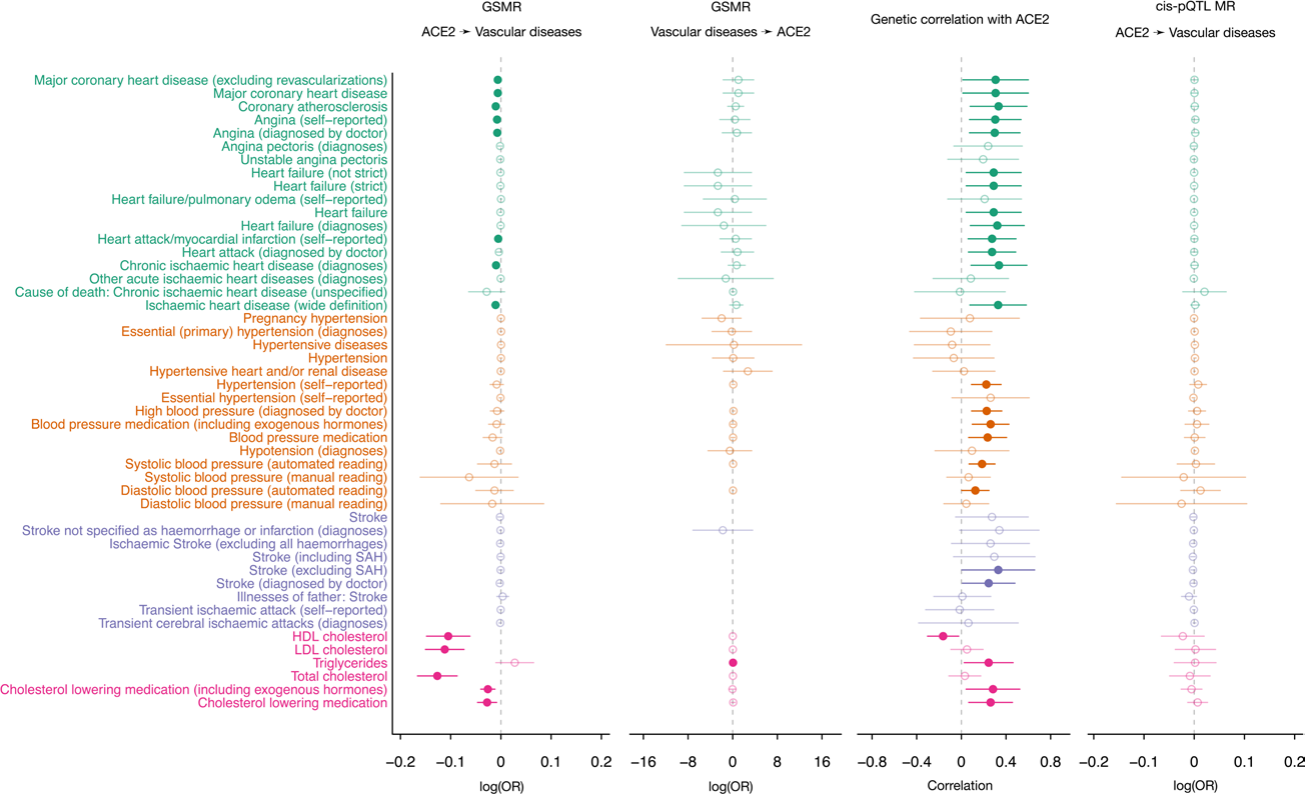
**Genetic and causal relationships between plasma ACE2 and vascular diseases.** Estimates significantly different from zero are highlighted in filled circles. First 2 forest plots show the bidirectional generalized summary-level mendelian randomization (GSMR) analysis results between plasma ACE2 (angiotensin-converting enzyme 2) and 48 vascular disease–related traits. Third forest plot gives the corresponding genetic correlations estimates between plasma ACE2 and these phenotypes. Last forest plot shows the estimated mendelian randomization (MR) effects based on cis–protein quantitative trait loci (pQTL) only. HDL indicates high-density lipoprotein; LDL, low-density lipoprotein; OR, odds ratio; and SAH, subarachnoid hemorrhage.

Next, to examine the shared genetic basis between plasma ACE2 level and specific vascular diseases or diagnoses, we specifically extracted the GWAS summary-level data for 48 vascular disease–related phenotypes from UK Biobank (see Methods and Table S10), categorized into 18 for heart disease, 15 for blood pressure, 9 for stroke, and the other 6 for blood lipids. We assessed genetic correlations and found that plasma ACE2 levels had positive genetic correlations with most of the vascular disease phenotypes, except for high-density lipoprotein cholesterol (Figure 3 and Table S11).

Next, we conducted bidirectional causal inference using GSMR to identify potential causal effects of ACE2 on these traits (Figure 3 and Table S12). In the GSMR analysis, the instrumental variables used to assess causality are restricted to the autosomal trans-pQTL for ACE2. Paradoxically, GSMR suggested that genetically elevated plasma ACE2 level is associated with reduced risk of heart disease, high-density lipoprotein, low-density lipoprotein, and total cholesterol. However, the results of MR analyses can be biased if some of the genetic instruments are invalid because they additionally influence the outcome through pathways other than ACE2 (so-called horizontal pleiotropy).

Because several of the ACE2-associated autosomal loci are pleiotropically associated with various cardiometabolic and inflammation-related phenotypes, we further conducted the MR analysis for vascular diseases using only the X chromosome cis-pQTL *ACE2* locus. No significant causal effect was detected (Figure 3 and Table S13) for any of the phenotypes with summary association statistics of the X chromosome. It should be noted that although on one hand using only cis-pQTL as the MR instrument may avoid the issue of bias owing to horizontal pleiotropy, on the other hand, there may be reduced power to detect a true causal effect using only the cis-pQTL because of the smaller number of SNPs considered.

### Genetic Correlation and Causal Inference Between ACE2 and COVID-19

**Figure 4.**
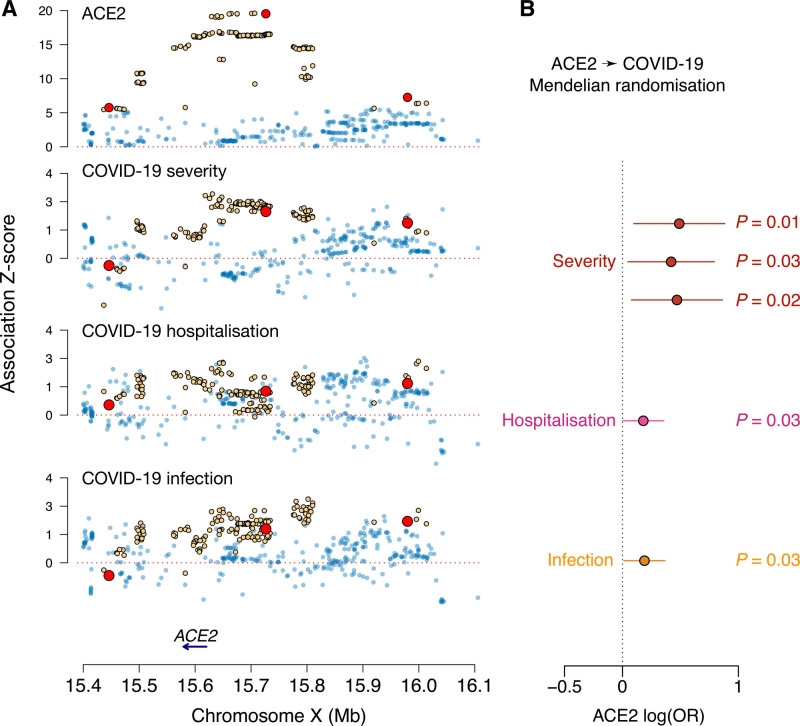
**Causal inference between plasma ACE2 and COVID-19 based on the *ACE2* cis-pQTL. A**, The regional genome-wide association study *z* scores across 4 traits are compared; alleles are coded so that the estimated single nucleotide polymorphism (SNP) effects on ACE2 (angiotensin-converting enzyme 2) are all positive. Genome-wide significant SNPs for ACE2 (*P*<5×10^−8^) are highlighted in yellow. The 3 SNPs representing independent significant associations after linkage disequilibrium (LD) clumping (*r*^2^<0.001) are marked in red. **B**, Inference of the causal effect of ACE2 on COVID-19 through mendelian randomization (MR). The MR was performed with an inverse-variance weighted causal effect estimator based on multiple genome-wide significant cis-regulatory SNPs. A threshold of *R*^2^<0.001 was applied to prune out SNPs in LD. Whiskers represent 95% CIs. OR indicates odds ratio.

Using the latest genome-wide association summary statistics for severe COVID-19,^20^ we obtained an estimated autosomal genetic correlation of 0.476 (*P*=9.4×10^−3^) with plasma ACE2, indicating an increased risk of severe COVID-19 for people who have genetically raised levels of circulating ACE2 protein. At the cis-pQTL located near the *ACE2* gene, the lead variant rs4830984 was nominally significantly associated with severe COVID-19 (*P*=0.026).^20^ GSMR analysis based on all the discovered ACE2 pQTL as instruments revealed no significant causal effect. To avoid horizontal pleiotropy, we considered in our MR analysis only the independent cis variants as genetic instruments for causal inference (Figure 4A). Using inverse variance–weighted MR, we estimated an odds ratio of 1.63 (95% CI, 1.10–2.42; *P*=0.01) for ACE2 on COVID-19 severity (Figure 4B). Reverse MR analysis instrumenting on the severe COVID-19 loci did not reveal a significant estimate (GSMR *P*=0.95), suggesting the absence of a causal effect of COVID-19 on ACE2 levels. As validation, in the Host Genetics Initiative COVID-19 hospitalization data (GWAS B2, GenOMICC subtracted), we estimated the causal effect of ACE2 with an odds ratio of 1.52 (95% CI, 1.05–2.21; *P*=0.03), and in the Host Genetics Initiative COVID-19 infection data (GWAS C2, GenOMICC subtracted), the estimated odds ratio was 1.60 (95% CI, 1.08–2.37; *P*=0.02).

## Discussion

We provide a detailed characterization of the phenotypic and genetic correlates of plasma ACE2 using data from several large cohort studies, including the identification of 8 novel genetic loci, which together explain 30% of the heritability of the protein. We further demonstrate that ACE2 levels are genetically correlated with both CVD and COVID-19 clinical outcomes. Moreover, we found genetic evidence that elevated ACE2 levels may be causally related to COVID-19 severity, hospitalization, and infection. These data add further evidence that the cardiovascular complications of patients with COVID-19 may be mechanistically related to ACE2.

### Genetics of Plasma ACE2 Levels

Previous studies have measured the heritability of ACE2 levels,^44,45^ with the SNP heritability estimated to be 33% in the PURE study (Prospective Urban Rural Epidemiology)^45^ using the same Olink PEA-based ACE2 antigen assay. The heritability was estimated to be 16% among Europeans (with somewhat higher estimates for Latinos and Persians) in our study. Differences in heritability estimation methods or geographic or demographic differences between populations may account for different heritabilities across populations. Despite these differences, we were able to confirm 2 recently identified genetic loci associated with ACE2, the cis-pQTL on chromosome X and *HNF1A*. The peak signal on the X chromosome is located ≈100 kb upstream of *ACE2* and therefore likely reflects regulatory effects on *ACE2* expression. These variants are not in LD with several common missense variants of *ACE2* that are predicted to affect ACE2 protein stability^46^ or SARS-CoV-2 binding.^47,48^ The presence of a strong X-linked locus may explain in part the observed sex differences in *ACE2* expression across various tissues^49^ and the higher circulating levels of ACE2 in men.

Along with the *HNF1A* ACE2-associated autosomal locus, the 8 newly identified genetic loci associated with ACE2 may help to shed additional light on mechanisms by which cellular or plasma ACE2 levels are regulated under physiological and pathological conditions.^49^ The transcription factors HNF1A and HNF4A regulate *ACE2* expression in the pancreas and gastrointestinal tract.^29,30^ Besides *ACE2* expression itself, we identified another discovered novel locus being the transcription factor binding site of HNF4A. The *HNF1A*, *HNF4A*, and *TRIB1* loci also are important determinants of CVD, diabetes, lipids, and adiposity-related traits and therefore likely contribute to the genetic correlations we observed between these cardiometabolic phenotypes and the findings from MR analysis between ACE2 for BMI and diabetes in the PURE study. Although the *ACE2* locus on chromosome X and several of the newly identified autosomal genetic loci likely influence ACE2 levels through regulation of cellular expression or transcription of *ACE2* in various tissues, other loci may influence the amount of ACE2 shedding from the cell membrane. In this regard, the protease inhibitor α-1-antitrypsin is capable of inhibiting the enzymatic activities of 2 proteases, TMPRSS2 and ADAM17, both of which are involved in ACE2 shedding.^38,39^

### Relationship of ACE2 and Renin-Angiotensin System to CVD

In our analysis, soluble ACE2 levels were positively correlated with several traditional CVD risk factors, which is consistent with its important role in counterregulation of the renin-angiotensin system. Higher ACE2 has additionally been associated with poorer prognosis in patients with preexisting CVD and recently was found to prospectively predict incident CVD, mortality, diabetes, and heart failure in previously unaffected individuals,^9–12^ independently of traditional risk factors.^45^ In particular, ACE2 was ranked higher as a predictor of overall mortality compared with smoking, diabetes, blood pressure, BMI, and lipids. We also found that higher plasma ACE2 level is genetically correlated with a higher risk of vascular diseases, including coronary heart disease, hypertension, stroke, and heart failure. Nonetheless, MR analysis restricted to the cis-pQTL ACE2 locus was unable to demonstrate a causal relationship between ACE2 and CVD-related outcomes. We speculate that the apparent but paradoxical protective effect of ACE2 on CVD by GSMR using the additional autosomal trans-pQTL associations may reflect that extensive pleiotropy exhibited by most of the autosomal trans-pQTL on blood lipids and other CVD risk factors.

Both ACE and ACE2 have connections with cardiovascular mechanisms. Although the sparse genetic architecture of both proteins did not result in significant genetic correlation, a suggestive causal effect of ACE2 on ACE was detected (see also Supplemental Material). We have shown that ACE2 is genetically correlated with a series of vascular traits, whereas its causal role was detected only for CVD-related phenotypes; in contrast, the hypertension target ACE showed a clear causal effect on blood pressure. Our results thus suggest distinct downstream functions of the 2 homologous proteins.

### Relationship of ACE2 to COVID-19

In addition to its role in the regulation of the renin-angiotensin system, cellular ACE2 is an important receptor for SARS-CoV-2 and other coronaviruses. Our findings of a genetic correlation of soluble ACE2 and additional evidence that genetically determined soluble ACE2 is associated with increased risk of COVID-19 severity are consistent with recent in vitro data that the secreted form of ACE2 plays a direct role in cell entry of SARS-CoV-2 through receptor-mediated endocytosis.^50^ Our results are also consistent with human genetic studies indicating that genetic variation in soluble ACE2 influences COVID-19 risk.^42,51^

According to animal studies, ACE inhibitors or ARBs may upregulate *ACE2* gene expression in cardiac cells,^52,53^ which might increase COVID-19 susceptibility. However, human observational studies have not found a robust relationship between higher ACE2 levels and ACE inhibitor or ARB use.^13,14^ Consistent with these results, we did not find a significant association of ACE inhibitors or ARBs with plasma ACE2.

### Study Limitations

Although our study adds important information on the regulation of ACE2 and the genetic relationship between ACE2 and other phenotypes, several limitations should be highlighted.

(1) The genetic loci identified herein are associated with the soluble form of ACE2 found in human plasma, which is relatively easily obtainable and can be studied with sufficiently large sample sizes. The relationship of the plasma ACE2 pQTL or other genetic loci with tissue levels of the full-length cellular ACE2 receptor, the precise genetic regulatory mechanisms underlying these associations, and the relevant tissue sources of soluble ACE2 remain to be determined. (2) Despite the large sample size, we were able to account for only 30% of the heritability of ACE2, leaving the remaining loci (eg, common genetic variants with smaller effect sizes or rare genetic variants of large effect) to be discovered. (3) The Olink PEA assay quantifies plasma ACE2 concentration rather than the enzymatic activity of the protein. The activity of ACE2 as an enzyme may or may not have the same biological basis as its general abundance. An earlier family-based study of plasma ACE2 activity levels in healthy individuals^44^ estimated that genetic factors accounted for 67% of the phenotypic variance in ACE2, but only 7% of individuals had detectable ACE2 levels with a fluorogenic activity assay. With the use of a more sensitive fluorogenic assay, readouts from an ACE2 enzyme activity assay tend to have a strong correlation with protein levels of ACE2.^54^ Given the specificity of the Olink ACE2 assay (Supplemental Material), one can expect that the effects on Olink and enzyme activity assay measurements would likely be directionally concordant.

### Conclusions

Our findings suggest that ACE2 may play an important role not only in susceptibility to cardiovascular, metabolic, and pulmonary disorders but also in susceptibility to COVID-19 severity. These findings have potential therapeutic implications for the counterregulatory ACE2/angiotensin-(1–7) axis in modulating COVID-19 severity.^55^ In particular, identification of additional genetic factors involved in the regulation of ACE2 levels may help disentangle the potential causal roles of soluble versus cellular ACE2 in the regulation of chronic diseases, COVID-19 infection, disease severity, and immunity.

## Article Information

### Acknowledgments

The authors thank Dr Jie Zheng at the University of Bristol for sharing the harmonized GWAS summary statistics used in LD-Hub and IEU Open GWAS Project. The authors also thank Marie Pigeyre and Guillaume Paré at McMaster University for sharing the serum ACE GWAS summary statistics from the ORIGIN trial. They thank Yuanyuan Sun and Ao Lan at Sun Yat-sen University for useful comments on the manuscript. They thank the Edinburgh Compute and Data Facility at the University of Edinburgh for providing high-performance computing resources. They thank the members of the cited consortia of GWAS for making their data available. Cohort-specific acknowledgments are given in the Supplemental Material. X.S. and J.F.W. initiated and coordinated the study. E.M.-D. and P.K.J. contributed to autosomal meta-analysis. Z.Y., X.S., A. Richmond, and L.K. contributed to X chromosome meta-analysis. Z.Y., N.P., T.H., Y.H., and H.G. contributed to the MR analysis. Z.Y., J.C., R.Z., T.L., Y.W., and K.Y. contributed to functional analysis. Z.Y., Z.N., and C.Z. contributed to heritability and genetic correlation analysis. S.G., B.P., A. Ramisch, E.T.D., G.P., N.E., J.H., X.H., D.Z., T.B., S.-J.H., E.W., M.P., L.M.R., A.K., J. Peters, A.V., L.L., S. Elmståhl, G.D., J. Petrie, O.P., L.F. Y.C., C.Y., U.V., T.E., S. Enroth, Å.J., U.G., C.L., D.L., C.H., T.L.A., C.K., A.W.M., A.S., L.W., A.G., E.Z., J.M.S., A.B., K.M., and A.M. contributed to the cohort-level analysis. L.R., E.P.-C., S.C., A.D.B., K.R., and J.K.B. contributed to GenOMICC severe COVID-19 genomic analysis. X.S. drafted the manuscript. L.K., Y.P., A.P.R., and J.F.W. contributed to manuscript writing. All authors gave final approval to publish.

### Sources of Funding

Dr Shen received a Swedish Research Council Starting Grant (No. 2017-02543) and National Natural Science Foundation of China grant (No. 12171495). Drs Wilson and Hayward acknowledge support from the Medical Research Council Human Genetics Unit program grant “Quantitative Traits in Health and Disease” (U. MC_UU_00007/10). The work of Dr Klaric was supported by an RCUK Innovation Fellowship from the National Productivity Investment Fund (MR/R026408/1). Drs Zanetti, Petrie, and Assimes were supported by grant R01DK114183. Dr Bretherick acknowledges funding from the Wellcome PhD training fellowship for clinicians (204979/Z/16/Z), the Edinburgh Clinical Academic Track program. Dr Reiner was supported by grants R01HL146500 and R01HL132947.

### Disclosures

Dr Bretherick has received grants outside of this work from AstraZeneca, Bayer, Biogen, Bioverativ, Merck, Novartis, Pfizer, and Sanofi. Dr Dermitzakis is currently an employee of GSK, and the work was performed before he joined GSK. The other authors report no conflicts.

### Supplemental Material

Methods

Results and Discussion

Figures S1–S24

Tables S1–S21

References 56–58

## Supplementary Material
