# Supplementary material for: Genetic Landscape of the ACE2 Coronavirus Receptor
Source: Circulation. 2022 Apr 7;145(18):1398–411. doi: 10.1161/CIRCULATIONAHA.121.057888 (PMC9047645; doi:10.1161/CIRCULATIONAHA.121.057888)
Supplement: Supplementary file 1 [file cir-145-1398-s001.pdf]

SUPPLEMENTAL MATERIAL for

Genetic landscape of the ACE2 coronavirus receptor

Yang et al.

Contents

1 SUPPLEMENTAL METHODS **2**

2 SUPPLEMENTAL RESULTS & DISCUSSION **10**

3 SUPPLEMENTAL FIGURES **13**

4 SUPPLEMENTAL TABLES **37**

5 SUPPLEMENTAL INFORMATION **40**

1 SUPPLEMENTAL METHODS

Cohorts

NSPHS The Northern Sweden Population Health Study (NSPHS) represents a cross-sectional study conducted in the communities of Karesuando (samples gathered in 2006) and Soppero (2009) in the subarctic region of the County of Norrbotten, Sweden. NSPHS consists of 1069 individuals that were 14 to 94 years of age at time of sample collection. The NSPHS was approved by the local ethics committee at the University of Uppsala (Regionala Etikprövningsnämnden, Uppsala, 2005:325, and extension of the project was approved 2016-03-09). Informed consent to the study was given by all participants, including the examination of environmental and genetic cause of disease. If a person was not of age (<18 years), a legal guardian signed additionally.

ORCADES The Orkney Complex Disease Study (ORCADES) is a family-based, cross-sectional study that seeks to identify genetic factors influencing cardiovascular and other disease risk in the isolated archipelago of the Orkney Isles in northern Scotland (McQuillan et al., 2008). Genetic diversity in this population is decreased compared to Mainland Scotland, consistent with the high levels of endogamy historically. 2078 participants aged 16-100 years were recruited between 2005 and 2011, most having three or four grandparents from Orkney, the remainder with two Orcadian grandparents. Fasting blood samples were collected and many health-related phenotypes and environmental exposures were measured in each individual. All participants gave written informed consent and the study was approved by Research Ethics Committees in Orkney and Aberdeen (North of Scotland REC).

FENLAND The Fenland study is a population-based cohort of 12,435 participants of Caucasian-ancestry born between 1950 and 1975 who underwent detailed phenotyping at the baseline visit from 2005 to 2015. Participants were recruited from general practice surgeries in the Cambridgeshire region in the UK. Exclusion criteria were: clinically diagnosed diabetes mellitus, inability to walk unaided, terminal illness, clinically diagnosed psychotic disorder, pregnancy, or lactation.

HELIC MANOLIS The HELIC (Hellenic Isolated Cohorts; www.helic.org) MANOLIS (Minoan Isolates) collection focuses on Anogia and surrounding Mylopotamos villages. Recruitment of this population-based sample was primarily carried out at the village medical centres. All individuals were older than 17 years and had to have at least one parent from the Mylopotamos area. The study includes biological sample collection for DNA extraction and lab-based blood measurements, and interview-based questionnaire filling. The phenotypes collected include anthropometric and biometric measurements, clinical evaluation data, biochemical and haematological profiles, self-reported medical history, demographic, socioeconomic and lifestyle information. The study was approved by the Harokopio University Bioethics Committee and informed consent was obtained from every participant.

HELIC POMAK The HELIC (Hellenic Isolated Cohorts; www.helic.org) Pomak collection focuses on the Pomak villages, a set of isolated mountainous villages in the North of Greece. Recruitment of this population-based sample was primarily carried out at the village medical centres. The study includes biological sample collection for DNA extraction and lab-based blood measurements, and interview- based questionnaire filling. The phenotypes collected include anthropometric and biometric measurements, clinical evaluation data, biochemical and haematological profiles, self-reported medical history, demographic, socioeconomic and lifestyle information. The study was approved by the Harokopio University Bioethics Committee and informed consent was obtained from every participant.

ARISTOTLE The ARISTOTLE trial included 18 201 patients with AF and an increased risk of stroke who were randomized to a median of 1.9 years treatment with warfarin or apixaban. Biomarker samples at baseline were available from 14 611 participants and a random sample of 3999 patients was used for the multiplex proteomics analysis including the sACE2 measurements in the current study. The trial complied with the Declaration of Helsinki and were approved by appropriate ethics committees at all sites. All patients provided written informed consent. A subset of the patients with available sACE2 measurements participated in the ARISTOTLE genetic substudy. Participation in the ARISTOTLE genetic substudy with whole blood sampling for DNA extraction was voluntary and based on signing a separate consent form.

DIRECT The DIRECT (Diabetes Research on Patient Stratification) consortium includes pre-diabetic participants (target sample size 2,200–2,700) and patients with newly diagnosed type 2 diabetes (target sample size 1,000) pro existing cohort studies across Europe: Metabolic Syndrome in Men (METSIM) (Finland); Relationship between Insulin Sensitivity and Cardiovascular disease (RISC), Hoorn Meal Study (HMS), and New Hoorn Study (NHS) (Netherlands); Health2010, Health2006, Danish Study of Functional Disorders (DanFunD) (Dandund-web), and Gut, Grain and Greens (GGG) studies (Denmark); Malmö Diet and Cancer (MDC) study (Sweden). Blood samples from venous blood were collected together with other biochemical analyses. All measurement procedures are standardized across study sites and performed by trained nurses or research assistants. Height is measured using calibrated wall-mounted stadiometers, weight using calibrated scales, and waist, hip, thigh and calf circumferences using non-stretchable measuring tapes (Koivula et al. 2019).

Approval for the study protocol was obtained from each of the regional research ethics review boards separately (Lund, Sweden: 20130312105459927, Copenhagen, Denmark: H-1-2012-166 and H-1-2012-100, Amsterdam, Netherlands: NL40099.029.12, Newcastle, Dundee and Exeter, UK: 12/NE/0132), and all participants provided written informed consent at enrolment. The research conformed to the ethical principles for medical research involving human participants outlined in the Declaration of Helsinki.

EGCUT The Estonian Biobank cohort is a volunteer-based sample of the Estonian resident adult population (aged $\leqslant$ 18 years). All participants have signed a broad informed consent form, which allows the continuous periodical

linking to national electronic databases and recontacting of participants (Leitsalu et al., 2015). The cohort is currently composed of over 200,000 participants, which is around 20% of the Estonian adult population and the ratio of males and females within biobank reflects the general population of Estonia. All of the gene donors have genotyping data available. In this project the subsample of 500 individuals with available proteomics data were used. The subcohort contains 52.8% females and 47.2% males with the mean age of 53.932 (s.d. =14.002).

VIS The CROATIA_Vis study includes 1008 Croatians, aged 18–93 years, who were recruited from the villages of Vis and Komiza on the Dalmatian island of Vis during 2003 and 2004 All participants were volunteers and gave informed consent. They underwent a medical examination and interview, led by research teams from the Institute for Anthropological Research and the Andrija Stampar School of Public Health, (Zagreb, Croatia). All subjects visited the clinical research centre in the region, where they were examined in person and where fasting blood was drawn and stored for future analyses. Many biochemical and physiological measurements were performed, and questionnaires of medical history as well as lifestyle and environmental exposures were collected. The study received approval from the relevant ethics committees in Scotland and Croatia and complied with the tenets of the Declaration of Helsinki.

WHI The Women's Health Initiative (WHI) recruited 161,808 post-menopausal women age 50-79 years between 1993 and 1998 (baseline) at 40 clinical centers in the United States. Further details of the WHI design, recruitment and enrollment, and study population have been described in ref. 55. A broad array of demographic, clinical, and lifestyle characteristics were collected from each participant in the WHI through questionnaires, clinical examinations, periodic follow-up, and medical records, Plasma ACE2 levels were measured in a subset of WHI participants (using the Olink CVDII panel) who were enrolled in WHI Long Life Study and had blood specimens collected between March 2012 and May 2013 and who also underwent whole genome sequencing (>38x coverage) through the NHLBI Trans-Omics for Precision Medicine (TOPMed) program. All participants gave written informed consent and the study was approved by Human Subjects Committee at the Fred Hutchinson Cancer Research Center (Seattle, Washington, USA).

Quality control of each cohort

NSPHS Samples were sequenced using Illumina short read technology (X-ten) to 30x coverage per individual. WGS data was aligned to the GR37 using bwa-mem v0.7.1243. The raw alignments were then processed according to GATK best practice using GATK v3.3. Variants were called by the GATK HaplotyeCaller 3.3 followed by variant quality score recalibration (VQSR). Only, biallelic SNPs and indels that did not deviate from Hardy-Weinberg equilibrium ($P > 1{\times}10^{-8}$) were included. We also filtered on genotype quality (GQ) > 50, and SNVs and indels with >10% missing genotypes and MAF< 0.0017 (less than 3 observations in the cohort) were removed. No cohort-specific variables were used.

FENLAND Fenland study participants were genotyped using the Affymetrix UK Biobank Axiom array. Samples were excluded for the following reasons: (1) failed channel contrast (DishQC < 0.82), (2) low call rate ($< 95\%$), (3) gender mismatch between reported and genetic sex, (4) heterozygosity outlier, (5) unusually high number of singleton genotypes or (6) impossible identity-by-descent values. Single nucleotide polymorphisms (SNPs) were removed if: (1) call rate $< 95\%$, (2) clusters failed Affymetrix SNPolisher standard tests and thresholds, (3) MAF was significantly affected by plate, (4) SNP was a duplicate based on chromosome, position and alleles (selecting the best probeset according to Affymetrix SNPolisher), (5) Hardy-Weinberg equilibrium $P < 10^{-6}$, (6) did not match the reference, or (7) MAF$= 0$. Ancestry outliers and related individuals were also excluded.

Autosomes were imputed to the HRC (r1) panel using IMPUTE4, and the X-chromosome was imputed to HRC.r1.1 using the Sanger imputation server. Imputation to the HRC panel was supplemented with any additional variants in the UK10K+1000Gphase3 reference panel, imputed using the Sanger imputation server. Variants with MAF< 0.001, imputation quality (info) < 0.4, or Hardy-Weinberg equilibrium $P < 10^{-7}$ were excluded from further analyses.

Protein levels (on the log2 scale) were transformed using the rank-based inverse normal transformation, then adjusted for age and 10 genetic principal components. Genetic association under an additive model was performed using BGENIE (v1.3).

HELIC MANOLIS Basecall files for each sequencing lane were transformed into unmapped BAMs using Illumina2BAM, marking adaptor contamination and decoding barcodes for removal into BAM tags. PhiX control reads were mapped using BWA Backtrack and were used to remove spatial artefacts. Reads were converted to FASTQ and aligned using BWA MEM 0.7.8 to the hg38 (GRCh38) with decoys (HS38DH) reference. The alignment was then merged into the master sample BAM file using Illumina2BAM MergeAlign. PCR and optical duplicates are marked using biobambam markduplicates and the files were archived in CRAM format. Per-lane CRAMs were retrieved and reads pooled on a per-sample basis across all lanes to produce library CRAMs; these were each divided in 200 chunks for parallelism. GVCFs were generated using HaplotypeCaller v.3.5 from the Genome Analysis Toolkit (GATK) for each chunk. Ploidy was set to 1 for non-pseudoautosomal sex chromosome regions. All chunks were then merged at sample level, samples were then further combined in batches of 150 samples using GATK CombineGVCFs v.3.5. Variant calling was then performed on each batch using GATK GenotypeGVCFs v.3.5. The resulting variant callsets were then merged across all batches into a cohort-wide VCF file using bcftools concat. Variant-based quality control was performed for autosomes only using GATK Variant Quality Score Recalibrator. Sample quality control was aided by comparison to genotyping array data in the same samples.

In MANOLIS, four individuals failed sex checks, 8 samples had low concordance ($\hat{\pi} < 0.8$) with chip data, 11 samples were duplicates, and 12 samples displayed traces of contamination (Freemix score from the verifyBamID suite$> 5\%$). In case of sample duplicates, the sample with highest quality metrics (depth, freemix and chipmix score) was kept. As contamination and sex mismatches were correlated, a total of 25 individuals were excluded ($n = 1,457$).

No further samples were excluded based on depth, heterozygosity, transition/transversion (Ti/Tv) rate, missingness or ethnicity.

HELIC POMAK 1,642 Pomak samples underwent the same sequencing, variant calling, variant QC and sample QC procedure as MANOLIS. As part of sample QC, three duplicates were removed, as well as 9 sex check failures, 11 contamination outliers, as well as one missingness and one average depth outlier (final $n = 1,617$).

ARISTOTLE The genotyping was performed using the Illumina Global Screening Array (24v2). Quality controls were performed using the whole-genome association analysis toolset PLINK v1.9 (https://www.cog-genomics.org/plink/) which included sex check, call rate per SNP and individual > 98%, MAF > 0.001 and HWE $P > 10^{-8}$. Imputation of genotypes was performed using the Sanger imputation service with the Haplotype reference consortium v1.1 as reference, Eagle v2.3.3 for phasing and PBWT for imputation. No cohort specific covariates were used.

DIRECT Blood collection and DNA extractions was carried out using Maxwell 16 Blood DNA purification kits and a Maxwell 16 semi-automated nucleic acid purification system Page 16 of 56 (Promega). Genotyping was conducted using the Illumina HumanCore array (HCE24 v1.0) and genotypes were called using Illumina's GenCall algorithm. Samples were excluded for any of the following reasons: call rate < 97%; low or excess mean heterozygosity; sex discordance; and monozygosity. Genotyping quality control was then performed to provide high-quality genotype data for downstream analyses using the following criteria: call rate < 99%; deviation from Hardy-Weinberg equilibrium (exact $P < 0.001$); variants not mapped to human genome build GRCh37; and variants with duplicate chromosome positions (a total of 30,318 markers were excluded).

Sex of the genotype samples was assessed using the mean homozygosity rate across the X-chromosome calculated using the –check-sex command in PLINK (Purcell et al. 2007). For 91 samples (including 22 with unspecified sex and 10 samples with undetermined genotypic sex) the sex discrepancy between genotyping data and reported sex could not be resolved. To identify possible ethnical outliers in the DIRECT data, we performed a principal component analysis (PCA) using the genotype data from our studied population (3,102 samples; 547,644 markers) using the following cut-offs MAF> 0.01, HWE $P > 10^{-4}$ and call rate> 90% (280,845 markers and 3,033 samples in the analysis after excluding 69 samples with low call rate and duplicate sample). The clean DIRECT data (all samples which had previously failed QC are excluded in this step, missing sex samples were retained) was merged with data from the publicly available 1,000 Genomes project database, and thinned down to only contain markers that were present in both dataset and not palindromic (241,092 markers). This PCA was performed with GCTA software (command –pca). For these 4,127 samples (3,033 from DIRECT and 1,094 from 1,000 Genomes), ethnic characterization and genotype calls at the 241,092 SNPs were available. The first factorial plane was sufficient to discriminate ethnic origin (more accurately, the recruitment centers in DIRECT cohort). A total of 3,033 samples and 517,958 markers across the two studies passed quality control procedures. Imputation to the 1000 Genomes Phase 3 CEU reference panel was

performed with ShapeIt (v2.r790) and Impute2 (v2.3.2).

ACE2 protein abundance were measured using the Olink® Cardiovascular II (Olink Proteomics AB, Uppsala, Sweden) according to the manufacturer's instructions. The obtained data was processed using Olink's NPX manager software version 0.0.85.0. The internal controls are designed to mimic and monitor the different steps of the PEA. They consist of two incubation/immuno controls, an extension control and a detection control. The internal controls are introduced to all samples as well as to the external controls and are used for quality control and normalization of the data. The external controls consist of a negative control used to calculate the limit of detection (LOD), as well as a triplicate of inter-plate controls (IPCs) that are used for data normalization. Quality control of the data is performed in two steps: First, the run is quality controlled by calculating the standard deviation for the detection control and the incubation/immuno controls. The standard deviation should be below 0.2 for a run to pass quality control. Secondly, each sample is quality controlled by comparing the results for the detection control and one of the incubation controls against the run median. Samples that fall more than 0.3 NPX from the run median with regards to these two internal controls will fail the quality control. Assays on individual plates influencing the LOD determination highly, due to an overall lower or higher signal, were excluded as well as whole plates, with more than 50% of all assays excluded due to the previous reasoning. All assay validation data (detection limits, intra- and inter-assay precision data, etc.) are available on manufacturer's website (www.olink.com).

We performed association tests of imputed SNPs on the autosomal chromosomes with the SNPTEST program (version 2.5.2). We used the options '-frequentist 1' to perform a frequentist additive test, and '-method score' to apply a missing data likelihood score test. Moreover, we included following covariates into the testing model: type 2 diabetes status (diabetic or pre-diabetic), center, sex, age, plate, as well as 10 genotype PCs.

EGCUT Genotyping was performed using Illumina HumanOmniExpress arrays and Illumina Genomestudio was used for genotype calling (Illumina Inc., San Diego, US). Samples were excluded based on call rate of $\geqslant 95\%$. Additional samples were excluded based on gender mismatch, ethnic outliers, heterozygosity > 3 s.d. from the mean and cryptic relatedness. Pre-imputation SNP quality control was done using minor allele frequency cutoff of $\geqslant 1\%$, on the basis of call rate ($\leqslant 98\%$) across samples and deviation from Hardy-Weinberg equilibrium ($\leqslant 10^{-6}$). Genotypes were phased by using Eagle 2.3 and imputed to the Estonian-specific reference panel by Beagle 2.4.2 with default parameters.

Plasma concentrations of ACE2 were measured in EDTA plasma samples using the ProSeek Cardiovascular II panel (Olink Biosciences, Uppsala, Sweden), according to the standard protocol. Prior to statistical analyses, samples that didn't pass Olink internal quality control were removed. Rank-based inverse normal transformation was performed on raw ACE2 measurements and then linear regression with adjustments was conducted. Proteomics data was corrected for possible population stratification by adjusting for the first 4 MDS that were calculated from genotype data using PLINK 2.0 software, additional covariates were age, sex, Olink batches, Olink sample position, season of sample collection, sample preparation time until storage in days, sample storage time in days. The standardised residuals

were used in a linear regression-based association analysis using "–dosage" flag in PLINK 1.9 software to test association between genetic data and ACE2 levels.

VIS The CROATIA_Vis samples were genotyped using the Illumina's Sentrix HumanHap300 Genotyping BeadChip (v1).We excluded individuals with a genotyping rate lower than 95%. and SNPs showing departure from Hardy-Weinberg equilibrium ($P < 10^{-6}$) , call rate < 95% and MAF< 0.01.

Conditional analysis

We performed the multi-SNP conditional analysis using the COJO module[26] in the GCTA software. For the input GWAS summary statistics, we used our genome-wide association meta-analysis summary-level data and the 1000 Genomes Phase 3 data as the reference panel for LD. SNPs with minor allele frequency (MAF) less than 0.01 were filtered out. The genome-wide significance threshold of p-values was set to 5×10^{-8}. The collinearity cutoff for LD r^2 was set to 0.9.

Analysis of ACE2 pQTL associations with other complex traits

We investigated the gene-based associations for the pQTL of plasma ACE2 on other complex traits based on the GWAS catalog database, using the GENE2FUNC module of FUMA[56]. Phenotypes that have a significant enrichment of ACE2 pQTL mapped genes were reported (false discovery rate < 0.05). The mapped genes were based on the SNP2GENE module of FUMA, underlying genome-wide significant SNPs for plasma ACE2.

Analysis of ACE2 cis-eQTL effects on UK Biobank phenotypes

We extracted the cis-eQTL results for ACE2 gene expression from the GTEx project portal for 44 human tissues. The 1 Mb cis-window harboured 3,867 SNPs. We also extracted the Neale's lab UK Biobank GWAS summary statistics for the X chromosome across 3,990 phenotypes. In order to represent the cis-eQTL across multiple tissues and link to UK Biobank GWAS results, we investigated the common SNP (minor allele frequency (MAF) > 0.05) upstream of ACE2 that is closest to the transcription starting site (TSS), X:15618063 (Ch37 reference, MAF = 0.4572, distance to TSS = -2208 bp). At a p-value threshold of 0.05/3867, we detected the significant eQTL represented by this SNP and performed Mendelian randomization analysis across all the UK Biobank phenotypes. The p-value distribution across the UK Biobank phenotypes were evaluated.

Data availability

COVID-19 Host Genetics Initiative (HGI) summary statistics: https://www.covid19hg.org/; UK Biobank GWAS summary statistics: http://www.nealelab.is/uk-biobank; GTEx eQTL summary statistics in human tissues: https://gtexportal.org/home/datasets; eQTLGen summary-level data: https://www.eqtlgen.org/cis-eqtls.html; LD-Hub: http://ldsc.broadinstitute.org/lookup/; Consolidated epigenomes (Roadmap + ENCODE): https://egg2.wustl.edu/roadmap/web_portal/meta.html; IEU Open GWAS Project: https://gwas.mrcieu.ac.uk/.

Code availability

HDL:https://github.com/zhenin/HDL; LDSC: https://github.com/bulik/ldsc; GCTA: https://cnsgenomics.com/software/gcta.

2 SUPPLEMENTAL RESULTS & DISCUSSION

Specificity and accuracy of the Olink ACE2 assay

The specificity of the Olink ACE2 assay was tested by running the assays against 96 antigens (**Table S14**), including some with strong homology to ACE2 such as ACE. The antigens were tested at both 50 and 5 ng-ml. The experiments were run two times at both concentrations. A signal at LOD+1 NPX was considered unspecific. ACE2 did not yield an unspecific signal in any of the experiments.

Moreover, the antibodies used have been tested by the producer of the antibodies, who reported less than 1% cross-reactivity against ACE. The antibodies are polyclonal and were developed by immunization against Mouse myeloma cell line NS0-derived recombinant human ACE-2 Gln18-Ser740. The antibodies are commercially available but the exact article is Olink proprietary information.

Besides, the discovered strong cis-pQTL and trans-pQTL (e.g., the transcription factor HNF1A) for ACE2 already provide justification of the specificity of the Olink assay: 1) Without specific measurement of the protein, we would not be able to map the cis-pQTL on the X chromosome with great statistical significance; 2) It is unlikely that our pQTL discoveries were driven by an epitope effect (where one amino-acid variant is preferentially bound by the antibodies) – the trans-pQTL harbor biological relevant candidates such as HNF1A and HNF4A, and the HNF1A association colocalizes with the cis-eQTL association of HNF1A (**Figure S21**) – these would be unlikely to be detected if the ACE2 cis-pQTL was due to an epitope effect of the assay.

Genetic relationships between ACE2 and ACE

GTEx gene expression data across different human tissues revealed significant positive correlations between *ACE* and *ACE2* in blood vessel, colon, and small intestine, whereas negative correlations were found in many other tissues including adipose tissue, breast, heart, lung, and so on (**Figure S22**, **Table S15**). With our large-scale genomic analysis on ACE2, we investigated the genetic relationship between ACE and ACE2. Due to the relatively sparse genetic architecture of the proteins, insignificant positive genetic correlations were detected (**Figure S23A**). Nevertheless, bidirectional Mendelian randomization analysis suggested a weak causal effect of ACE2 on ACE, i.e., the cis-pQTL allele that increases plasma ACE2 also increases ACE (**Figure S23B**). We used 17 independent cis-pQTLs as instruments according to M. Pigeyre et al.' study[17].

When conducting the same set of causal inference and genetic correlation analyses in Figure 3 for ACE instead of ACE2, we observed a clear difference between the two proteins in terms of their connections with vascular phenotypes (**Figure S24**, **Table S16 - S18**). For this analysis, the GWAS summary statistics of ACE was from E. Ferkingstad et al.' study[18], and we took 14 significant SNPs overlaped with above 17 SNPs as instruments to conduct cis-pQTL-based MR. As a well-established drug target for hypertension, both GSMR and cis-pQTL-based MR suggest that genetically

elevated ACE levels had a causal role on high blood pressure. In contrast, as we mentioned in the main Results, ACE2's effects are mainly on CVD-related phenotypes instead of blood pressure traits.

Utilizing the power of both ACE and ACE2 cis-pQTL effects, we further investigated the downstream effects of these two proteins on cardiovascular and blood pressure phenotypes in the IEU Open GWAS Project database (see **Data availability**). Across CVD-related traits, the downstream effects of the protein-increasing allele are significantly negatively correlated between ACE and ACE2 (**Figure S23C**, **Table S19 - S20**). Such relationship could not be detected if also considering blood pressure traits, consistent with our results that ACE2 has no significant causal effect on blood pressure phenotypes.

Detailed explanations of the annotated phenotypes in Figure 2

The quoted trait names below match the names in **Table S9**. Since the GWAS summary statistics were obtained from the UK Biobank analysis, please refer to the UK Biobank data showcase (`https://biobank.ctsu.ox.ac.uk/crystal/index.cgi`) for further details.

Wheeze or whistling in the chest: 'Wheeze or whistling in the chest in last year', wheezing is the harsh whistle or rough rattle you hear when your airway is partially blocked. It may be blocked due to colds, allergic reactions, bronchitis or allergies. Wheezing is also a symptom of asthma, pneumonia, and heart failure.

SBP: 'Systolic blood pressure (automated reading)'

None vascular/heart problems diagnosed by doctor: 'Vascular/heart problems diagnosed by doctor: None of the above', none of the asked vascular/heart problems, i.e. angina, heart attack, high blood pressure ..., has been diagnosed by doctor before.

Hypertension: 'Vascular/heart problems diagnosed by doctor: High blood pressure' - left, '(Non-cancer) self-reported: hypertension' - right.

CRP: 'C-reactive protein', a cyclic pentameric protein found in plasma, the circulating concentration of which increases with inflammation. It is a liver-derived acute-phase protein that is increased after interleukin-6 secretion by macrophages and T cells.

None blood pressure medication: 'Medication for cholesterol, blood pressure or diabetes: None of the above', none of the asked medication, i.e. blood pressure medication, insulin ..., was taken.

Blood pressure medication: 'Medication for cholesterol, blood pressure, diabetes, or take exogenous hormones: Blood pressure medication' – left, 'Medication for cholesterol, blood pressure or diabetes: Blood pressure medication' – right.

Amlodipine: ‘Treatment/medication code: amlodipine’, Amlodipine is used alone or in combination with other medications to treat high blood pressure in adults and children 6 years and older. It is also used to treat certain types of angina (chest pain) and coronary artery disease.

Leg fat: ‘Leg fat percentage (left)’ - left, ‘Leg fat percentage (right)’ – right.

Usual walking pace: ‘Usual walking pace’, question ”How would you describe your usual walking pace?” was asked. If the participant activated the Help button, they were shown the message: Slow pace is defined as less than 3 miles per hour. Steady average pace is defined as between 3-4 miles per hour. Fast pace is defined as more than 4 miles per hour.

FVC: ‘Forced vital capacity (FVC), Best meassure’

Arm fat: ‘Arm fat mass (right)’ - left, ‘Arm fat mass (left)’ – right

Fluid intelligence: ‘Fluid intelligence score’, This is a simple unweighted sum of the number of correct answers given to the 13 fluid intelligence questions. Participants who did not answer all of the questions within the allotted 2-minute limit are scored as zero for each of the unattempted questions.

Red wine (weekly): ‘Average weekly red wine intake’, question ”In an average WEEK, how many glasses of RED wine would you drink? (There are six glasses in an average bottle)” was asked.

Current tobacco smoking: ‘Current tobacco smoking’ - left, ‘Smoking status: Current’ – right

Alcohol with meals: ‘Alcohol usually taken with meals’, question ”When you drink alcohol is it usually with meals?” was asked.

Qualifications: from left to right, ‘Qualifications: Other professional qualifications eg: nursing, teaching’, ‘Qualifications: O levels/GCSEs or equivalent’, ‘Qualifications: College or University degree’

Years of schooling: ‘Years of schooling 2016’

3 SUPPLEMENTAL FIGURES

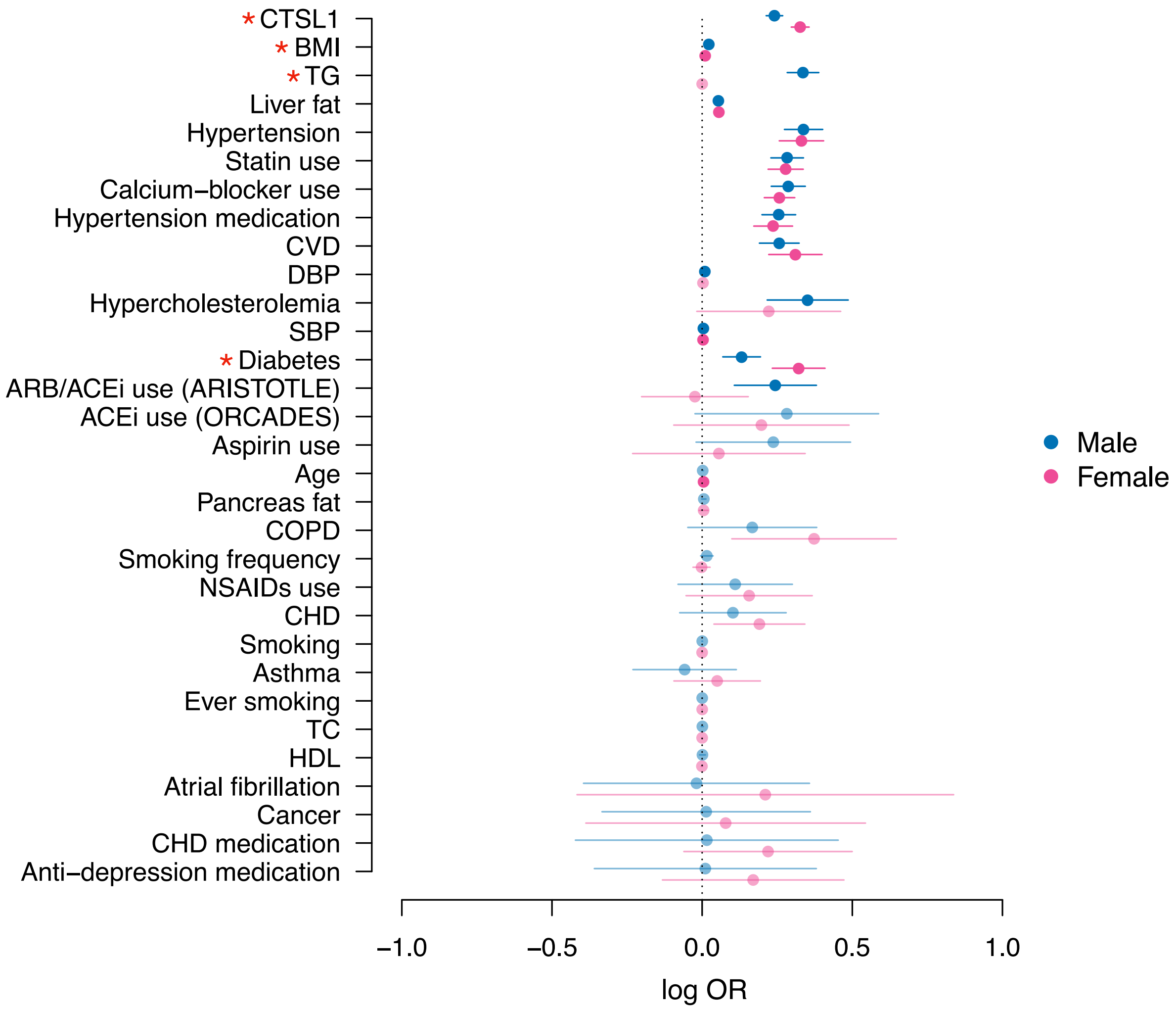

Figure S1: Phenotypic associations between plasma ACE2 and various conditions. Each log odds ratio (OR) was estimated via a univariate linear regression of plasma ACE2 on each condition. The error bars represent 95% confidence intervals. Estimates that passed a Bonferroni-corrected 5% p-value threshold are shown in bright colors, otherwise transparent. The red stars on the y-axis labels indicate statistically significant sex heterogeneity in the estimated effects at a Bonferroni-corrected 5% p-value threshold. CTSL1: Cathepsin L1, BMI: body mass index, TG: triglycerides, CVD: cardiovascular disease, DBP: diastolic blood pressure, SBP: systolic blood pressure, ARB: angiotensin receptor blocker, ACEi: angiotensin-converting enzyme (ACE) inhibitor, COPD: chronic obstructive pulmonary disease, NSAIDs: non-steroidal anti-inflammatory drugs, CHD: coronary heart disease, TC: total cholesterol, HDL: high-density lipoprotein.

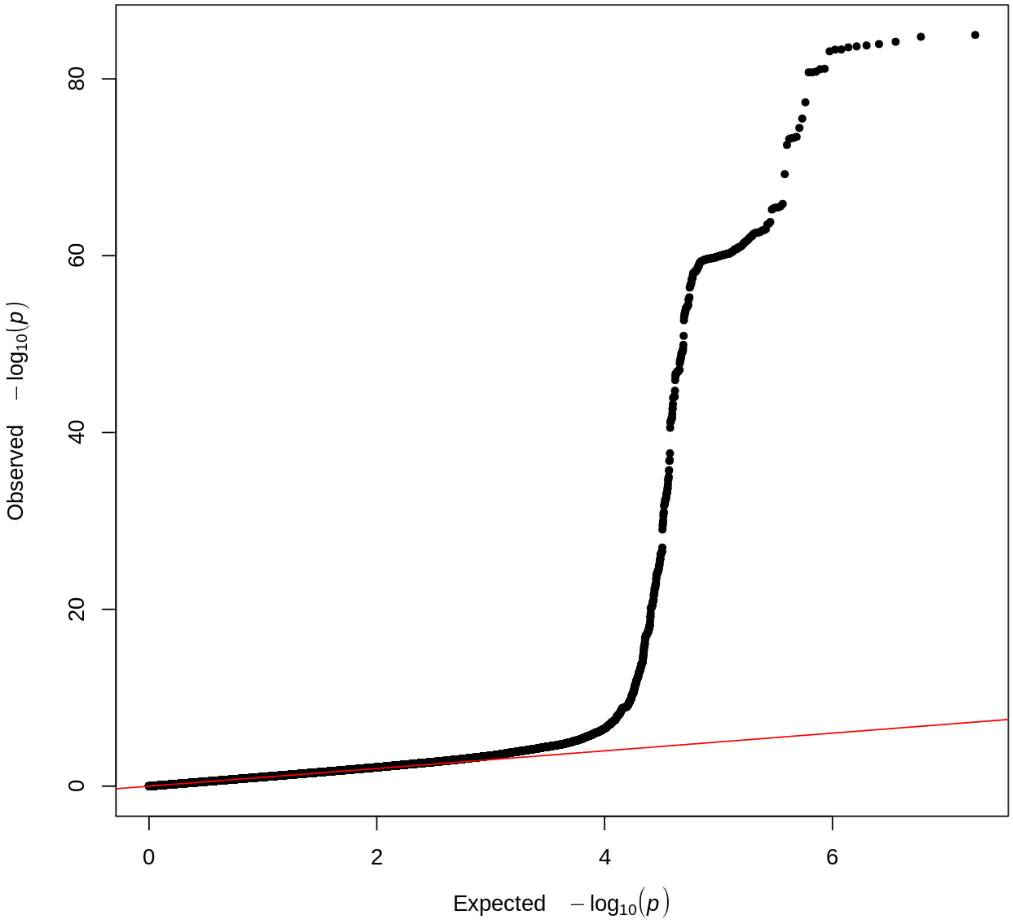

Figure S2: Quantile-quantile plot of the ACE2 GWAS $-\log_{10} P$ values.

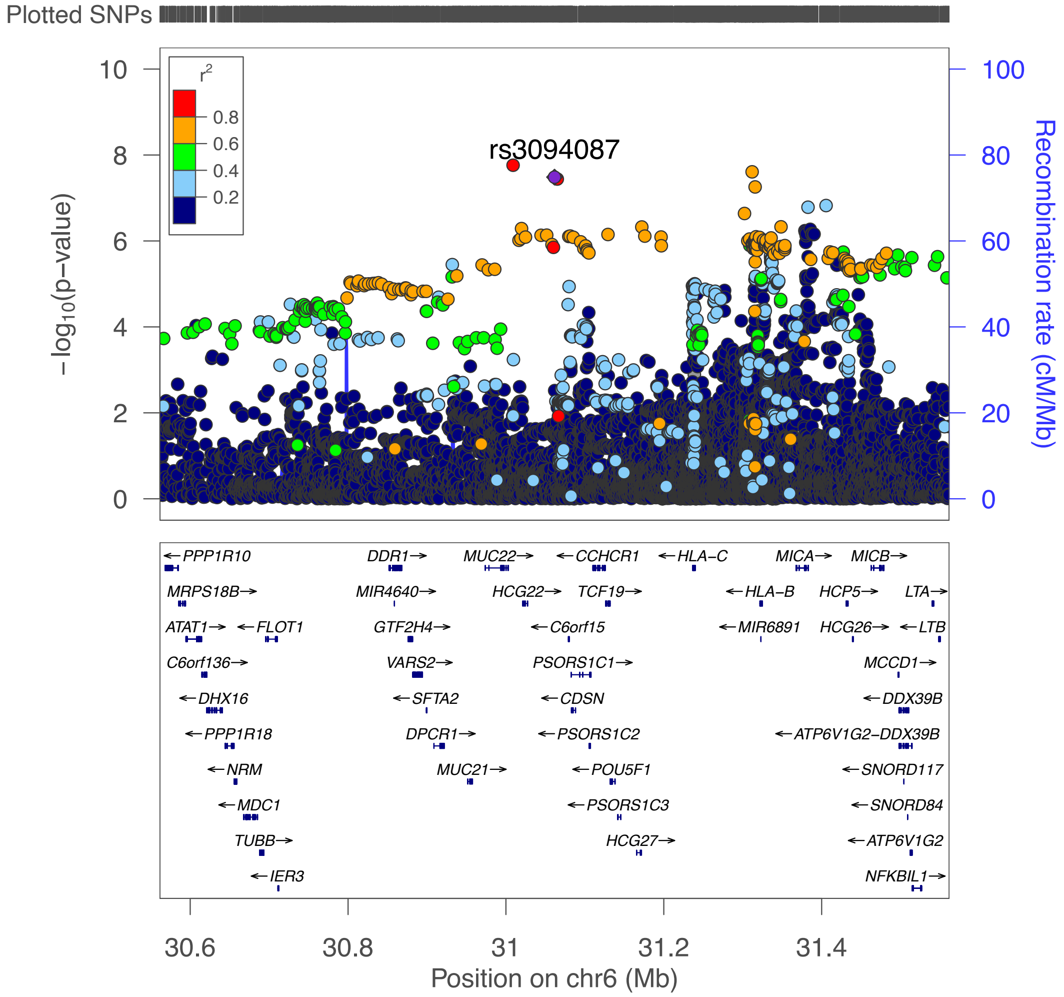

Figure S3: Regional plot for the rs3094087 locus.

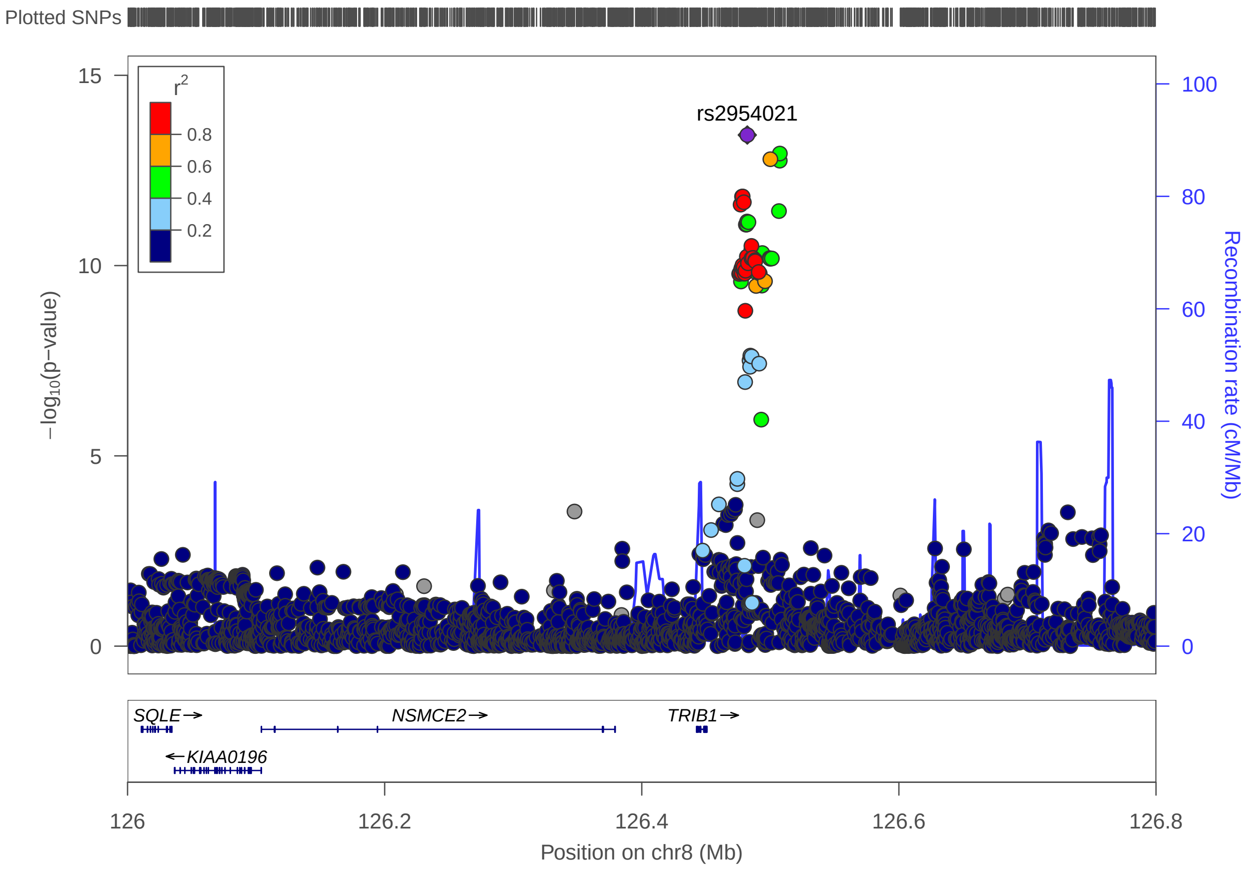

Figure S4: Regional plot for the rs2954021 locus.

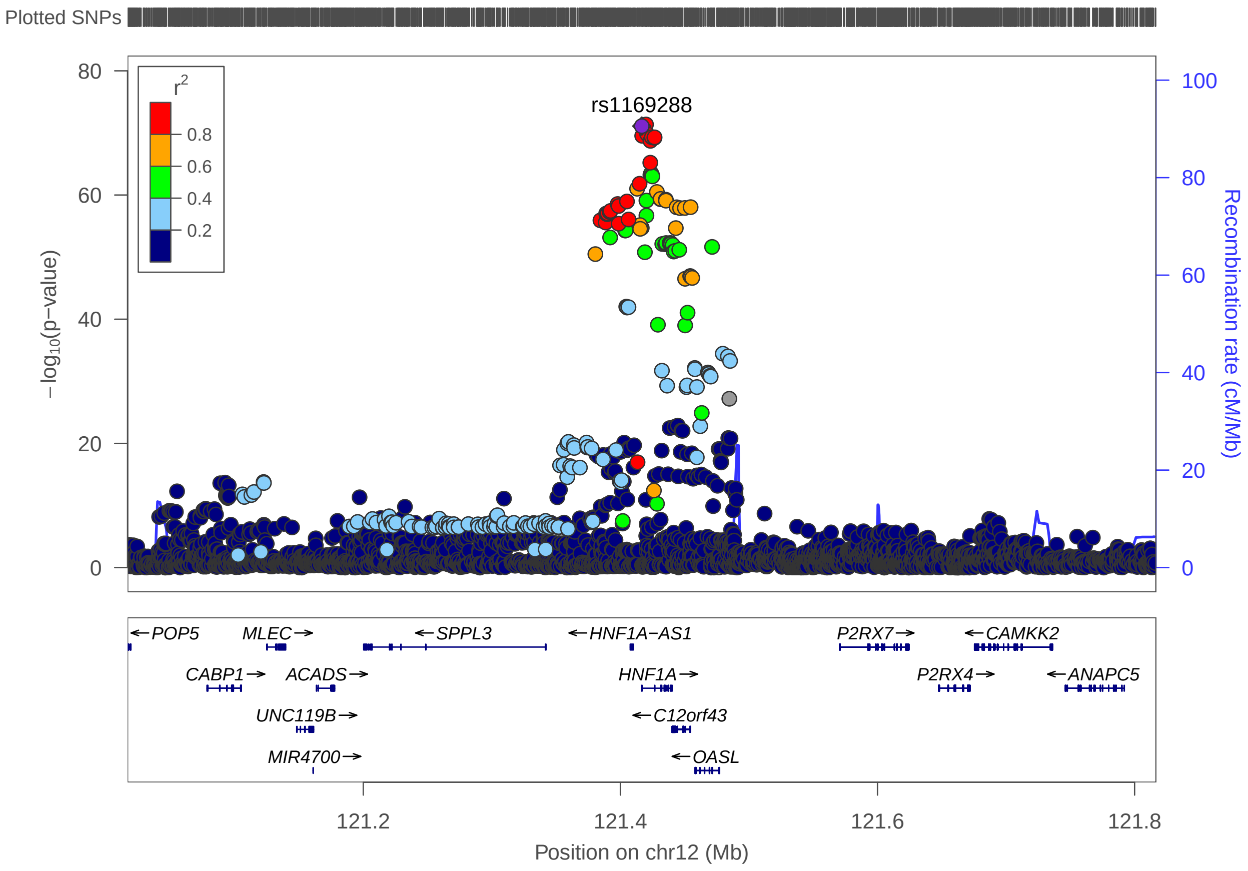

Figure S5: Regional plot for the rs1169288 locus.

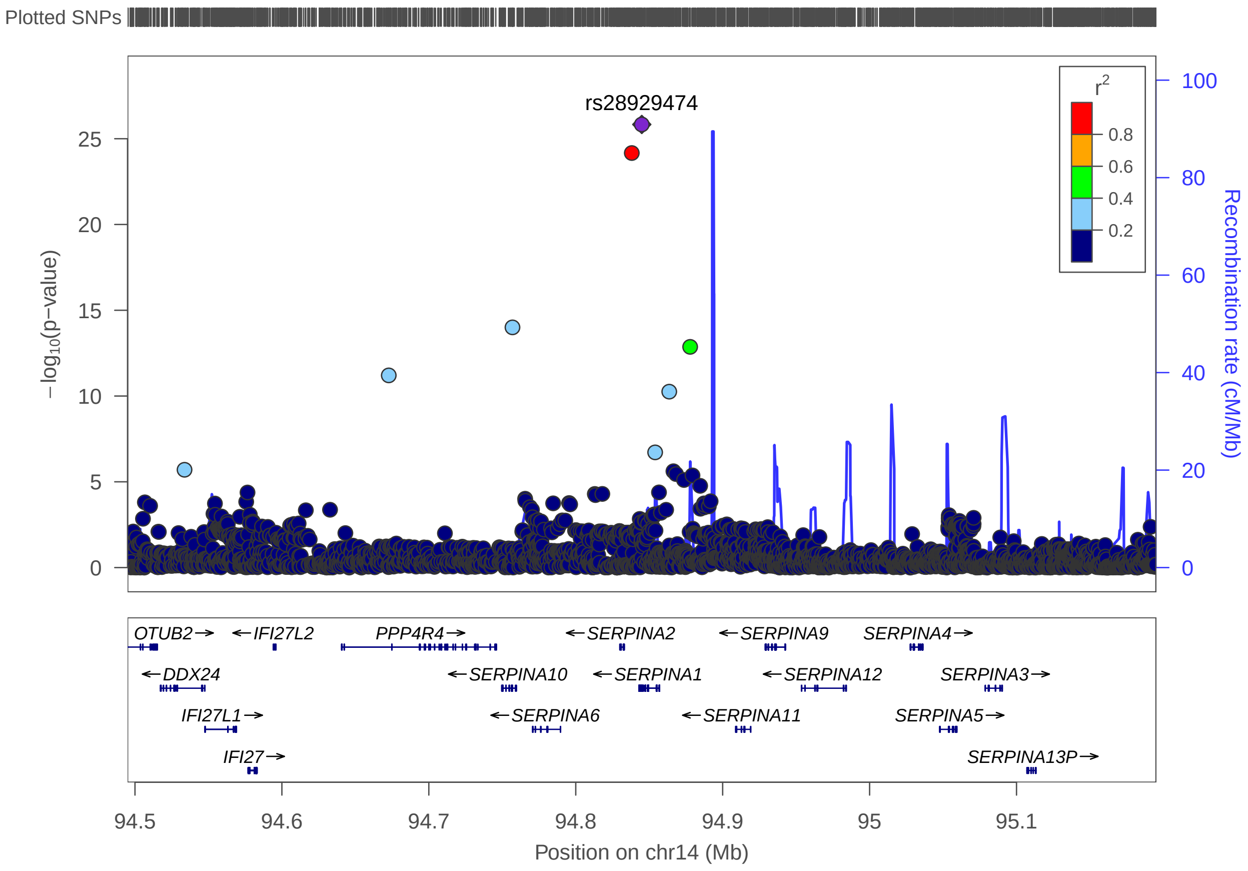

Figure S6: Regional plot for the rs28929474 locus.

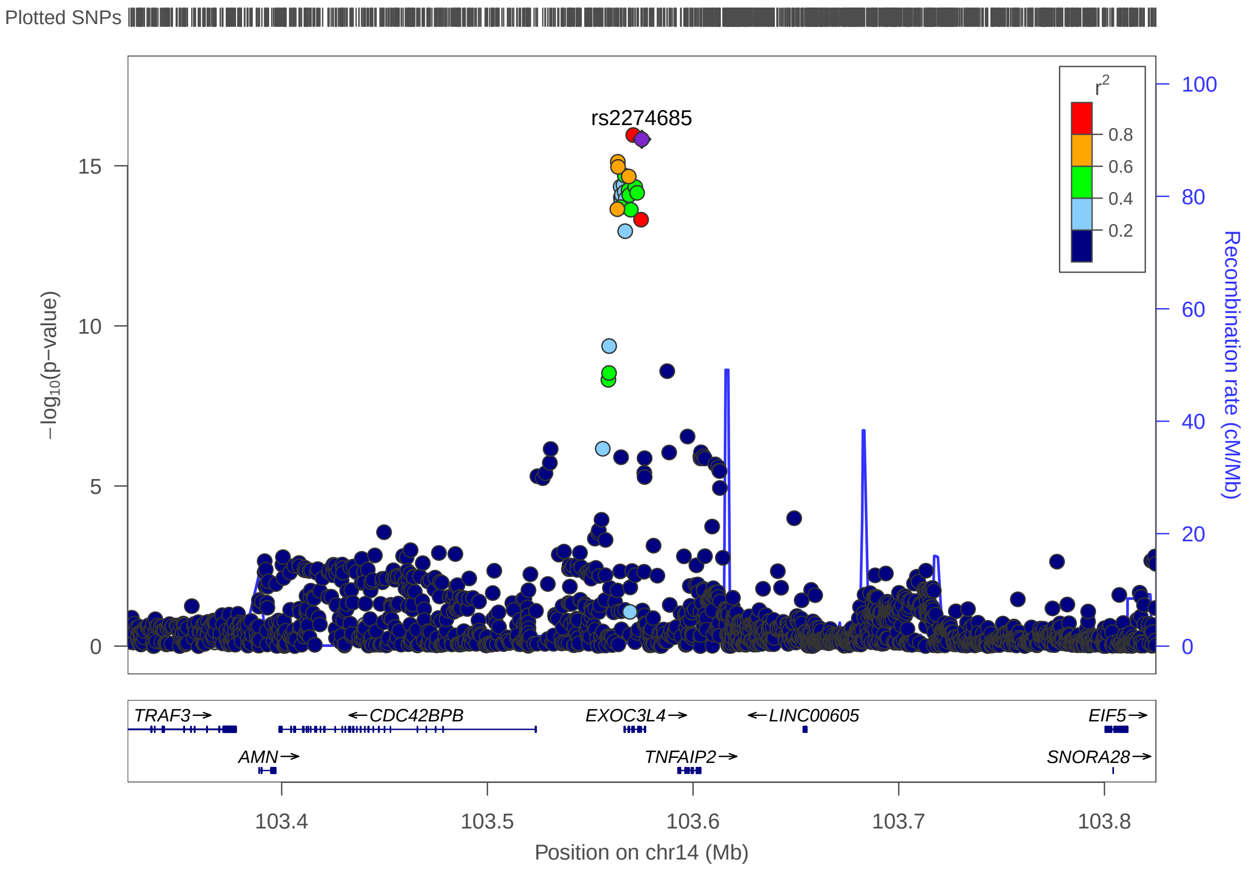

Figure S7: Regional plot for the rs2274685 locus.

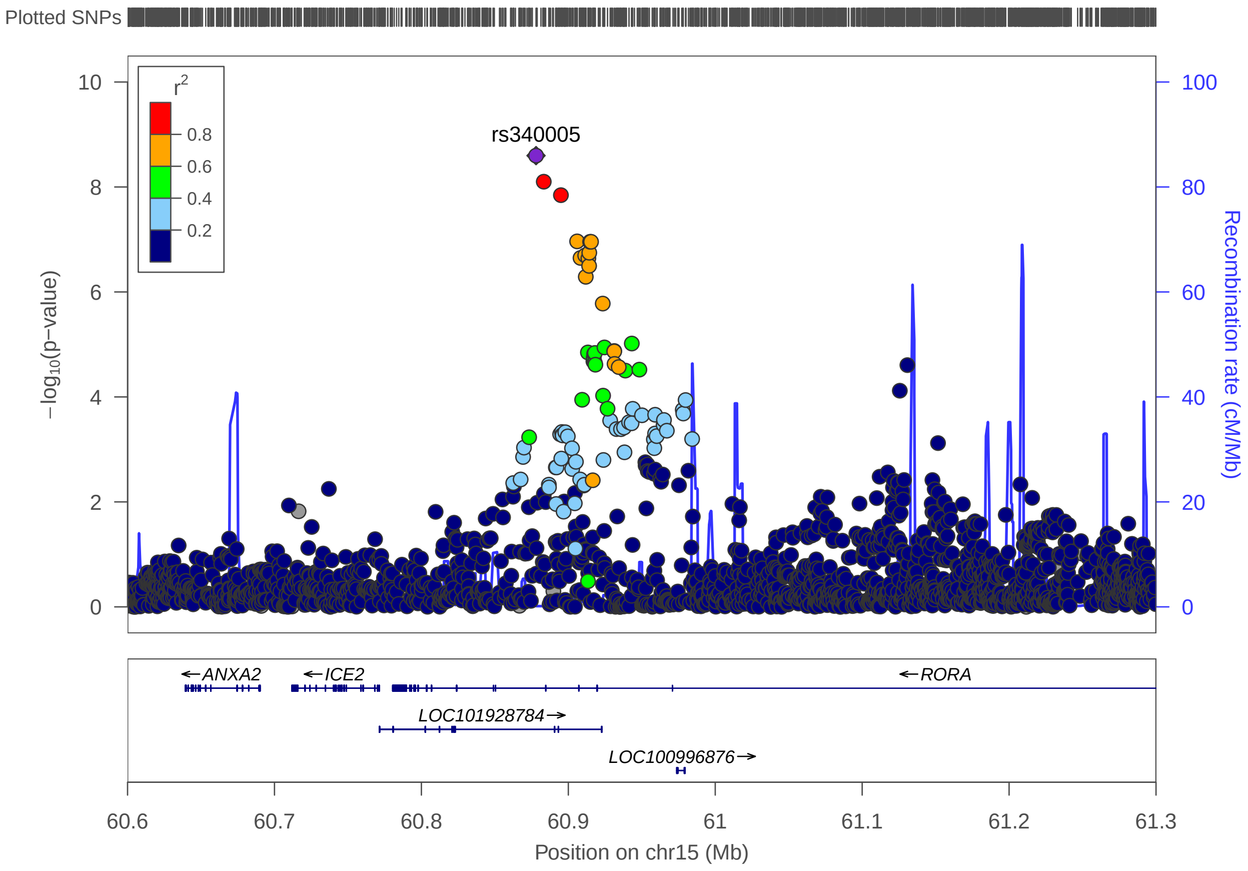

Figure S8: Regional plot for the rs340005 locus.

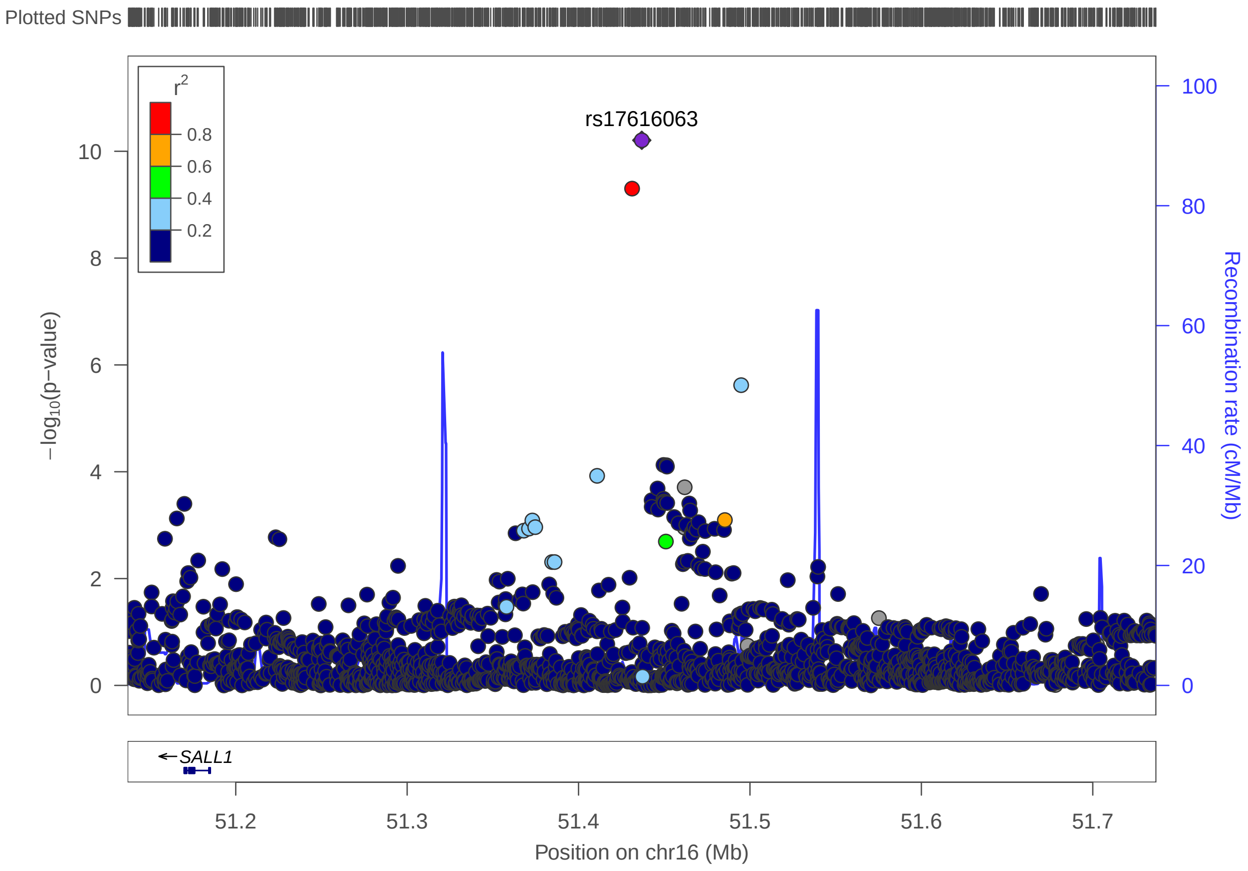

Figure S9: Regional plot for the rs17616063 locus.

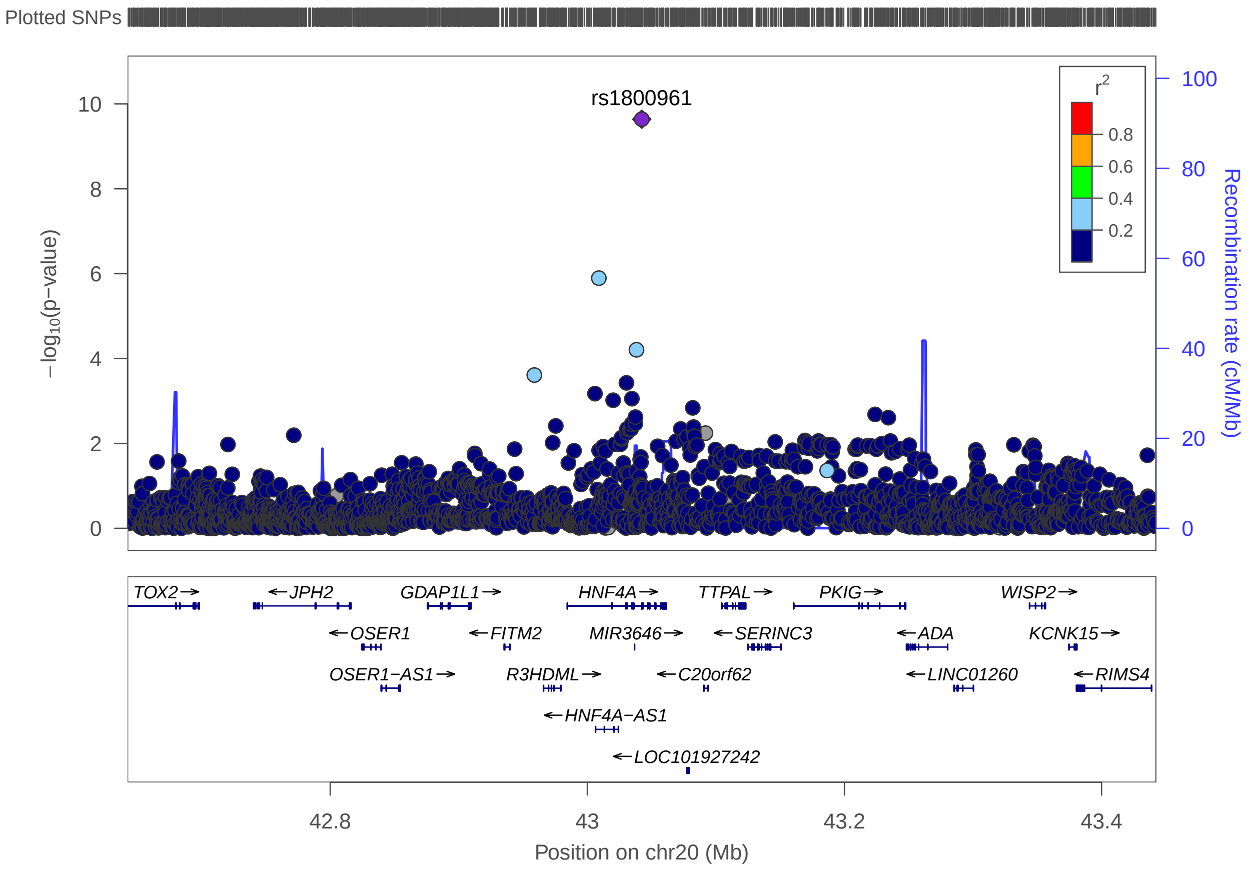

Figure S10: Regional plot for the rs1800961 locus.

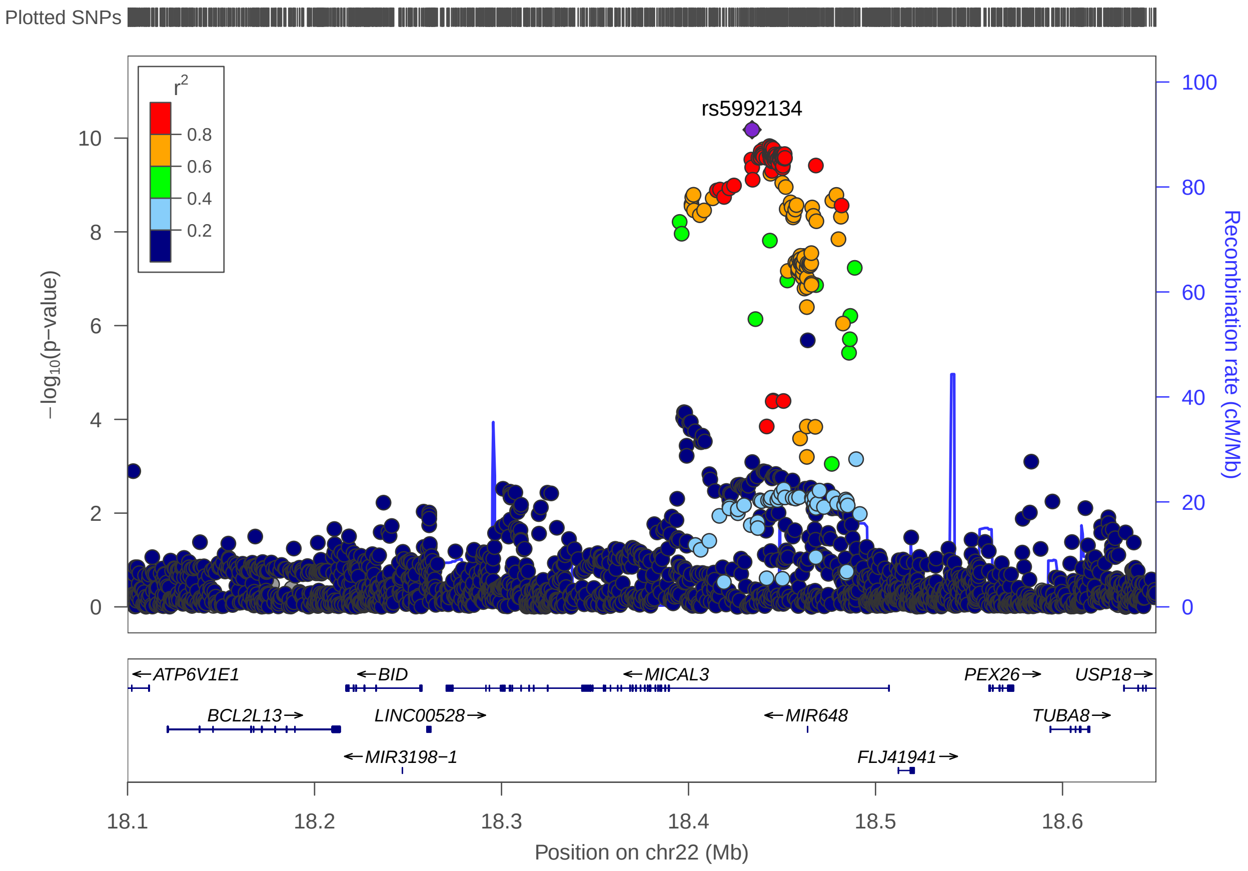

Figure S11: Regional plot for the rs5992134 locus.

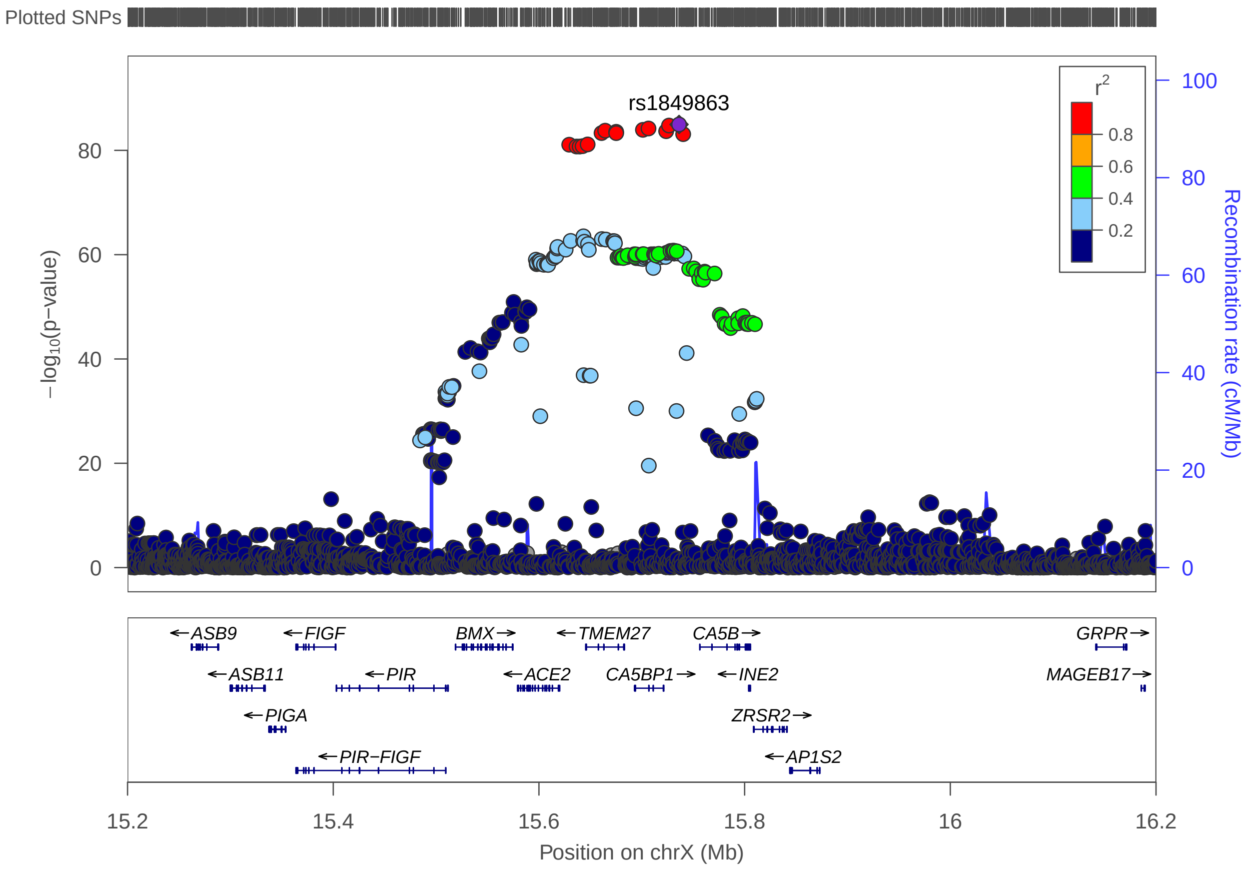

Figure S12: Regional plot for the rs1849863 locus.

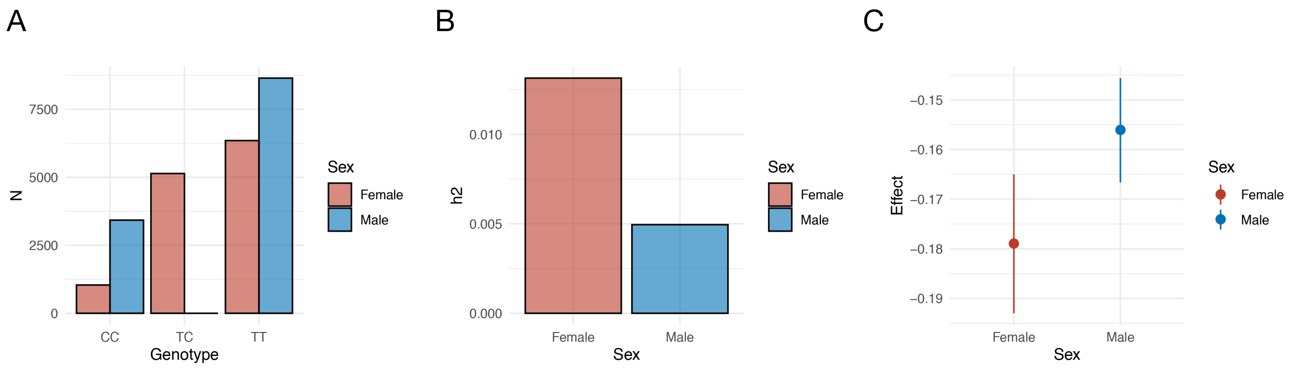

Figure S13: Sex heterogeneity of the ACE2 cis-pQTL genotype-phenotype map. (**A**) Sample size distributions of males and females across the ACE2 cis-pQTL genotypes. (**B**) Narrow-sense heritability captured by the ACE2 cis-pQTL for males and females. (**C**) Estimated allelic substitution effect of the ACE2 cis-pQTL for males and females, respectively.

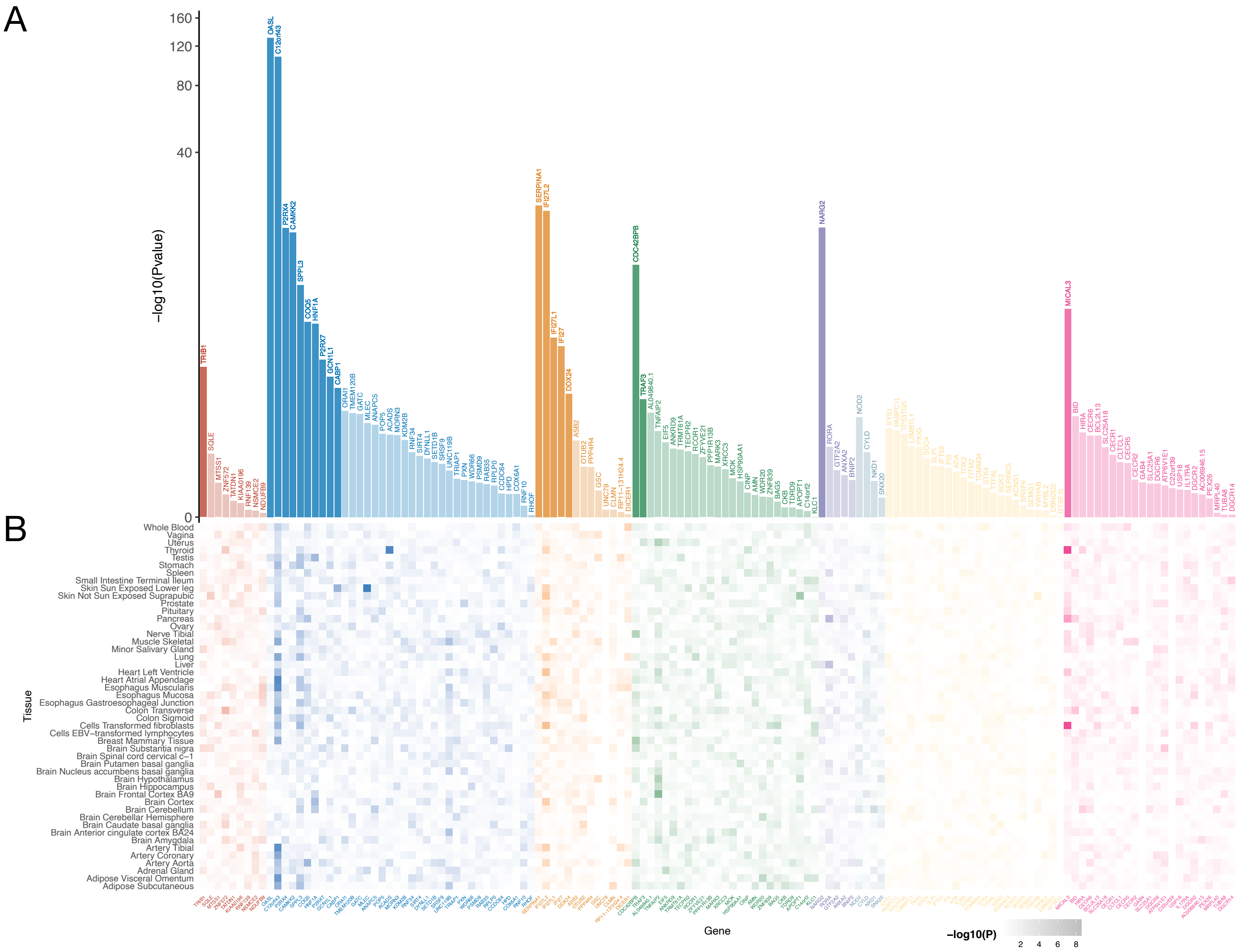

Figure S14: cis-eQTL associations of the trans-pQTL of plasma ACE2. The lead variant at each mapped trans-pQTL is used to represent the pQTL association. Besides the MHC, the other eight trans-pQTL are shown in different colours. (**A**) cis-eQTL association signals of the eQTLGen analysis, where the genome-wide significant eQTL associations are highlighted in dark colours. (**B**) cis-eQTL association signals of the GTEx analysis across multiple human tissues.

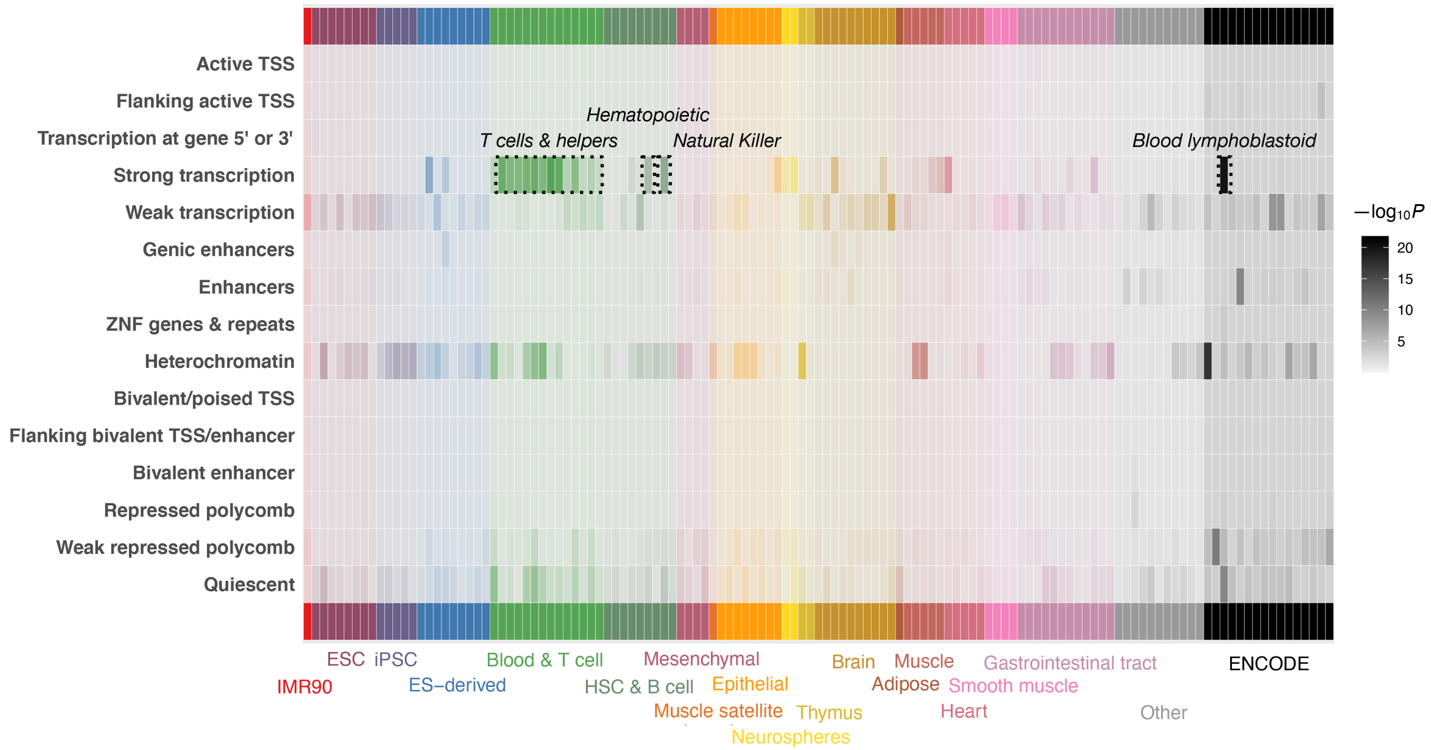

Figure S15: Enrichment of ACE2 genetic associations across chromatin states in different tissues and cell types. Genome-wide association true-false status of $P < 5 \times 10^{-8}$ for ACE2 were logistic-regressed on the chromatin states values to test for enrichment signals, including the LD scores of the genetic variants to account for linkage. TSS: transcription start sites. See Data Availability for detailed descriptions of the tissues and cell types.

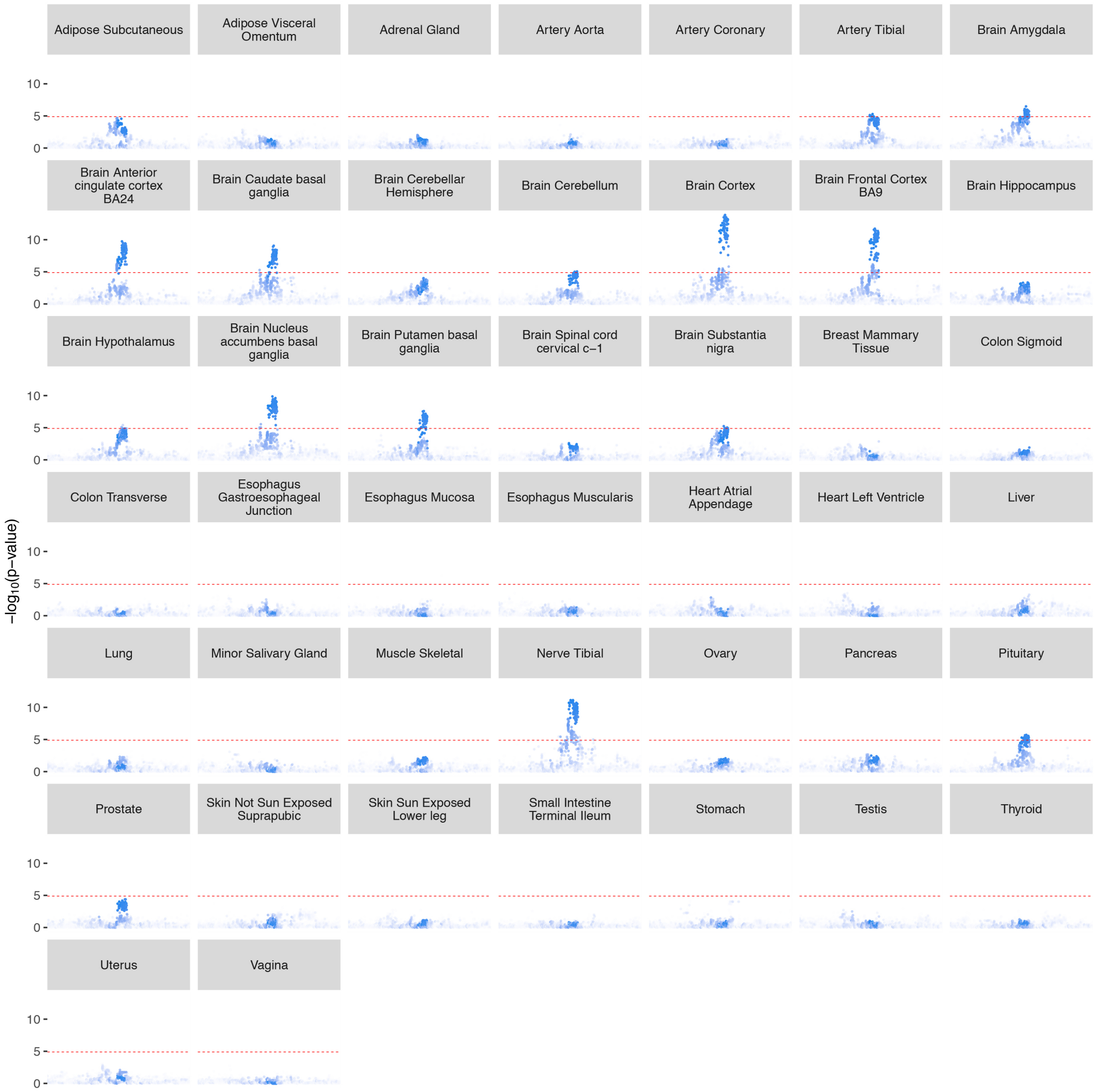

Figure S16: ACE2 cis-eQTL signals across 44 human tissues. The data were extracted from the GTEx project portal. The cis-window was defined as a 1 Mb window around the transcription starting site of ACE2. The common SNP upstream of ACE2 that is closest to the transcription starting site, X:15618063 (Ch37 reference, minor allele frequency = 0.4572, distance to TSS = -2208 bp), was considered a reference for linkage disequilibrium R^2 calculation and colouring of the other SNPs in the cis-window.

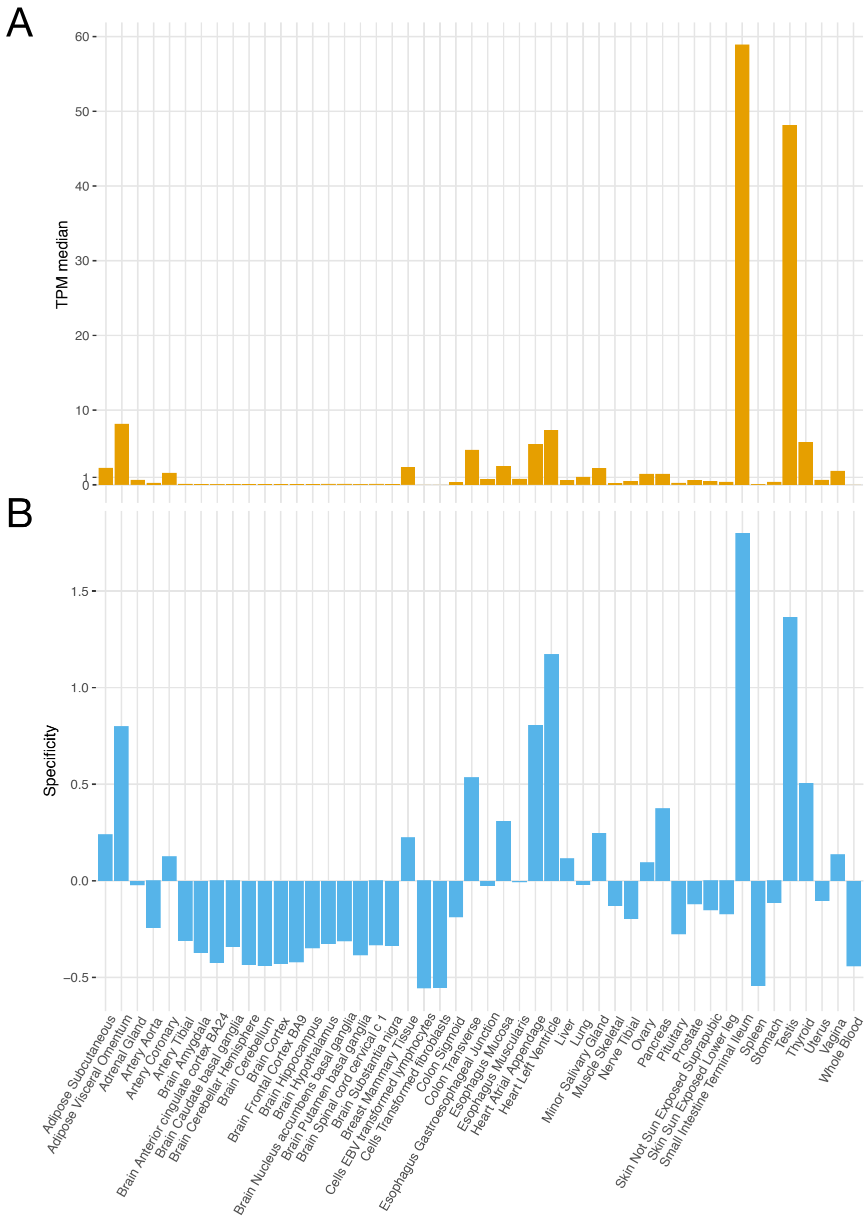

Figure S17: ACE2 expression and specificity across human tissues. The expression data were extracted from the GTEx project portal. (**A**) Medians of the gene expression values across samples in transcript counts per million reads. (**B**) Tissue-specificity of the gene expression normalised for both the distribution across samples and that across all the genes.

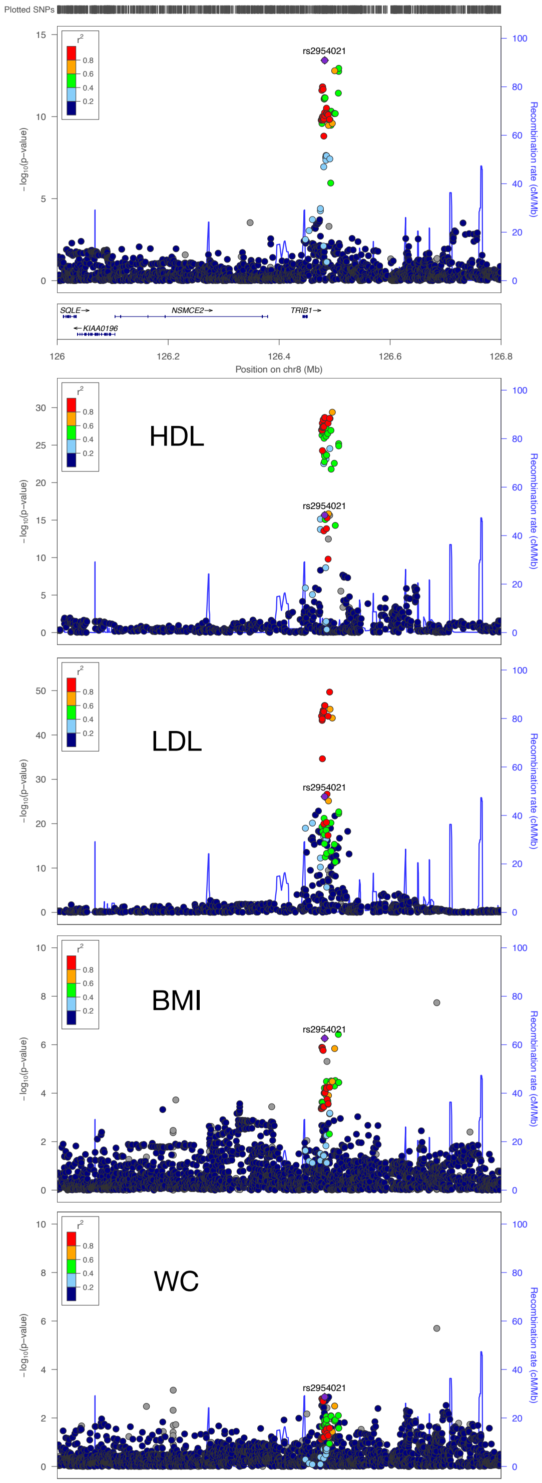

Figure S18: colocalisation of TRIB1 locus on blood lipids, and waist circumference. LDL: low-density lipoprotein, HDL: high-density lipoprotein, BMI: body mass index, WC: waist circumference.

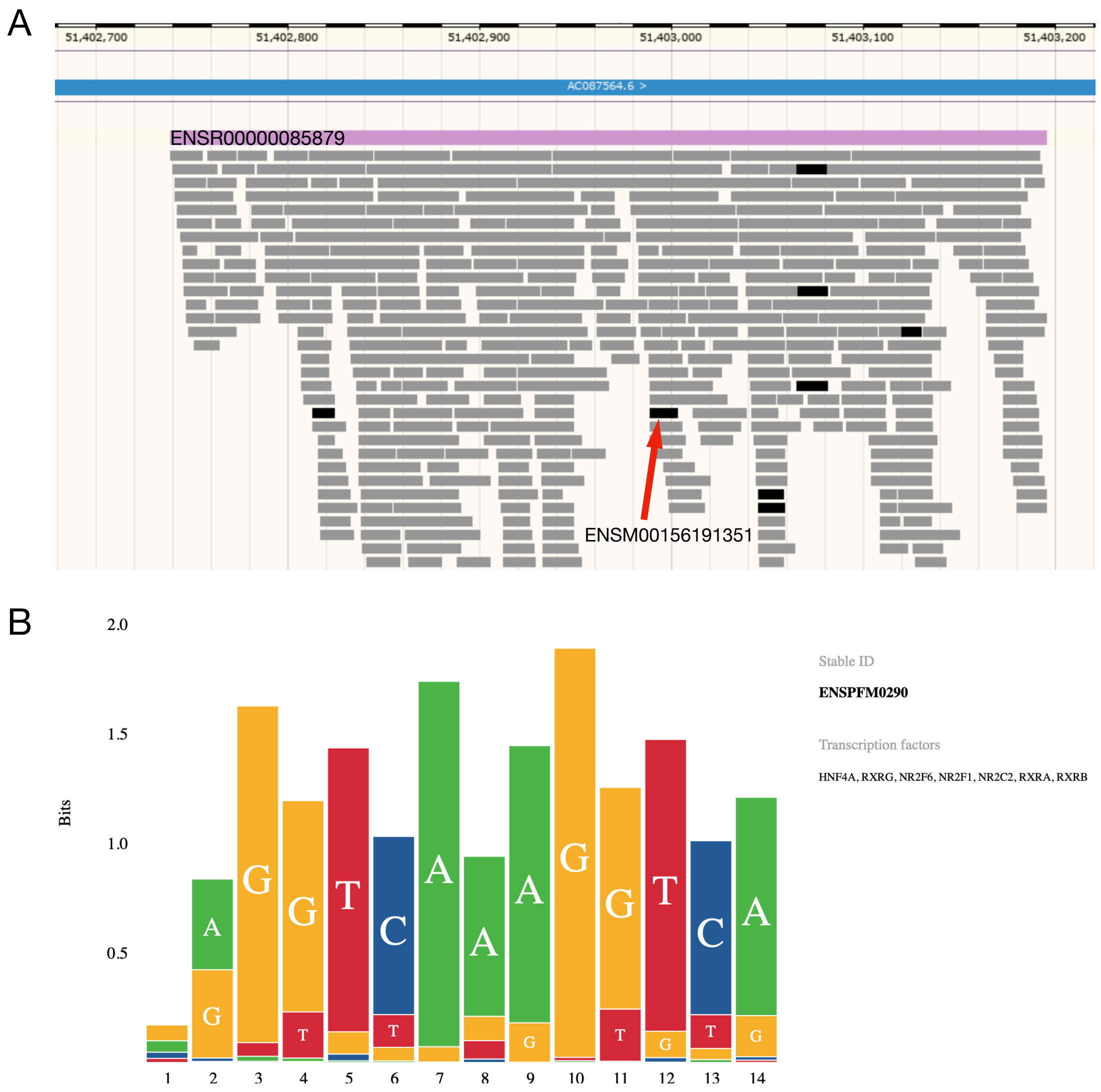

Figure S19: *HNF4A* binds on locus rs17616063 as a transcription factor (TF) (**A**) rs17616063 is located on the TF binding site ENSR00000085879 and the nearest experimentally verified motif ENSM00156191351 is shown. (**B**) The position weight matrix (PWM) ENSPFM0290 offered by ENCODE project corresponds to the motif ENSM00156191351. Protein *HNF4A* is one of the target binding TFs in HepG2 cell line.

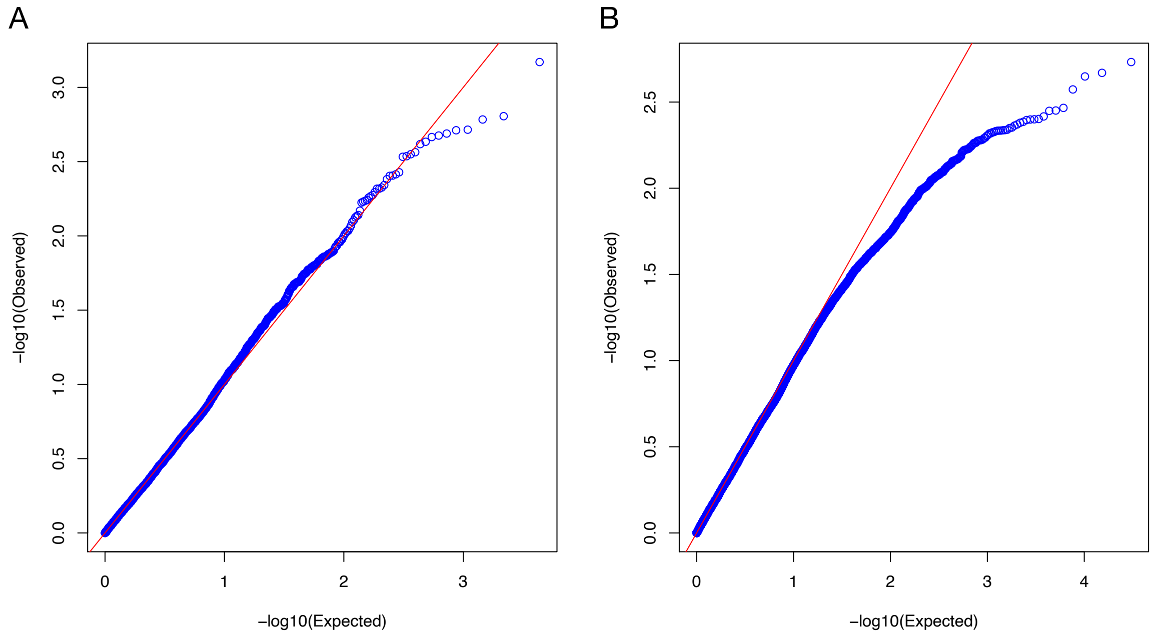

Figure S20: QQ-plots testing the ACE2 cis-eQTL effects on UK Biobank phenotypes. The common SNP upstream of ACE2 that is closest to the transcription starting site, X:15618063, was testing against 3,990 UK Biobank phenotypes, based on the Neale's lab protocol. (**A**) The QQ-plot for the marker-trait associations from UK Biobank GWAS. (**B**) The QQ-plot for testing the expression-trait Mendelian randomization causal effects with the marker as a single instrument.

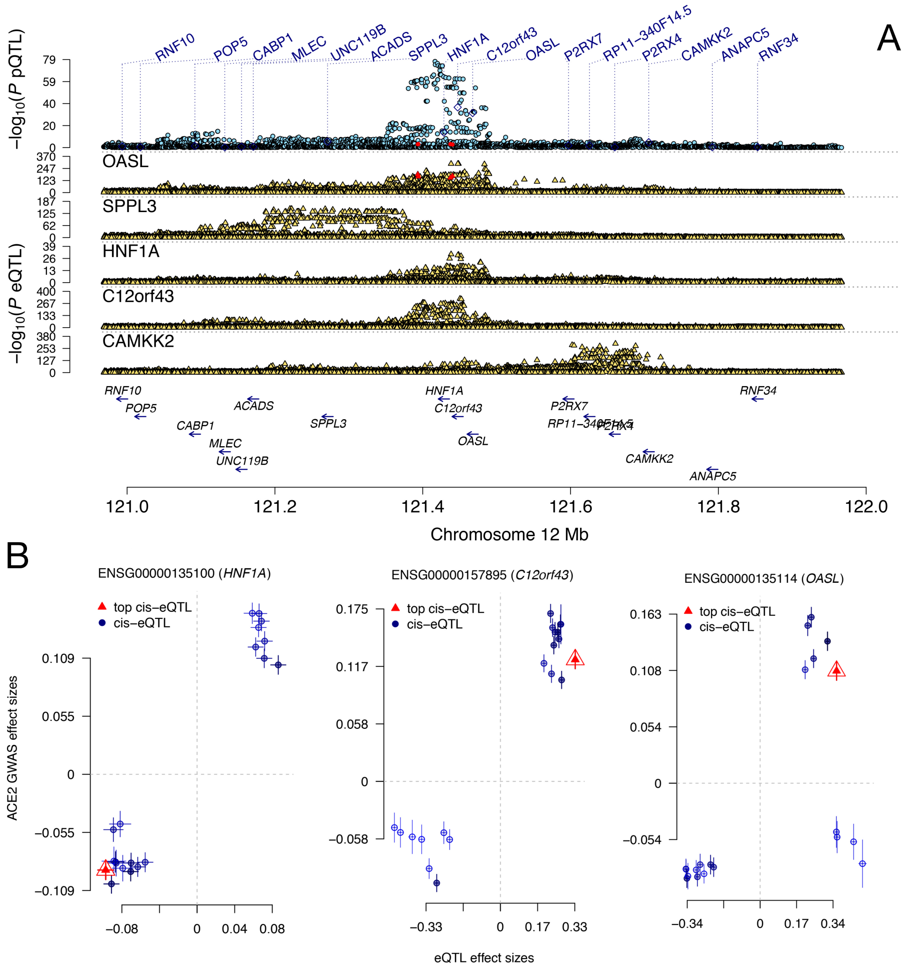

Figure S21: Colocalisation between cis-eQTL of *HNF1A* and *C12orf43* and the corresponding trans-pQTL of ACE2. (**A**) Comparison between the ACE2 trans-pQTL and the cis-eQTL of multiple genes in the region based on eQTLGen summary data. (**B**) Visualisation of the heterogeneity of multiple SNP effects between three eQTL and the ACE2 trans-pQTL, where the SNPs were LD-clumped by the HEIDI procedure. The SNPs showing heterogeneous effects for *OASL* expression are also highlighted in (A).

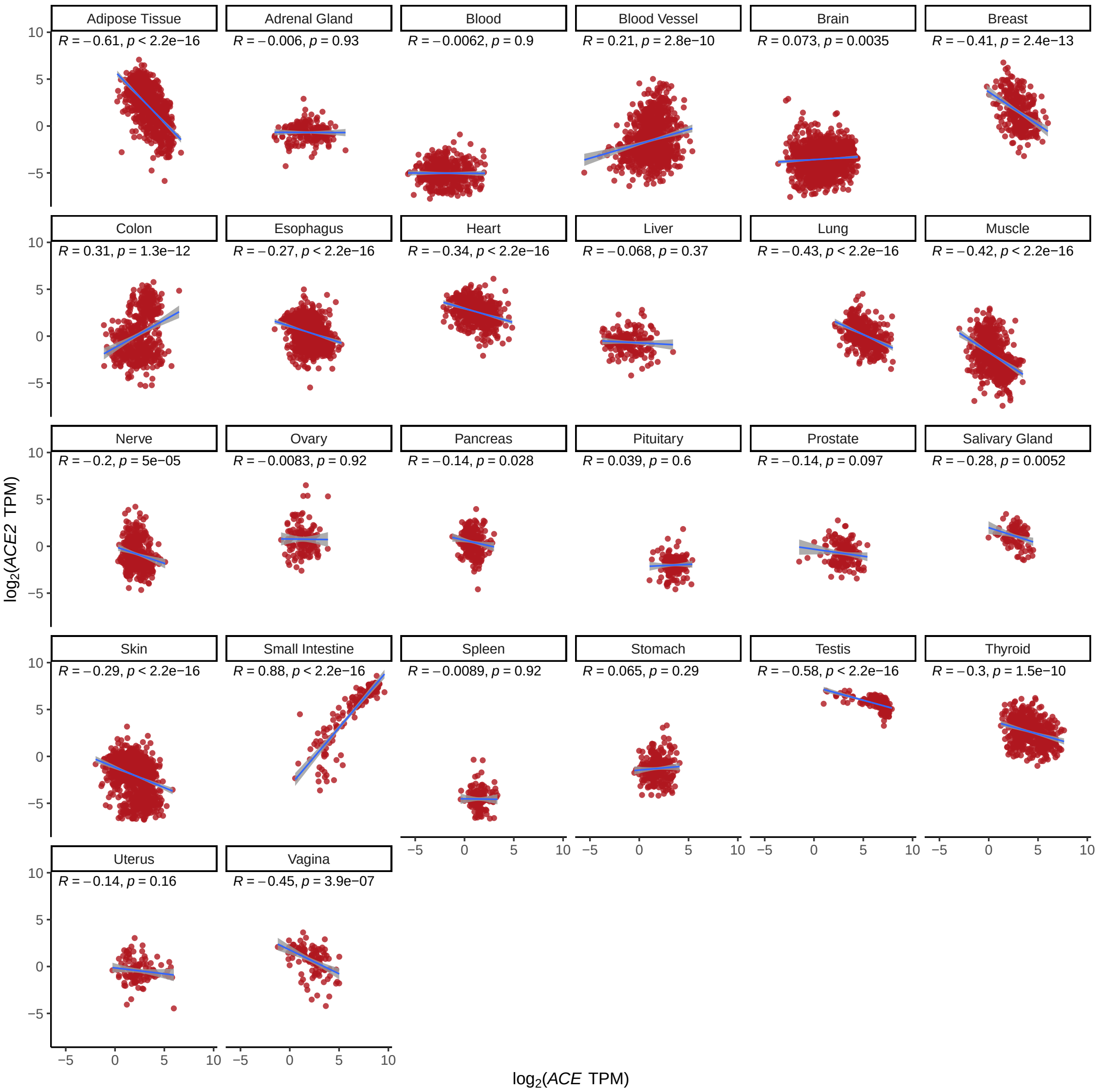

Figure S22: Relation of *ACE* and *ACE2* expression across human tissues The RNA-seq data of ACE and ACE2 obtained from GTEx project V7 across 26 tissues. The raw TPM values were performed log_2 scale transformation. Blue lines are linear regression lines.

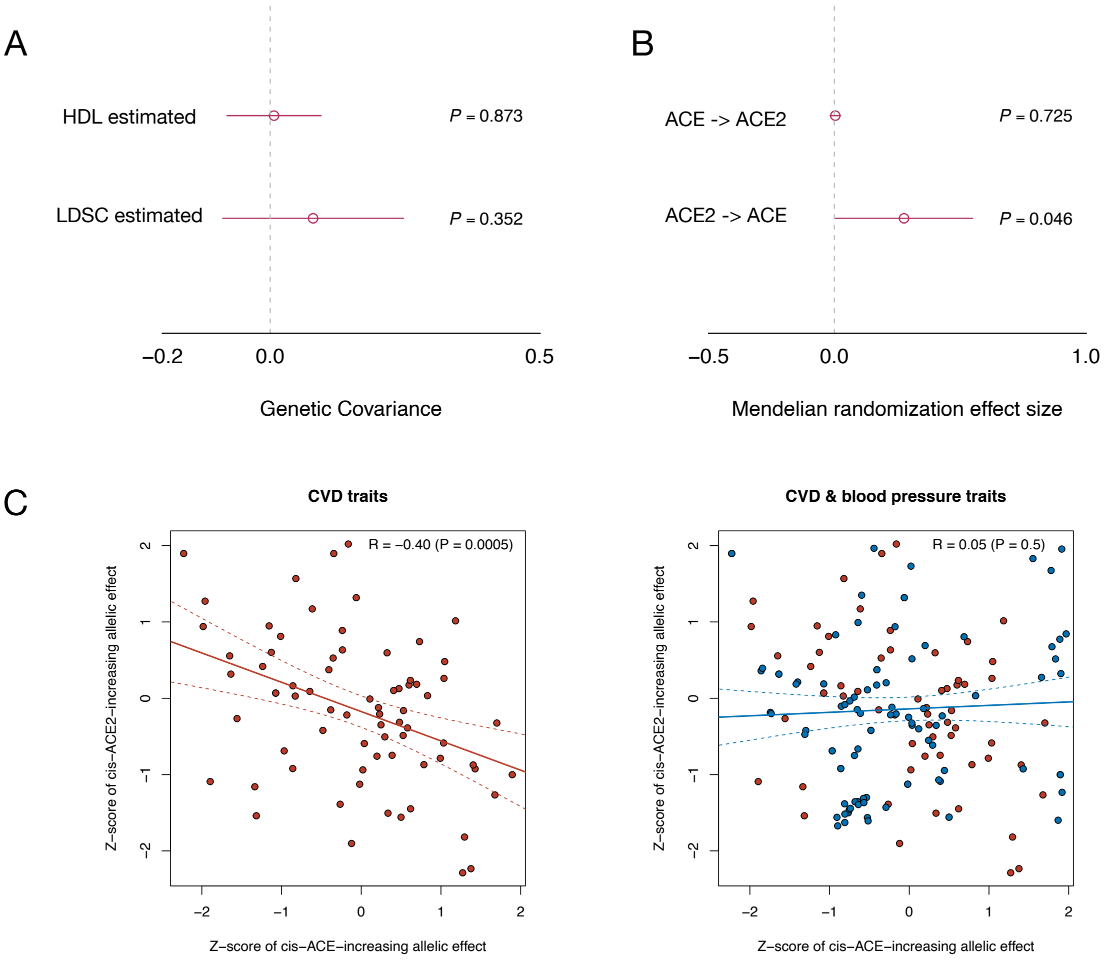

Figure S23: Genetic relationship between plasma ACE and ACE2 The figure shown the genetic covariance between plasma ACE and ACE2, cis-region mendelian randomization (MR) analysis and their causal effects on cardiovascular disease (CVD) and blood pressure traits. The error bar represents 95% confidence interval. (**A**) the genetic covariance between plasma ACE and ACE2 estimated by method high-definition likelihood (HDL) and LD Score Regression (LDSC) respectively. (**B**) Cis-SNPs based MR of effect of ACE concentration change on ACE2 concentration and effect of ACE2 change on ACE. (**C**) MR analysis was performed on ACE and ACE2 concentration to different CVD and blood pressure traits using top cis-SNP as instrument. Z-score obtained from MR's effect size and standard error. Red dots represent effect on CVD traits and blue represent effect on blood pressure traits.

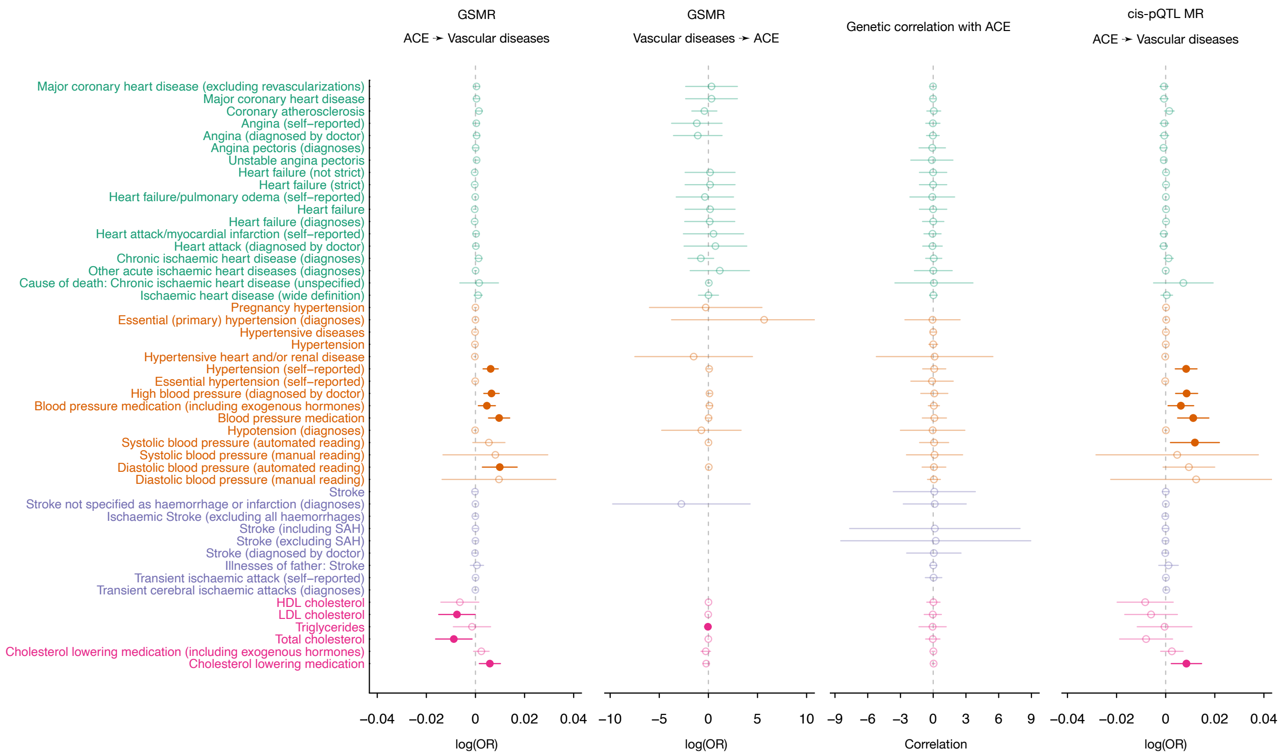

Figure S24: Genetic and causal relationships between plasma ACE and vascular diseases. Estimates significantly (Bonferroni Correction) different from zero are highlighted in filled circles. The first two forest plots show the bidirectional generalised summary-level Mendelian randomization (GSMR) analysis results between plasma ACE and 48 vascular-disease-related traits. The third forest plot gives the corresponding genetic correlations estimates between plasma ACE and these phenotypes. The last forest plot shows the estimated MR effects based on cis-pQTL only. The error bar represents 95% confidence interval. OR: odds ratio.

4 SUPPLEMENTAL TABLES

Table S1: Summary of participating cohorts.

[See the Excel File]

Table S2: Cohort-specific sex and age effects on plasma ACE2.

[See the Excel File]

Table S3: Simple linear regression revealed phenotypic relation between plasma ACE2 and different conditions.

[See the Excel File]

Table S4: Detailed summary of ten genome-wide significant loci for plasma ACE2 by FUMA.

[See the Excel File]

Table S5: Cis-eQTL association signals of trans-pQTLs from the eQTLGen consortium.

[See the Excel File]

Table S6: Cis-eQTL association signals of trans-pQTLs from GTEx consortium.

[See the Excel File]

Table S7: Enrichment of ACE2 genetic associations across chromatin states in different tissues and cell types.

[See the Excel File]

Table S8: Significant associations of the ACE2 pQTL with complex traits using PhenoScanner. Results with false discovery rate < 0.05 are listed.

[See the Excel File]

Table S9: Genetic correlation estimates between plasma ACE2 and complex traits using the high-definition likelihood method. The analysis was performed across severe COVID-19, C-Reactive Protein(CRP), and the 821 phenotypes from LD-Hub. We filtered out those traits with estimated zero heritability remaining 724 traits.

[See the Excel File]

Table S10: Description of the GWAS summary statistics for the 48 selected vascular disease phenotypes from the UK Biobank.

[See the Excel File]

Table S11: Genetic correlation estimates between plasma ACE2 and 48 vascular disease phenotypes.

[See the Excel File]

Table S12: Bidirectional generalised summary-level Mendelian randomization analysis between plasma ACE2 and 48 vascular disease phenotypes.

[See the Excel File]

Table S13: Cis-locus MR analysis for plasma ACE2 and 48 vascular disease phenotypes.

[See the Excel File]

Table S14: 96 antigens used to test for the ACE2 assay specificity.

[See the Excel File]

Table S15: Relation of ACE and ACE2 expression across human tissues in GTEx project. Raw TPM values were transformed in to log2 scale for each tissue.

[See the Excel File]

Table S16: Genetic correlation estimates between plasma ACE and 48 vascular disease phenotypes.

[See the Excel File]

Table S17: Bidirectional generalised summary-level Mendelian randomisation analysis between plasma ACE and 48 vascular disease phenotypes.

[See the Excel File]

Table S18: Cis-locus MR analysis for plasma ACE and 48 vascular disease phenotypes.

[See the Excel File]

Table S19: Cis-SNP MR analysis for plasma ACE and ACE2 to cardiovascular disease phenotypes.

[See the Excel File]

Table S20: Cis-SNP MR analysis for plasma ACE and ACE2 to cardiovascular disease and blood pressure phenotypes.

[See the Excel File]

Table S21: Author list of the IMI-DIRECT Consortium.

[See the Excel File]

5 SUPPLEMENTAL INFORMATION

Cohort-specific acknowledgements

ARISTOTLE This work was supported by the Swedish Foundation for Strategic Research. The ARISTOTLE trial (ClinicalTrials.gov Identifier: NCT00412984) was funded by Bristol-Myers Squibb Co., Princeton, NJ, USA, and Pfizer Inc., New York, NY, USA. The computations were enabled by resources provided by the Swedish National Infrastructure for Computing (SNIC) at Uppsala Multidisciplinary Center for Advanced Computational Science (UPPMAX) partially funded by the Swedish Research Council through grant agreement no. 2018-05973 (project sens2017573).

HELIC MANOLIS & HELIC POMAK This work was funded by the Wellcome Trust (098051) and the European Research Council (ERC-2011-StG 280559-SEPI). The MANOLIS cohort is named in honour of Manolis Giannakakis, 1978-2010. We thank the residents of the Mylopotamos villages, and of the Pomak villages, for taking part. The HELIC study has been supported by many individuals who have contributed to sample collection (including A. Athanasiadis, O. Balafouti, C. Batzaki, G. Daskalakis, E. Emmanouil, C. Giannakaki, M. Giannakopoulou, A. Kaparou, V. Kariakli, S. Koinaki, D. Kokori, M. Konidari, H. Koundouraki, D. Koutoukidis, V. Mamakou, E. Mamalaki, E. Mpamiaki, M. Tsoukana, D. Tzakou, K. Vosdogianni, N. Xenaki, E. Zengini), data entry (T. Antonos, D. Papagrigoriou, B. Spiliopoulou), sample logistics (S. Edkins, E. Gray), genotyping (R. Andrews, H. Blackburn, D. Simpkin, S. Whitehead), research administration (A. Kolb-Kokocinski, S. Smee, D. Walker) and informatics (M. Pollard, J. Randall)."

EGCUT EGCUT work was supported by the European Regional Development Fund and the programme Mobilitas Pluss (MOBTP108), SP1GI18045T, No.2014-2020.4.01.15-0012 GENTRANSMED and 2014-2020.4.01.16-0125 This study was also funded by EU H2020 grant 692145, Estonian Research Council Grant PUT1660. Data analyses with Estonian datasets were carried out in part in the High-Performance Computing Center of University of Tartu.

VIS The VIS study was funded by grants from the Medical Research Council (UK), European Commission Framework 6 project EUROSPAN (Contract No. LSHG-CT-2006-018947), the Republic of Croatia Ministry of Science, Education and Sports research grants (216-1080315-0302), the Croatian National Center of Research Excellence in Personalized Healthcare (grant number KK.01.1.1.01.0010), and the Center of Competence in Molecular Diagnostics (KK.01.2.2.03.0006).

WHI The WHI program is funded by the National Heart, Lung, and Blood Institute, National Institutes of Health, U.S. Department of Health and Human Services through contracts HHSN268201600018C, HHSN268201600001C, HHSN268201600002C, HHSN268201600003C, and HHSN268201600004C. We thank all the investigators who have con-

tributed to WHI science. Please visit: https://s3-us-west-2.amazonaws.com/www-whi-org/wp-content/uploads/WHI-Investigator-Long-List.pdf

FENLAND The Fenland Study (10.22025/2017.10.101.00001) is funded by the Medical Research Council (MC_UU_12015/1). We further acknowledge support for genomics and metabolomics from the Medical Research Council (MC_PC_13046). We are grateful to all the volunteers and to the General Practitioners and practice staff for assistance with recruitment. We thank the Fenland Study Investigators, Fenland Study Co-ordination team and the Epidemiology Field, Data and Laboratory teams.

GenOMICC J.K.B. gratefully acknowledges funding support from a Wellcome Trust Senior Research Fellowship (223164/Z/21/Z), UKRI grants MC_PC_20004, MC_PC_19025, MC_PC_1905, MRNO2995X/1, Sepsis Research (Fiona Elizabeth Agnew Trust), a BBSRC Institute Strategic Programme Grant to the Roslin Institute (BB/P013732/1, BB/P013759/1), and the UK Intensive Care Society.

NSPHS The Northern Swedish Population Health Study (NSPHS) has been funded by the Swedish Medical Research Council (K2007-66X-20270-01-3, 2011-5252, 2012-2884 and 2011-2354), and the Foundation for Strategic Research (SSF). NSPHS as part of European Special Populations Research Network (EUROSPAN) was also supported by the European Commission FP6 STRP (01947, LSHG-CT-2006-01947). Whole-genome sequencing was funded by SciLifeLab. Computations were performed on resources provided by SNIC through Uppsala Multidisciplinary Center for Advanced Computational Science (UPPMAX) under project sens2016007.

DIRECT The DIRECT study has received support from the Innovative Medicines Initiative Joint Undertaking under grant agreement No. 115317 (DIRECT), resources of which are composed of financial contribution from the European Union's Seventh Framework Programme (FP7/2007-2013) and EFPIA companies' in kind contribution.

INTERVAL Participants in the INTERVAL randomised controlled trial were recruited with the active collaboration of NHS Blood and Transplant England (www.nhsbt.nhs.uk), which has supported field work and other elements of the trial. DNA extraction and genotyping was co-funded by the National Institute for Health Research (NIHR), the NIHR BioResource (http://bioresource.nihr.ac.uk) and the NIHR Cambridge Biomedical Research Centre (BRC) [*]. The academic coordinating centre for INTERVAL was supported by core funding from: NIHR Blood and Transplant Research Unit in Donor Health and Genomics (NIHR BTRU-2014-10024), UK Medical Research Council (MR/L003120/1), British Heart Foundation (SP/09/002; RG/13/13/30194; RG/18/13/33946) and the NIHR Cambridge BRC [*]. A complete list of the investigators and contributors to the INTERVAL trial is provided in reference 57[57]. The academic coordinating centre would like to thank blood donor centre staff and blood donors for participating in the INTERVAL trial.

This work was supported by Health Data Research UK, which is funded by the UK Medical Research Council, Engineering and Physical Sciences Research Council, Economic and Social Research Council, Department of Health and Social Care (England), Chief Scientist Office of the Scottish Government Health and Social Care Directorates, Health and Social Care Research and Development Division (Welsh Government), Public Health Agency (Northern Ireland), British Heart Foundation and Wellcome.

*The views expressed are those of the author(s) and not necessarily those of the NIHR or the Department of Health and Social Care.

IMI-DIRECT Consortium Author List

[See Table S21]

GenOMICC Consortium Author List

[See next page]

GenOMICC co-investigators

Sara Clohisey[1], Peter Horby[21], Johnny Millar[1], Julian Knight[14], Hugh Montgomery[29], David Maslove[25], Lowell Ling[26], Alistair Nichol[22], Charlotte Summers[15], Tim Walsh[11], Charles Hinds[20], Malcolm G. Semple[18,38], Peter J.M. Openshaw[36,37], Manu Shankar-Hari[16], Antonia Ho[19], Danny McAuley[27,28], Chris Ponting[2], Kathy Rowan[7], J. Kenneth Baillie[1,2,11].

Central management and laboratory team

Fiona Griffiths[1], Wilna Oosthuyzen[1], Jen Meikle[1], Paul Finernan[1], James Furniss[1], Ellie Mcmaster[1], Andy Law[1], Sara Clohisey[1], J. Kenneth Baillie[1,11], Trevor Paterson[1], Tony Wackett[1], Ruth Armstrong[1], Lee Murphy[6], Angie Fawkes[6], Richard Clark[6], Audrey Coutts[6], Lorna Donnelly[6], Tammy Gilchrist[6], Katarzyna Hafezi[6], Louise Macgillivray[6], Alan Maclean[6], Sarah McCafferty[6], Kirstie Morrice[6], Jane Weaver[1], Ceilia Boz[1], Ailsa Golightly[1], Mari Ward[1], Hanning Mal[1], Helen Szoor-McElhinney[1], Adam Brown[1], Ross Hendry[1], Andrew Stenhouse[1], Louise Cullum[1], Dawn Law[1], Sarah Law[1], Rachel Law[1], Max Head Fourman[1], Maaike Swets[1], Nicky Day[1], Filip Taneski[1], Esther Duncan[1], Marie Zechner[1], Nicholas Parkinson[1].

Data analysis team

Erola Pairo-Castineira[1,2], Sara Clohisey[1], Lucija Klaric[2], Andrew D. Bretherick[2], Konrad Rawlik[1], Dorota Pasko[3], Susan Walker[3], Nick Parkinson[1], Max Head Fourman[1], Clark D Russell[1,4], James Furniss[1], Anne Richmond[2], Elvina Gountouna[5], David Harrison[7], Bo Wang[1], Yang Wu[8], Alison Meynert[2], Athanasios Kousathanas[3], Loukas Moutsianas[3], Zhijian Yang[9], Ranran Zhai[9], Chenqing Zheng[9], Graeme Grimes[2], Jonathan Millar[1], Barbara Shih[1], Marie Zechner[1], Jian Yang[12,13], Xia Shen[9,33,34], Chris P. Ponting[2], Albert Tenesa[1,2,33], Kathy Rowan[7], Andrew Law[1], Veronique Vitart[2], James F. Wilson[2,33], J. Kenneth Baillie[1,2,11].

Barts Health NHS Trust, London, UK

D Collier[39], S Wood[39], A Zak[39], C Borra[39], M Matharu[39], P May[39], Z Alldis[39], O Mitchelmore[39], R Bowles[39], A Easthorpe[39], F Bibi[39], I Lancoma-Malcolm[39], J Gurasashvili[39], J Pheby[39], J Shiel[39], M Bolton[39], M Patel[39], M Taylor[39], O Zongo[39], P Ebano[39], P Harding[39], R Astin-Chamberlain[39], Y Choudhury[39], A Cox[39], D Kallon[39], M Burton[39], R Hall[39], S Blowes[39], Z Prime[39], J Biddle[39], O Prysyazhna[39], T Newman[39], C Tierney[39], J Kassam[39].

Guys and St Thomas' Hospital, London, UK

M Shankar-Hari[40], M Ostermann[40], S Campos[40], A Bociek[40], R Lim[40], N Grau[40], T O Jones[40], C Whitton[40], M Marotti[40], G Arbane[40].

James Cook University Hospital, Middlesburgh, UK

S. Bonner[41], K Hugill[41], J Reid[41].

The Royal Liverpool University Hospital, Liverpool, UK

I Welters[42], V Waugh[42], K Williams[42], D Shaw[42], J Fernandez Roman[42], M Lopez Martinez[42], E Johnson[42], A Waite[42], B Johnson[42], O Hamilton[42], S Mulla[42].

King's College Hospital, London, UK

M McPhail[43], J Smith[43].

Royal Infirmary of Edinburgh, Edinburgh, UK

J K Baillie[1,2,11], L Barclay[44], D Hope[44], C McCulloch[44], L McQuillan[44], S Clark[44], J Singleton[44], K Priestley[44], N Rea[44], M Callaghan[44], R Campbell[44], G Andrew[44], L Marshall[44].

John Radcliffe Hospital, Oxford, UK

S McKechnie[45], P Hutton[45], A Bashyal[45], N Davidson[45].

Addenbrooke's Hospital, Cambridge, UK

C Summers[46], P Polgarova[46], K Stroud[46], N Pathan[46], K Elston[46], S Agrawal[46].

Morriston Hospital, Swansea, UK

C Battle[47], L Newey[47], T Rees[47], R Harford[47], E Brinkworth[47], M Williams[47], C Murphy[47].

Ashford and St Peter's Hospital, Surrey, UK

I White[48], M Croft[48].

Royal Stoke University Hospital, Staffordshire, UK

N Bandla[49], M Gellamucho[49], J Tomlinson[49], H Turner[49], M Davies[49], A Quinn[49], I Hussain[49], C Thompson[49], H Parker[49], R Bradley[49], R Griffiths[49].

Queen Elizabeth Hospital, Birmingham, UK

J. Scriven[50], J Gill[50].

Glasgow Royal Infirmary, Glasgow, UK

A Puxty[51], S Cathcart[51], D Salutous[51], L Turner[51], K Duffy[51], K Puxty[51].

Kingston Hospital, Surrey, UK

A Joseph[52], R Herdman-Grant[52], R Simms[52], A Swain[52], A Naranjo[52], R Crowe[52], K Sollesta[52], A Loveridge[52], D Baptista[52], E Morino[52].

The Tunbridge Wells Hospital and Maidstone Hospital, Kent, UK

M Davey[53], D Golden[53], J Jones[53].

North Middlesex University Hospital NHS trust, London, UK

J Moreno Cuesta[54], A Haldeos[54], D Bakthavatsalam[54], R Vincent[54], M Elhassan[54], K Xavier[54], A Ganesan[54], D Purohit M Abdelrazik[54].

Bradford Royal Infirmary, Bradford, UK

J Morgan[55], L Akeroyd[55], S Bano[55], D Warren[55], M Bromley[55], K Sellick[55], L Gurr[55], B Wilkinson[55], V Nagarajan[55], P Szedlak[55].

Blackpool Victoria Hospital, Blackpool, UK

J Cupitt[56], E Stoddard[56], L Benham[56], S Preston[56], N Slawson[56], Z Bradshaw[56], J brown[56], M Caswell[56], S Melling[56].

Countess of Chester Hospital, Chester, UK

P Bamford[57], M Faulkner[57], K Cawley[57], H Jeffrey[57], E London[57], H Sainsbury[57], I Nagra[57], F Nasir[57], Ce Dunmore[57], R Jones[57], A Abraheem[57], M Al-Moasseb[57], R Girach[57].

Wythenshawe Hospital, Manchester, UK

C Brantwood[58], P Alexander[58], J Bradley-Potts[58], S Allen[58], T Felton[58].

St George's Hospital, London, UK

S Manna[59], S Farnell-Ward[59], S Leaver[59], J Queiroz[59], E Maccacari[59], D Dawson[59], C Castro Delgado[59], R Pepermans Saluzzio[59], O Ezeobu[59], L Ding[59], C Sicat[59], R Kanu[59], G Durrant[59], J Texeira[59], A Harrison[59], T Samakomva[59].

Good Hope Hospital, Birmingham, UK

J Scriven[60], H Willis[60], B Hopkins[60], L Thrasyvoulou[60].

Stepping Hill Hospital, Stockport, UK

M Jackson[61], A Zaki[61], C Tibke[61], S Bennett[61], W Woodyatt[61], A Kent[61], E Goodwin[61].

Manchester Royal Infirmary, Manchester, UK

C Brandwood[62], R Clark[62], L Smith[62].

Royal Alexandra Hospital, Paisley, UK

K Rooney[63], N Thomson[63], N Rodden[63], E Hughes[63], D McGlynn[63], C Clark[63], P Clark[63], L Abel[63], R Sundaram[63], L Gemmell[63], M Brett[63], J Hornsby[63], P MacGoey[63], R Price[63], B Digby[63], P O'Neil[63], P McConnell[63], P Henderson[63].

Queen Elizabeth University Hospital, Glasgow, UK

S Henderson[64], M Sim[64], S Kennedy-Hay[64], C McParland[64], L Rooney[64], N Baxter[64].

Queen Alexandra Hospital, Portsmouth, UK

D Pogson[65], S Rose[65], Z Daly[65], L Brimfield[65].

BHRUT (Barking Havering) - Queens Hospital and King George Hospital, Essex, UK

M K Phull[66], M Hussain[66], T Pogreban[66], L Rosaroso[66], E Salciute L Grauslyte[66].

University College Hospital, London, UK

D Brealey[67], E Wraith[67], N MacCallum[67], G Bercades[67], I Hass[67], D Smyth[67], A Reyes[67], G Martir[67].

Royal Victoria Infirmary, Newcastle Upon Tyne, UK

I D Clement[68], K Webster[68], C Hays[68], A Gulati[68].

Western Sussex Hospitals, West Sussex, UK

L Hodgson[69], M Margarson[69], R Gomez[69], Y Baird[69], Y Thirlwall[69], L Folkes[69], A Butler[69], E Meadows[69], S Moore[69], D Raynard[69], H Fox[69], L Riddles[69], K King[69], S Kimber[69], G Hobden[69], A McCarthy[69], V Cannons[69], I Balagosa[69], I Chadbourn[69], A Gardner[69].

Salford Royal Hospital, Manchester, UK

D Horner[70], D McLaughlanv[70], B Charles[70], N Proudfoot[70], T Marsden[70], L Mc Morrow[70], B Blackledge[70], J Pendlebury[70], A Harvey[70], E Apetri[70], C Basikolo[70], L Catlow[70], R Doonan[70], K Knowles[70], S Lee[70], D Lomas[70], C Lyons[70], J Perez[70], M Poulaka[70], M Slaughter[70], K Slevin[70], M Taylor[70], V Thomas[70], D Walker[70], J Harris[70].

The Royal Oldham Hospital, Manchester, UK

A Drummond[71], R Tully[71], J Dearden[71], J Philbin[71], S Munt[71], C Rishton[71], G O'Connor[71], M Mulcahy[71], E Dobson[71], J Cuttler[71], M Edward[71].

Pinderfields General Hospital, Wakefield, UK

A Rose[72], B Sloan[72], S Buckley[72], H Brooke[72], E Smithson[72], R Charlesworth[72], R Sandu[72], M Thirumaran[72], V Wagstaff[72], J Cebrian Suarez[72].

Basildon Hospital, Basildon, UK

A Kaliappan[73], M Vertue[73], A Nicholson[73], J Riches[73], A Solesbury[73], L Kittridge[73], M Forsey[73], G Maloney[73].

University Hospital of Wales, Cardiff, UK

J Cole[74], M Davies[74], R Davies[74], H Hill[74], E Thomas[74], A Williams[74], D Duffin[74], B Player[74].

Broomfield Hospital, Chelmsford, UK

J Radhakrishnan[75], S Gibson[75], A Lyle[75], F McNeela[75].

Royal Brompton Hospital, London, UK

B Patel[76], M Gummadi[76], G Sloane[76], N Dormand[76], S Salmi[76], Z Farzad[76], D Cristiano[76], K Liyanage[76], V Thwaites[76], M Varghese[76].

Nottingham University Hospital, Nottingham, UK

M Meredith[77].

Royal Hallamshire Hospital and Northern General Hospital, Sheffield, UK

G Mills[78], J Willson[78], K Harrington[78], B Lenagh[78], K Cawthron[78], S Masuko[78], A Raithatha[78], K Bauchmuller[78], N Ahmad[78], J Barker[78], Y Jackson[78], F Kibutu[78], S Bird[78].

Royal Hampshire County Hospital, Hampshire, UK

G Watson[79], J Martin[79], E Bevan[79], C Wrey Brown[79], D Trodd[79].

Queens Hospital Burton, Burton-On-Trent, UK

K English[80], G Bell[80], L Wilcox[80], A Katary[80].

New Cross Hospital, Wolverhampton, UK

S Gopal[81], V Lake[81], N Harris[81], S Metherell[81], E Radford[81].

Heartlands Hospital, Birmingham, UK

J Scriven[82], F Moore[82], H Bancroft[82], J Daglish[82], M Sangombe[82], M Carmody[82], J Rhodes[82], M Bellamy[82].

Walsall Manor Hospital, Walsall, UK

A Garg[83], A Kuravi[83], E Virgilio[83], P Ranga[83], J Butler[83], L Botfield[83], C Dexter[83], J Fletcher[83].

Stoke Mandeville Hospital, Buckinghamshire, UK

P Shanmugasundaram[84], G Hambrook[84], I Burn[84], K Manso[84], D Thornton[84], J Tebbutt[84], R Penn[84].

Sandwell General Hospital, Birmingham, UK

J Hulme[85], S Hussain[85], Z Maqsood[85], S Joseph[85], J Colley[85], A Hayes[85], C Ahmed[85], R Haque[85], S Clamp[85], R Kumar[85], M Purewal[85], B Baines[85].

Royal Berkshire NHS Foundation Trust, Berkshire, UK

M Frise[86], N Jacques[86], H Coles[86], J Caterson[86], S Gurung Rai[86], M Brunton[86], E Tilney[86], L Keating[86], A Walden[86].

Charing Cross Hospital, St Mary's Hospital and Hammersmith Hospital, London, UK

D Antcliffe[87], A Gordon[87], M Templeton[87], R Rojo[87], D Banach[87], S Sousa Arias[87], Z Fernandez[87], P Coghlan[87].

Dumfries and Galloway Royal Infirmary, Dumfries, UK

D Williams[88], C Jardine[88].

Bristol Royal Infirmary, Bristol, UK

J Bewley[89], K Sweet[89], L Grimmer[89], R Johnson[89], Z Garland[89], B Gumbrill[89].

Royal Sussex County Hospital, Brighton, UK

C Phillips[90], L Ortiz-Ruiz de Gordoa[90], E Peasgood[90].

Whiston Hospital, Prescot, UK

A Tridente[91], K Shuker S Greer[91].

Royal Glamorgan Hospital, Cardiff, UK

C Lynch[92], C Pothecary[92], L Roche[92], B Deacon[92], K Turner[92], J Singh[92], G Sera Howe[92].

King's Mill Hospital, Nottingham, UK

P Paul[93], M Gill[93], I Wynter[93], V Ratnam[93], S Shelton[93].

Fairfield General Hospital, Bury, UK

J Naisbitt[94], J Melville[94].

Western General Hospital, Edinburgh, UK

R Baruah[95], S Morrison[95].

Northwick Park Hospital, London, UK

A McGregor[96], V Parris[96], M Mpelembue[96], S Srikaran[96], C Dennis[96], A Sukha[96].

Royal Preston Hospital, Preston, UK

A Williams[97], M Verlande[97].

Royal Derby Hospital, Derby, UK

K Holding[98], K Riches[98], C Downes[98], C Swan[98].

Sunderland Royal Hospital, Sunderland, UK

A Rostron[99], A Roy[99], L Woods[99], S Cornell[99], F Wakinshaw[99].

Royal Surrey County Hospital, Guildford, UK

B Creagh-Brown[100], H Blackman[100], A Salberg[100], E Smith[100], S Donlon[100], S Mtuwa[100], N Michalak-Glinska[100], S Stone[100], C Beazley[100], V Pristopan[100].

Derriford Hospital, Plymouth, UK

N Nikitas[101], L Lankester[101], C Wells[101].

Croydon University Hospital, Croydon, UK

A S Raj[102], K Fletcher[102], R Khade[102], G Tsinaslanidis[102].

Victoria Hospital, Kirkcaldy, UK

M McMahon[103], S Fowler[103], A McGregor[103], T Coventry[103].

Milton Keynes University Hospital, Milton Keynes, UK

R Stewart[104], L Wren[104], E Mwaura[104], L Mew[104], A Rose[104], D Scaletta[104], F Williams[104].

Barnsley Hospital, Barnsley, UK

K Inweregbu[105], A Nicholson[105], N Lancaster[105], M Cunningham[105], A Daniels[105], L Harrison[105], S Hope[105], S Jones[105], A Crew[105], G Wray[105], J Matthews[105], R Crawley[105].

York Hospital, York, UK

J Carter[106], I Birkinshaw[106], J Ingham[106], Z Scott[106], K Howard[106], R Joy[106], S Roche[106].

University Hospital of North Tees, Stockton on Tees, UK

M Clark[107], S Purvis[107].

University Hospital Wishaw, Wishaw, UK

A Morrison[108], D Strachan[108], M Taylor[108], S Clements[108], K Black[108].

Whittington Hospital, London, UK

C Parmar[109], A Altabaibeh[109], K Simpson[109], L Mostoles[109], K Gilbert[109], L Ma[109], A Alvaro[109].

Southmead Hospital, Bristol, UK

M Thomas[110], B Faulkner[110], R Worner[110], K Hayes[110], E Gendall[110], H Blakemore[110], B Borislavova[110], E Goff[110].

The Royal Papworth Hospital, Cambridge, UK

A Vuylsteke[111], L Mwaura[111], J Zamikula[111], L Garner[111], A Mitchell[111], S Mepham[111], L Cagova[111], A Fofano[111], H Holcombe[111], K Praman[111].

Royal Gwent Hospital, Newport, UK

T Szakmany[112], A E Heron[112], S Cherian[112], S Cutler[112], A Roynon-Reed[112].

Norfolk and Norwich University hospital (NNUH), Norwich, UK

G Randell[113], K Convery[113], K Stammers D Fottrell-Gould[113], L Hudig[113], J Keshet-price[113].

Great Ormond St Hospital and UCL Great Ormond St Institute of Child Health NIHR Biomedical Research Centre, London, UK

M Peters[114], L O'Neill[114], S Ray[114], H Belfield[114], T McHugh[114], G Jones[114], O Akinkugbe[114], A Tomas[114], E Abaleke[114], E Beech[114], H Meghari[114], S Yussuf[114], A Bamford[114].

Airedale General Hospital, Keighley, UK

B Hairsine[115], E Dooks[115], F Farquhar[115], S Packham[115], H Bates[115], C McParland[115], L Armstrong[115].

Aberdeen Royal Infirmary, Aberdeen, UK

C Kaye[116], A Allan[116], J Medhora[116], J Liew[116], A Botello[116], F Anderson[116].

Southampton General Hospital, Southampton, UK

R Cusack[117], H Golding[117], K Prager[117], T Williams[117], S Leggett[117], K Golder[117], M Male[117], O Jones[117], K Criste[117], M Marani[117].

Russell's Hall Hospital, Dudley, UK

Dr. Anumakonda[118], V Amin[118], K Karthik[118], R Kausar[118], E Anastasescu[118], K Reid[118], Ms. Jacqui[118].

Rotherham General Hospital, Rotherham, UK

A Hormis[119], R Walker[119], D Collier[119].

North Manchester General Hospital, Manchester, UK

T Duncan[120], A Uriel[120], A Ustianowski[120], H T-Michael[120], M Bruce[120], K Connolly[120], K Smith[120].

Basingstoke and North Hampshire Hospital, Basingstoke, UK

R Partridge[121], D Griffin[121], M McDonald[121], N Muchenje[121].

Royal Free Hospital, London, UK

D Martin[122], H Filipe[122], C Eastgate[122], C Jackson[122].

Hull Royal Infirmary, Hull, UK

A Gratrix[123], L Foster[123], V Martinson[123], E Stones[123], Caroline Abernathy[123], P Parkinson[123].

Harefield Hospital, London, UK

A Reed[124], C Prendergast[124], P Rogers[124], M Woodruff[124], R Shokkar[124], S Kaul[124], A Barron[124], C Collins[124].

Chesterfield Royal Hospital Foundation Trust, Chesterfield, UK

S Beavis[125], A Whileman[125], K Dale[125], J Hawes[125], K Pritchard[125], R Gascoyne[125], L Stevenson[125].

Barnet Hospital, London, UK

R Jha[126], L Lim[126], V Krishnamurthy[126].

Aintree University Hospital, Liverpool, UK

R Parker[127], I Turner-Bone[127], L Wilding[127], A Reddy[127].

St James's University Hospital and Leeds General Infirmary, Leeds, UK

S Whiteley[128], E Wilby[128], C Howcroft[128], A Aspinwall[128], S Charlton[128], B Ogg[128].

Glan Clwyd Hospital, Bodelwyddan, UK

D Menzies[129], R Pugh[129], E Allan[129], R Lean[129], F Davies[129], J Easton[129], X Qiu[129], S Kumar[129], K Darlington[129].

University Hospital Crosshouse, Kilmarnock, UK

G Houston[130], P O'Brien[130], T Geary[130], J Allan[130], A Meikle[130].

Royal Bolton Hospital, Bolton, UK

G Hughes[131], M Balasubramaniam[131], S Latham[131], E McKenna[131], R Flanagan[131].

Princess of Wales Hospital, Llantrisant, UK

S Sathe[132], E Davies[132], L Roche[132].

Pilgrim Hospital, Lincoln, UK

M Chablani[133], A Kirkby[133], K Netherton[133], S Archer[133].

Northumbria Healthcare NHS Foundation Trust, North Shields, UK

B Yates[134], C Ashbrook-Raby[134].

Ninewells Hospital, Dundee, UK

S Cole[135], M Casey[135], L Cabrelli[135], S Chapman[135], M Casey[135], P Austin[135], A Hutcheon[135], C Whyte[135], C Almaden- Boyle[135].

Lister Hospital, Stevenage, UK

N Pattison[136], C Cruz[136].

Bedford Hospital, Bedford, UK

A Vochin[137], H Kent[137], A Thomas[137], S Murdoch[137], B David[137], M Penacerrada[137], G Lubimbi[137], V Bastion[137], R Wulandari[137], J Valentine[137], D Clarke[137].

Royal United Hospital, Bath, UK

A Serrano-Ruiz[138], S Hierons[138], L Ramos[138], C Demetriou[138], S Mitchard[138], K White[138].

Royal Bournemouth Hospital, Bournemouth, UK

N White[139], S Pitts[139], D Branney[139], J Frankham[139].

The Great Western Hospital, Swindon, UK

M Watters[140], H Langton[140], R Prout[140].

Watford General Hospital, Watford, UK

V Page[141], T Varghes[141].

University Hospital North Durham, Darlington, UK

A Cowton[142], A Kay[142], K Potts[142], M Birt[142], M Kent[142], A Wilkinson[142].

Tameside General Hospital, Ashton Under Lyne, UK

E Jude[143], V Turner[143], H Savill[143], J McCormick[143], M Clark[143], M Coulding[143], S Siddiqui[143], O Mercer[143], H Rehman[143], D Potla[143].

Princess Royal Hospital, Telford and Royal Shrewsbury Hospital, Shrewsbury, UK

N Capps[144], D Donaldson[144], J Jones[144], H Button[144], T Martin[144], K Hard[144], A Agasou[144], L Tonks[144], T Arden[144], P Boyle[144], M Carnahan[144], J Strickley[144], C Adams[144], D Childs[144], R Rikunenko[144], M Leigh[144], M Breekes[144], R Wilcox[144], A Bowes[144], H Tiveran[144], F Hurford[144], J Summers[144], A Carter[144], Y Hussain[144], L Ting[144], A Javaid[144], N Motherwell[144], H Moore[144], H Millward[144], S Jose[144], N Schunki[144], A Noakes[144], C Clulow[144].

Arrowe Park Hospital, Wirral, UK

G Sadera[145], R Jacob[145], C Jones[145].

The Queen Elizabeth Hospital, King's Lynn, UK

M Blunt[146], Z Coton[146], H Curgenven[146], S Mohamed Ally[146], K Beaumont[146], M Elsaadany[146], K Fernandes[146], I Ali Mohamed Ali[146], H Rangarajan[146], V Sarathy[146], S Selvanayagam[146], D Vedage[146], M White[146].

Royal Blackburn Teaching Hospital, Blackburn, UK

M Smith[147], N Truman[147], S Chukkambotla[147], S Keith[147], J Cockerill-Taylor[147], J Ryan-Smith[147], R Bolton[147], P Springle[147], J Dykes[147], J Thomas[147], M Khan[147], M T Hijazi[147], E Massey[147], G Croston[147].

Poole Hospital, Poole, UK

H Reschreite r[148], J Camsooksai[148], S Patch[148], S Jenkins[148], C Humphrey[148], B Wadams[148], J Camsooksai[148].

Medway Maritime Hospital, Gillingham, UK

N Bhatia[149], M Msiska[149], O Adanini[149].

Warwick Hospital, Warwick, UK

B Attwood[150], P Parsons[150].

The Royal Marsden Hospital, London, UK

K Tatham[151], S Jhanji[151], E Black[151], A Dela Rosa[151], R Howle[151], B Thomas[151], T Bemand[151], R Raobaikady[151].

The Princess Alexandra Hospital, Harlow, UK

R Saha[152], N Staines[152], A Daniel[152], J Finn[152].

Musgrove Park Hospital, Taunton, UK

J Hutter[153], P Doble[153], C Shovelton[153], C Pawley[153].

George Eliot Hospital NHS Trust, Nuneaton, UK

T Kannan[154], M Hill[154].

East Surrey Hospital, Redhill, UK

E Combes[155], S Monnery[155], T Joefield[155].

West Middlesex Hospital, Isleworth, UK

M Popescu[156], M Thankachen[156], M Oblak[156].

Warrington General Hospital, Warrington, UK

J Little[157], S McIvor[157], A Brady[157], H Whittle[157], H Prady[157], R Chan[157].

Southport and Formby District General Hospital, Ormskirk, UK

A Ahmed[158], A Morris[158].

Royal Devon and Exeter Hospital, Exeter, UK

C Gibson[159], E Gordon[159], S Keenan[159], H Quinn[159], S Benyon[159], S Marriott[159], L Zitter[159], L Park[159], K Baines[159].

Macclesfield District General Hospital, Macclesfield, UK

M Lyons[160], M Holland[160], N Keenan[160], M Young[160].

Borders General Hospital, Melrose, UK

S Garrioch[161], J Dawson[161], M Tolson[161].

Birmingham Children's Hospital, Birmingham, UK

B Scholefield[162], R Bi[162].

William Harvey Hospital, Ashford, UK

N Richardson[163], N Schumacher[163], T Cosier[163], G Millen[163].

Royal Lancaster Infirmary, Lancaster, UK

A Higham[164], K Simpson[164].

Queen Elizabeth the Queen Mother Hospital, Margate, UK

S Turki[165], L Allen[165], N Crisp[165], T Hazleton[165], A Knight[165], J Deery[165], C Price[165], S Turney[165], S Tilbey[165], E Beranova[165].

Liverpool Heart and Chest Hospital, Liverpool, UK

D Wright[166], L Georg[166], S Twiss[166].

Darlington Memorial Hospital, Darlington, UK

A Cowton[167], S Wadd[167], K Postlethwaite[167].

Southend University Hospital, Westcliff-on-Sea, UK

P Gondo[168], B Masunda[168], A Kayani[168], B Hadebe[168].

Raigmore Hospital, Inverness, UK

J Whiteside[169], R Campbell[169], N Clarke[169].

Salisbury District Hospital, Salisbury, UK

P Donnison[170], F Trim[170], I Leadbitter[170].

Peterborough City Hospital, Peterborough, UK

D Butcher[171], S O'Sullivan[171].

Ipswich Hospital, Ipswich, UK

B Purewal[172], B Purewal[172], S Bell[172], V Rivers'[172].

Hereford County Hospital, Hereford, UK

R O'Leary[173], J Birch[173], E Collins[173], S Anderson[173], K Hammerton[173], E Andrews[173].

Furness General Hospital, Barrow-in-Furness, UK

A Higham[174], K Burns[174].

Forth Valley Royal Hospital, Falkirk, UK

I Edmond[175], D Salutous[175], A Todd[175], J Donnachie[175], P Turner[175], L Prentice[175], L Symon[175], N Runciman[175], F Auld[175].

Torbay Hospital, Torquay, UK

M Halkes[176], P Mercer[176], L Thornton[176].

St Mary's Hospital, Newport, UK

G Debreceni[177], J Wilkins[177], A Brown[177], V Crickmore[177].

Royal Manchester Children's Hospital, Manchester, UK

G Subramanian[178], R Marshall[178], C Jennings[178], M Latif[178], L Bunni[178].

Royal Cornwall Hospital, Truro, UK

M Spivey[179], S Bean[179], K Burt[179].

Queen Elizabeth Hospital Gateshead, Gateshead, UK

V Linnett[180], J Ritzema[180], A Sanderson[180], W McCormick[180], M Bokhari[180].

Kent & Canterbury Hospital, Canterbury, UK

R Kapoor[181], D Loader[181].

James Paget University Hospital NHS Trust, Great Yarmouth, UK

A Ayers[182], W Harrison[182], J North[182].

Darent Valley Hospital, Dartford, UK

Z Belagodu[183], R Parasomthy[183], O Olufuwa[183], A Gherman[183], B Fuller[183], C Stuart[183].

The Alexandra Hospital, Redditch and Worcester Royal Hospital, Worcester, UK

O Kelsall[184], C Davis[184], L Wild[184], H Wood[184], J Thrush[184], A Durie[184], K Austin'[184], K Archer[184], P Anderson[184], C Vigurs[184].

Ysbyty Gwynedd, Bangor, UK

C Thorpe[185], A Thomas[185], E Knights[185], N Boyle[185], A Price[185].

Yeovil Hospital, Yeovil, UK

A Kubisz-Pudelko[186], D Wood[186], A Lewis[186], S Board[186], L Pippard[186], J Perry[186], K Beesley[186].

University Hospital Hairmyres, East Kilbride, UK

A Rattray[187], M Taylor[187], E Lee[187], L Lennon[187], K Douglas[187], D Bell[187], R Boyle[187], L Glass[187].

Scunthorpe General Hospital, Scunthorpe, UK

M Nauman Akhtar[188], K Dent[188], D Potoczna[188], S Pearson[188], E Horsley[188], S Spencer[188].

Princess Royal Hospital Brighton, West Sussex, UK

C Phillips[189], D Mullan[189], D Skinner[189], J Gaylard[189], L Ortiz-Ruizdegordoa[189].

Lincoln County Hospital, Lincoln, UK

R Barber[190], C Hewitt[190], A Hilldrith[190], S Shepardson[190], M Wills[190], K Jackson-Lawrence[190].

Homerton University Hospital, London, UK

A Gupta[191], A Easthope[191], E Timlick[191], C Gorman[191].

Glangwili General Hospital, Camarthen, UK

I Otaha[192], A Gales[192], S Coetzee[192], M Raj[192], M Peiu[192].

Ealing Hospital, Southall, UK

V Parris[193], S Quaid[193], E Watson[193].

Scarborough General Hospital, Scarborough, UK

K Elliott[194], J Mallinson[194], B Chandler[194], A Turnbull[194].

Royal Albert Edward Infirmary, Wigan, UK

A Quinn[195], C Finch[195], C Holl[195], J Cooper[195], A Evans[195].

Queen Elizabeth Hospital, Woolwich, London, UK

W Khaliq[196], A Collins[196], E Treus Gude[196].

North Devon District Hospital, Barnstaple, UK

N Love[197], L van Koutrik[197], J Hunt[197], D Kaye[197], E Fisher[197], A Brayne[197], V Tuckey[197], P Jackson[197], J Parkin[197].

National Hospital for Neurology and Neurosurgery, London, UK

D Brealey[198], E Raith[198], A Tariq[198], H Houlden[198], A Tucci[198], J Hardy[198], E Moncur[198].

Eastbourne District General Hospital, East Sussex, UK and Conquest Hospital, East Sussex, UK

J Highgate[199], A Cowley[199].

Diana Princess of Wales Hospital, Grimsby, UK

A Mitra[200], R Stead[200], T Behan[200], C Burnett[200], M Newton[200], E Heeney[200], R Pollard[200], J Hatton[200].

The Christie NHS Foundation Trust, Manchester, UK

A Patel[201], V Kasipandian[201], S Allibone[201], R M Genetu[201].

Prince Philip Hospital, Lianelli, UK

I Otahal[202], L O'Brien[202], Z Omar[202], E Perkins[202], K Davies[202].

Prince Charles Hospital, Merthyr Tydfil, UK

D Tetla[203], C Pothecary[203], B Deacon[203].

Golden Jubilee National Hospital, Clydebank, UK

B Shelley[204], V Irvine[204].

Dorset County Hospital, Dorchester, UK

S Williams[205], P Williams[205], J Birch[205], J Goodsell[205], R Tutton[205], L Bough[205], B Winter-Goodwin[205].

Calderdale Royal Hospital, Halifax, UK

R Kitson[206], J Pinnell[206], A Wilson[206], T Nortcliffe[206], T Wood[206], M Home[206], K Holdroyd[206], M Robinson[206], KHanson[206], R Shaw[206], J Greig[206], M Brady[206], A Haigh[206], L Matupe[206], M Usher[206], S Mellor[206], S Dale[206], L Gledhill[206], L Shaw[206], G Turner[206], D Kelly[206], B Anwar[206], H Riley[206], H Sturgeon[206], A Ali[206], L Thomis[206], D Melia[206], A Dance[206], K Hanson[206].

West Suffolk Hospital, Suffolk, UK

S Humphreys[207], I Frost[207], V Gopal[207], J Godden[207], A Holden[207], S Swann[207].

West Cumberland Hospital, Whitehaven, UK

T Smith[208], M Clapham[208], U Poultney[208], R Harper[208], P Rice[208].

University Hospital Lewisham, London, UK

W Khaliq[209], R Reece-Anthony[209], B Gurung[209].

St John's Hospital Livingston, Livingston, UK

S Moultrie[210], M Odam[210].

Sheffield Children's Hospital, Sheffield, UK

A Mayer[211], A Bellini[211], A Pickard[211], J Bryant[211], N Roe[211], J Sowter[211].

Hinchingbrooke Hospital, Huntingdon, UK

D Butcher[212], K Lang[212], J Taylor[212].

Glenfield Hospital, Leicester, UK

P Barry[213].

Bronglais General Hospital, Aberystwyth, UK

M Hobrok[214], H Tench[214], R Wolf-Roberts[214], H McGuinness[214], R Loosley[214].

Alder Hey Children's Hospital, Liverpool, UK

D Hawcutt[215], L Rad[215], L O'Malley[215], P Saunderson[215], G Seddon[215], T Anderson[215], N Rogers[215].

University Hospital Monklands, Airdrie, UK

J Ruddy[216], Margaret H[216], M Taylor[216], C Beith[216], A McAlpine[216], L Ferguson[216], P Grant[216], S MacFadyen[216], M McLaughlin[216], T Baird[216], S Rundell[216], L Glass[216], B Welsh[216], R Hamill[216], F Fisher[216].

Cumberland Infirmary, Carlisle, UK

T Smith[217], J Gregory[217], A Brown[217].

[1]Roslin Institute, University of Edinburgh, Easter Bush, Edinburgh, EH25 9RG, UK
[21]Centre for Tropical Medicine and Global Health, Nuffield Department of Medicine, University of Oxford, Old Road Campus, Roosevelt Drive, Oxford, OX3 7FZ, UK
[14]Wellcome Centre for Human Genetics, University of Oxford, Oxford, UK
[29]UCL Centre for Human Health and Performance, London, W1T 7HA, UK
[25]Department of Critical Care Medicine, Queen's University and Kingston Health Sciences Centre, Kingston, ON, Canada
[26]Department of Anaesthesia and Intensive Care, The Chinese University of Hong Kong, Prince of Wales Hospital, Hong Kong, China
[22]Clinical Research Centre at St Vincent's University Hospital, University College Dublin, Dublin, Ireland
[15]Department of Medicine, University of Cambridge, Cambridge, UK

[11]Intensive Care Unit, Royal Infirmary of Edinburgh, 54 Little France Drive, Edinburgh, EH16 5SA, UK
[20]William Harvey Research Institute, Barts and the London School of Medicine and Dentistry, Queen Mary University of London, London EC1M 6BQ, UK
[18]NIHR Health Protection Research Unit for Emerging and Zoonotic Infections, Institute of Infection, Veterinary and Ecological Sciences University of Liverpool, Liverpool, L69 7BE, UK
[38]Respiratory Medicine, Alder Hey Children's Hospital, Institute in The Park, University of Liverpool, Alder Hey Children's Hospital, Liverpool, UK
[36]National Heart and Lung Institute, Imperial College London, London, UK
[37]Imperial College Healthcare NHS Trust: London, London, UK
[16]Department of Intensive Care Medicine, Guy's and St. Thomas NHS Foundation Trust, London, UK
[19]MRC-University of Glasgow Centre for Virus Research, Institute of Infection, Immunity and Inflammation, College of Medical, Veterinary and Life Sciences, University of Glasgow, Glasgow, UK
[27]Wellcome-Wolfson Institute for Experimental Medicine, Queen's University Belfast, Belfast, Northern Ireland, UK
[28]Department of Intensive Care Medicine, Royal Victoria Hospital, Belfast, Northern Ireland, UK
[2]MRC Human Genetics Unit, Institute of Genetics and Molecular Medicine, University of Edinburgh, Western General Hospital, Crewe Road, Edinburgh, EH4 2XU, UK
[7]Intensive Care National Audit & Research Centre, London, UK
[6]Edinburgh Clinical Research Facility, Western General Hospital, University of Edinburgh, EH4 2XU, UK
[3]Genomics England, London, UK
[4]Centre for Inflammation Research, The Queen's Medical Research Institute, University of Edinburgh, 47 Little France Crescent, Edinburgh, UK
[5]Centre for Genomic and Experimental Medicine, Institute of Genetics and Molecular Medicine, University of Edinburgh, Western General Hospital, Crewe Road, Edinburgh, EH4 2XU, UK
[8]Institute for Molecular Bioscience, The University of Queensland, Brisbane, Australia
[9]Biostatistics Group, School of Life Sciences, Sun Yat-sen University, Guangzhou, China
[12]School of Life Sciences, Westlake University, Hangzhou, Zhejiang 310024, China
[13]Westlake Laboratory of Life Sciences and Biomedicine, Hangzhou, Zhejiang 310024, China
[33]Centre for Global Health Research, Usher Institute of Population Health Sciences and Informatics, Teviot Place, Edinburgh EH8 9AG, UK
[34]Department of Medical Epidemiology and Biostatistics, Karolinska Institutet, Stockholm, Sweden
[39]Barts Health NHS Trust, London, UK
[40]Guys and St Thomas' Hospital, London, UK
[41]James Cook University Hospital, Middlesburgh, UK
[42]The Royal Liverpool University Hospital, Liverpool, UK
[43]King's College Hospital, London, UK
[44]Royal Infirmary of Edinburgh, Edinburgh, UK

[45]John Radcliffe Hospital, Oxford, UK
[46]Addenbrooke's Hospital, Cambridge, UK
[47]Morriston Hospital, Swansea, UK
[48]Ashford and St Peter's Hospital, Surrey, UK
[49]Royal Stoke University Hospital, Staffordshire, UK
[50]Queen Elizabeth Hospital, Birmingham, UK
[51]Glasgow Royal Infirmary, Glasgow, UK
[52]Kingston Hospital, Surrey, UK
[53]The Tunbridge Wells Hospital and Maidstone Hospital, Kent, UK
[54]North Middlesex University Hospital NHS trust, London, UK
[55]Bradford Royal Infirmary, Bradford, UK
[56]Blackpool Victoria Hospital, Blackpool, UK
[57]Countess of Chester Hospital, Chester, UK
[58]Wythenshawe Hospital, Manchester, UK
[59]St George's Hospital, London, UK
[60]Good Hope Hospital, Birmingham, UK
[61]Stepping Hill Hospital, Stockport, UK
[62]Manchester Royal Infirmary, Manchester, UK
[63]Royal Alexandra Hospital, Paisley, UK
[64]Queen Elizabeth University Hospital, Glasgow, UK
[65]Queen Alexandra Hospital, Portsmouth, UK
[66]BHRUT (Barking Havering) - Queens Hospital and King George Hospital, Essex, UK
[67]University College Hospital, London, UK
[68]Royal Victoria Infirmary, Newcastle Upon Tyne, UK
[69]Western Sussex Hospitals, West Sussex, UK
[70]Salford Royal Hospital, Manchester, UK
[71]The Royal Oldham Hospital, Manchester, UK
[72]Pinderfields General Hospital, Wakefield, UK
[73]Basildon Hospital, Basildon, UK
[74]University Hospital of Wales, Cardiff, UK
[75]Broomfield Hospital, Chelmsford, UK
[76]Royal Brompton Hospital, London, UK
[77]Nottingham University Hospital, Nottingham, UK
[78]Royal Hallamshire Hospital and Northern General Hospital, Sheffield, UK
[79]Royal Hampshire County Hospital, Hampshire, UK
[80]Queens Hospital Burton, Burton-On-Trent, UK
[81]New Cross Hospital, Wolverhampton, UK
[82]Heartlands Hospital, Birmingham, UK
[83]Walsall Manor Hospital, Walsall, UK
[84]Stoke Mandeville Hospital, Buckinghamshire, UK
[85]Sandwell General Hospital, Birmingham, UK
[86]Royal Berkshire NHS Foundation Trust, Berkshire, UK
[87]Charing Cross Hospital, St Mary's Hospital and Hammersmith Hospital, London, UK
[88]Dumfries and Galloway Royal Infirmary, Dumfries, UK
[89]Bristol Royal Infirmary, Bristol, UK
[90]Royal Sussex County Hospital, Brighton, UK

[91]Whiston Hospital, Prescot, UK
[92]Royal Glamorgan Hospital, Cardiff, UK
[93]King's Mill Hospital, Nottingham, UK
[94]Fairfield General Hospital, Bury, UK
[95]Western General Hospital, Edinburgh, UK
[96]Northwick Park Hospital, London, UK
[97]Royal Preston Hospital, Preston, UK
[98]Royal Derby Hospital, Derby, UK
[99]Sunderland Royal Hospital, Sunderland, UK
[100]Royal Surrey County Hospital, Guildford, UK
[101]Derriford Hospital, Plymouth, UK
[102]Croydon University Hospital, Croydon, UK
[103]Victoria Hospital, Kirkcaldy, UK
[104]Milton Keynes University Hospital, Milton Keynes, UK
[105]Barnsley Hospital, Barnsley, UK
[106]York Hospital, York, UK
[107]University Hospital of North Tees, Stockton on Tees, UK
[108]University Hospital Wishaw, Wishaw, UK
[109]Whittington Hospital, London, UK
[110]Southmead Hospital, Bristol, UK
[111]The Royal Papworth Hospital, Cambridge, UK
[112]Royal Gwent Hospital, Newport, UK
[113]Norfolk and Norwich University hospital (NNUH), Norwich, UK
[114]Great Ormond St Hospital and UCL Great Ormond St Institute of Child Health NIHR Biomedical Research Centre, London, UK
[115]Airedale General Hospital, Keighley, UK
[116]Aberdeen Royal Infirmary, Aberdeen, UK
[117]Southampton General Hospital, Southampton, UK
[118]Russell's Hall Hospital, Dudley, UK
[119]Rotherham General Hospital, Rotherham, UK
[120]North Manchester General Hospital, Manchester, UK
[121]Basingstoke and North Hampshire Hospital, Basingstoke, UK
[122]Royal Free Hospital, London, UK
[123]Hull Royal Infirmary, Hull, UK
[124]Harefield Hospital, London, UK
[125]Chesterfield Royal Hospital Foundation Trust, Chesterfield, UK
[126]Barnet Hospital, London, UK
[127]Aintree University Hospital, Liverpool, UK
[128]St James's University Hospital and Leeds General Infirmary, Leeds, UK
[129]Glan Clwyd Hospital, Bodelwyddan, UK
[130]University Hospital Crosshouse, Kilmarnock, UK
[131]Royal Bolton Hospital, Bolton, UK
[132]Princess of Wales Hospital, Llantrisant, UK
[133]Pilgrim Hospital, Lincoln, UK
[134]Northumbria Healthcare NHS Foundation Trust, North Shields, UK
[135]Ninewells Hospital, Dundee, UK

[136]Lister Hospital, Stevenage, UK
[137]Bedford Hospital, Bedford, UK
[138]Royal United Hospital, Bath, UK
[139]Royal Bournemouth Hospital, Bournemouth, UK
[140]The Great Western Hospital, Swindon, UK
[141]Watford General Hospital, Watford, UK
[142]University Hospital North Durham, Darlington, UK
[143]Tameside General Hospital, Ashton Under Lyne, UK
[144]Princess Royal Hospital Shrewsbury and Royal Shrewsbury Hospital, Shrewsbury, UK
[145]Arrowe Park Hospital, Wirral, UK
[146]The Queen Elizabeth Hospital, King's Lynn, UK
[147]Royal Blackburn Teaching Hospital, Blackburn, UK
[148]Poole Hospital, Poole, UK
[149]Medway Maritime Hospital, Gillingham, UK
[150]Warwick Hospital, Warwick, UK
[151]The Royal Marsden Hospital, London, UK
[152]The Princess Alexandra Hospital, Harlow, UK
[153]Musgrove Park Hospital, Taunton, UK
[154]George Eliot Hospital NHS Trust, Nuneaton, UK
[155]East Surrey Hospital, Redhill, UK
[156]West Middlesex Hospital, Isleworth, UK
[157]Warrington General Hospital, Warrington, UK
[158]Southport and Formby District General Hospital, Ormskirk, UK
[159]Royal Devon and Exeter Hospital, Exeter, UK
[160]Macclesfield District General Hospital, Macclesfield, UK
[161]Borders General Hospital, Melrose, UK
[162]Birmingham Children's Hospital, Birmingham, UK
[163]William Harvey Hospital, Ashford, UK
[164]Royal Lancaster Infirmary, Lancaster, UK
[165]Queen Elizabeth the Queen Mother Hospital, Margate, UK
[166]Liverpool Heart and Chest Hospital, Liverpool, UK
[167]Darlington Memorial Hospital, Darlington, UK
[168]Southend University Hospital, Westcliff-on-Sea, UK
[169]Raigmore Hospital, Inverness, UK
[170]Salisbury District Hospital, Salisbury, UK
[171]Peterborough City Hospital, Peterborough, UK
[172]Ipswich Hospital, Ipswich, UK
[173]Hereford County Hospital, Worcester, UK
[174]Furness General Hospital, Barrow-in-Furness, UK
[175]Forth Valley Royal Hospital, Falkirk, UK
[176]Torbay Hospital, Torquay, UK
[177]St Mary's Hospital, Newport, UK
[178]Royal Manchester Children's Hospital, Manchester, UK
[179]Royal Cornwall Hospital, Truro, UK
[180]Queen Elizabeth Hospital Gateshead, Gateshead, UK
[181]Kent & Canterbury Hospital, Canterbury, UK

[182]James Paget University Hospital NHS Trust, Great Yarmouth, UK
[183]Darent Valley Hospital, Dartford, UK
[184]The Alexandra Hospital, Redditch and Worcester Royal Hospital, Worcester, UK
[185]Ysbyty Gwynedd, Bangor, UK
[186]Yeovil Hospital, Yeovil, UK
[187]University Hospital Hairmyres, East Kilbride, UK
[188]Scunthorpe General Hospital, Scunthorpe, UK
[189]Princess Royal Hospital Brighton, West Sussex, UK
[190]Lincoln County Hospital, Lincoln, UK
[191]Homerton University Hospital, London, UK
[192]Glangwili General Hospital, Camarthen, UK
[193]Ealing Hospital, Southall, UK
[194]Scarborough General Hospital, Scarborough, UK
[195]Royal Albert Edward Infirmary, Wigan, UK
[196]Queen Elizabeth Hospital, Woolwich, London, UK
[197]North Devon District Hospital, Barnstaple, UK
[198]National Hospital for Neurology and Neurosurgery, London, UK
[199]Eastbourne District General Hospital, East Sussex, UK and Conquest Hospital, East Sussex, UK
[200]Diana Princess of Wales Hospital, Grimsby, UK
[201]The Christie NHS Foundation Trust, Manchester, UK
[202]Prince Philip Hospital, Lianelli, UK
[203]Prince Charles Hospital, Merthyr Tydfil, UK
[204]Golden Jubilee National Hospital, Clydebank, UK
[205]Dorset County Hospital, Dorchester, UK
[206]Calderdale Royal Hospital, Halifax, UK
[207]West Suffolk Hospital, Suffolk, UK
[208]West Cumberland Hospital, Whitehaven, UK
[209]University Hospital Lewisham, London, UK
[210]St John's Hospital Livingston, Livingston, UK
[211]Sheffield Children's Hospital, Sheffield, UK
[212]Hinchingbrooke Hospital, Huntingdon, UK
[213]Glenfield Hospital, Leicester, UK
[214]Bronglais General Hospital, Aberystwyth, UK
[215]Alder Hey Children's Hospital, Liverpool, UK
[216]University Hospital Monklands, Airdrie, UK
[217]Cumberland Infirmary, Carlisle, UK
